# Supplementary material for: Upfront admixing antibodies and EGFR inhibitors preempts sequential treatments in lung cancer models
Source: EMBO Mol Med. 2021 Mar 4;13(4):e13144. doi: 10.15252/emmm.202013144 (PMC8033519; doi:10.15252/emmm.202013144)
Supplement: Supplementary file 1 — Appendix [file EMMM-13-e13144-s002.pdf]

APPENDIX

Upfront admixing antibodies and EGFR inhibitors preempts sequential treatments in lung cancer models

Ilaria Marrocco, Donatella Romaniello, Itay Vaknin, Diana Drago-Garcia, Roni Oren, Mary Luz Uribe, Nishanth Belugali Nataraj, Soma Ghosh, Raya Eilam, Tomer Meir Salame, Moshit Lindzen and Yosef Yarden

Table of contents:

- **Appendix Figure S1.** Combinations of antibodies and TKIs increase apoptosis of PC9 and H3255 NSCLC cells.
- **Appendix Figure S2.** Combinations of EGFR-specific TKIs and two monoclonal antibodies (cetuximab and trastuzumab) induce cell cycle arrest and strongly inhibit proliferation of PC9 cells.
- **Appendix Figure S3.** Adding two monoclonal antibodies (cetuximab and trastuzumab) to an EGFR-specific TKI either cures or significantly delays relapses of an EGFR-mutated xenograft model of NSCLC.
- **Appendix Figures S4.** Combinations of antibodies and TKIs increase apoptosis and decrease the levels of the proliferation marker Ki67 in vivo.
- **Appendix Figure S5.** Sequential treatments with a TKI, which is followed, after relapse, by two monoclonal antibodies, variably affect tumor growth in an animal model.
- **Appendix Figure S6.** By downregulating EGFR, HER2 and additional RTKs, a combination of two antibodies and erlotinib overcomes drug resistance in an animal model.
- **Appendix Figure S7.** Combining monoclonal antibodies (cetuximab and trastuzumab) with EGFR-TKIs induces no evident effects on body weight of treated animals.
- **Appendix Table S1:** List of primers used for RT-PCR.
- **Appendix Table S2:** Statistical parameters corresponding to individual figures.

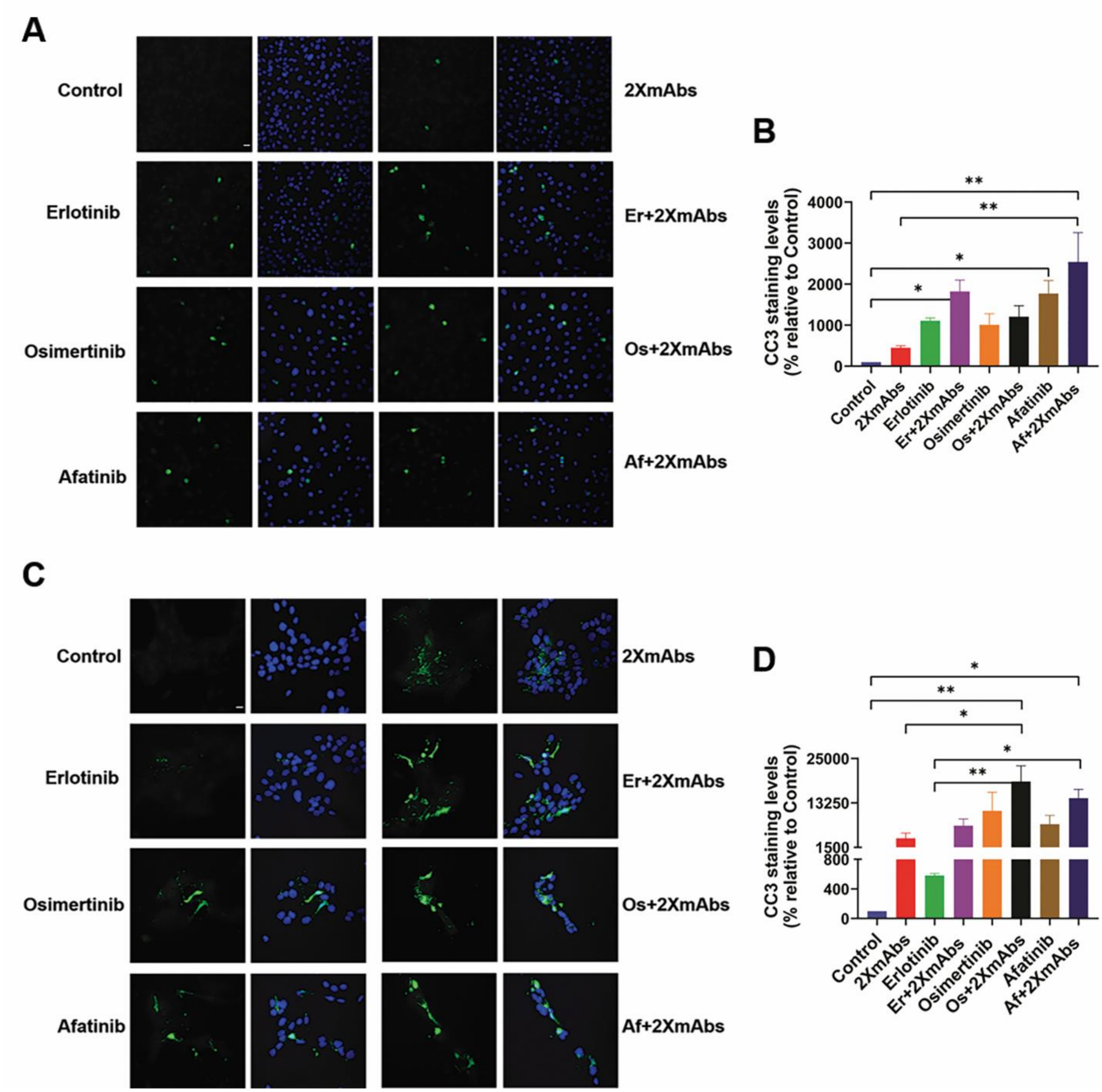

Appendix Figure S1. Combinations of antibodies and TKIs increase apoptosis of PC9 and H3255 NSCLC cells. PC9 (**A**, **B**) or H3255 (**C**, **D**) cells were seeded on coverslips and treated for 48 hours with different EGFR-specific TKIs (erlotinib, 40 nM; osimertinib, 40 nM, or afatinib, 10 nM), either alone or in combination with 2XmAbs (cetuximab and trastuzumab, 5 μg/ml, each). Cells were fixed in paraformaldehyde (4%) and incubated with an anti-cleaved caspase 3 antibody (CC3), followed by an Alexa Fluor 488-conjugated secondary antibody. DAPI (blue) was used to stain nuclei. Images were captured using confocal microscopy (20X magnification) (**A** and **C**). Bar, 20 μm. CC3 staining was quantified using Fiji and normalized to the nuclei, and results are

presented as means + SEM of three experiments. (**B** and **D**) Significance was assessed using one-way ANOVA followed by Tukey's multiple comparisons test. Note that non-significant comparisons are not shown. See a list of p-values in Appendix Table S2.

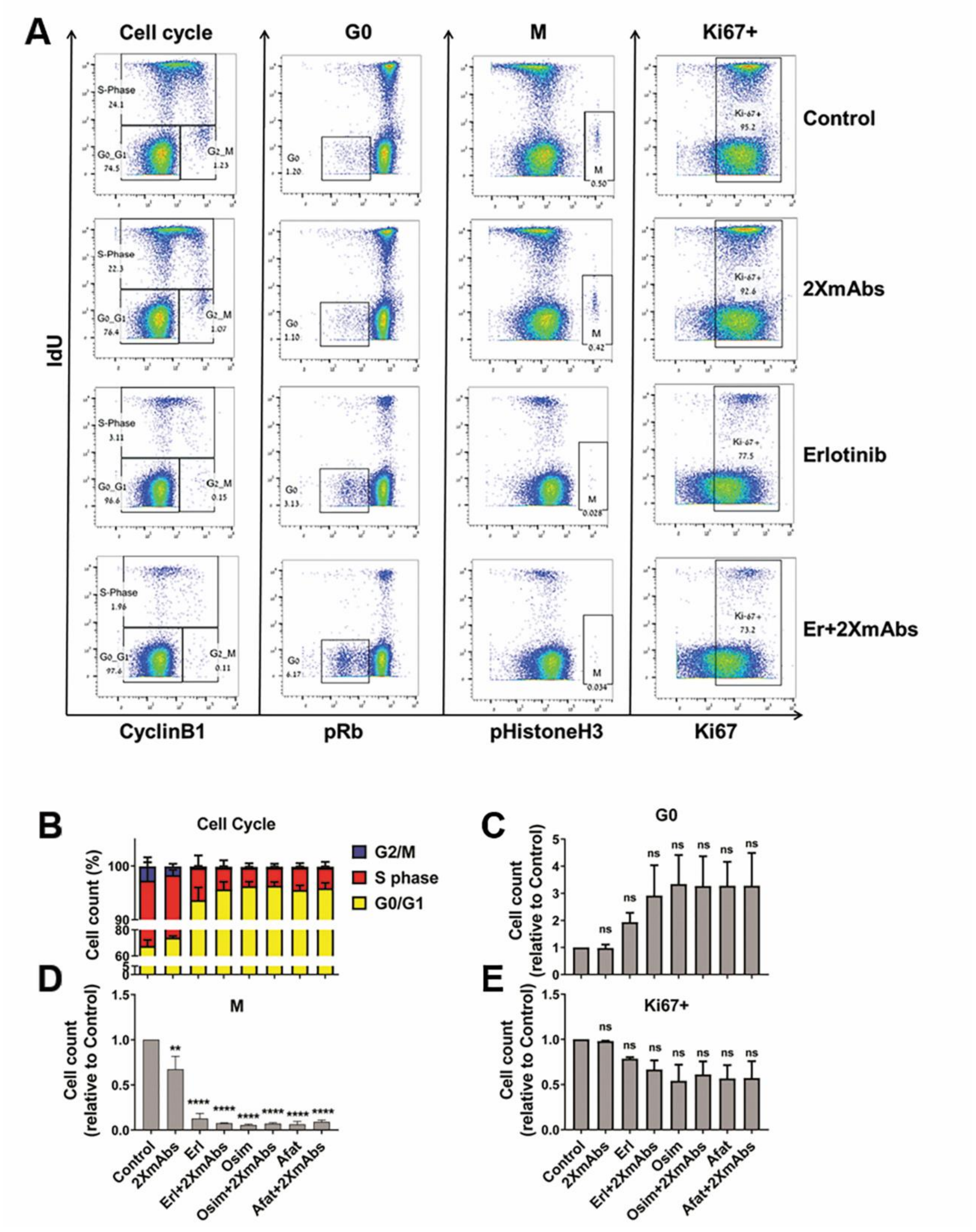

Appendix Figure S2. Combinations of EGFR-specific TKIs and two monoclonal antibodies (cetuximab and trastuzumab) induce cell cycle arrest and strongly inhibit proliferation of PC9 cells. (**A**) PC9 cells were seeded in 10-cm dishes and treated for 48 hours, in media containing 1% serum, with erlotinib (40 nM), osimertinib (40 nM) or afatinib (10 nM), either alone or in combination with 2XmAbs (cetuximab and trastuzumab, each at 5 μg/ml). Thereafter, the cells were exposed to IdU (5-Iodo-2′-deoxyuridine) for 2 hours, followed by incubation with metal-conjugated primary antibodies included in the Maxpar Cell Cycle Panel Kit (Fluidigm). Samples were analyzed using the CyTOF 2 mass cytometer (Fluidigm). The figure presents only the results we obtained with erlotinib and 2XmAbs. (**B-E**) The percentages of cells in G0/G1, S or G2/M phases of the cell cycle were estimated according to the levels of cyclinB1. Antibodies against pRB and phosphorylated histone H3 were used to assay the fractions of cells found in G0 and M phases, respectively. Signal quantification of the cells in each phase is presented. The fraction of cells found to be in the active proliferation state are presented as $Ki67^+$ cells. Data were analyzed using the FlowJo software and the results from three experiments are presented as mean + SEM. Significance was assessed using one-way ANOVA followed by Dunnett's multiple comparisons test. See a list of p-values in Appendix Table S2.

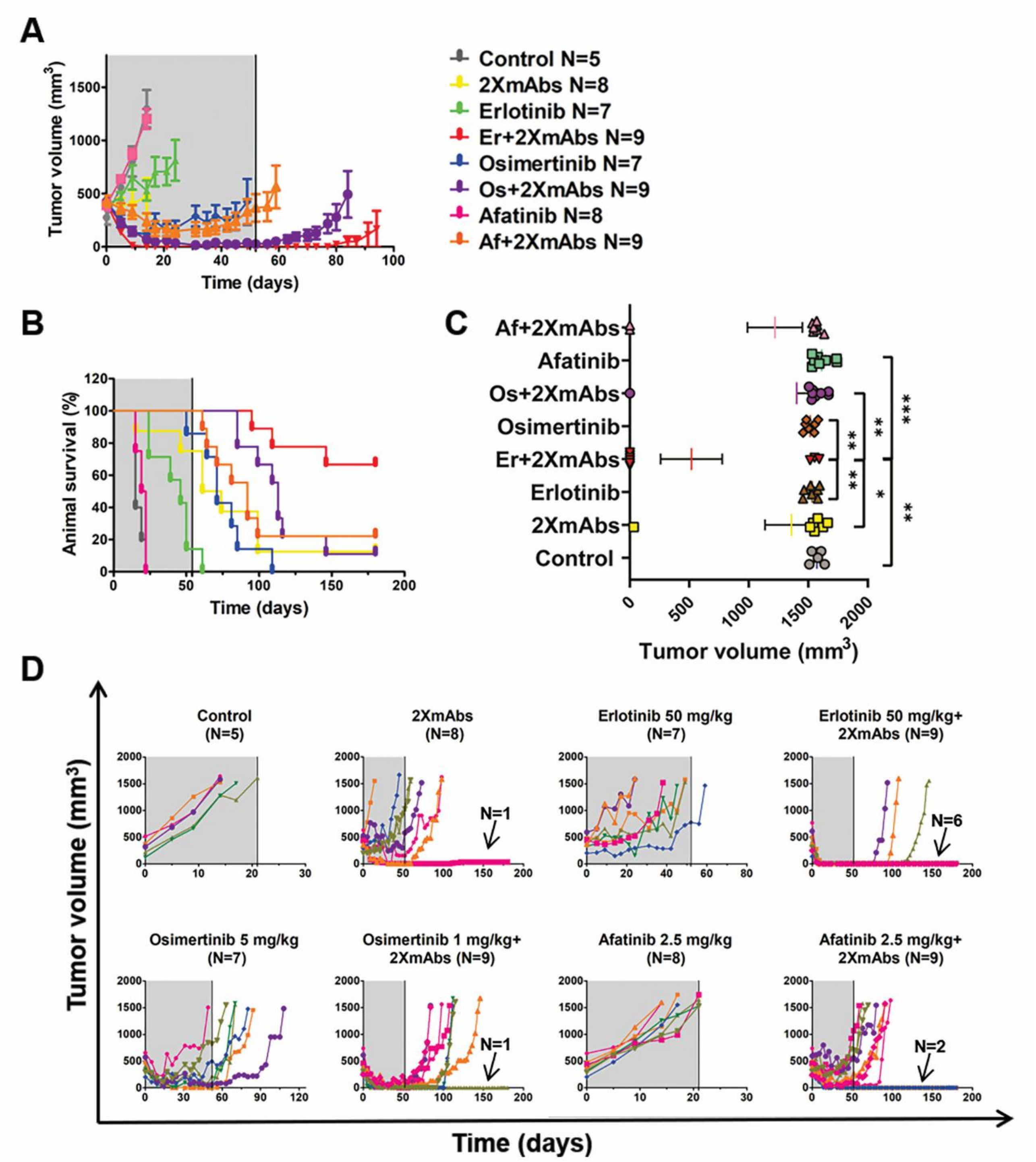

Appendix Figure S3. Adding two monoclonal antibodies (cetuximab and trastuzumab) to an EGFR-specific TKI either cures or significantly delays relapses of an EGFR-mutated xenograft model of NSCLC. (**A** and **B**) PC9 cells (exon 19 deletion) were subcutaneously implanted in the flanks of CD1-nu/nu mice ($3X10^6$/mouse). Once tumors became palpable, mice were randomized to 8 groups of 5-9 animals and treated, once every three days, with 2XmAbs

(cetuximab and trastuzumab, each at 0.1 mg/mouse/injection), the following TKIs: erlotinib (50 mg/kg/day), afatinib (2.5 mg/kg) or osimertinib (5 mg/kg), or the respective combinations of TKIs and antibodies. Note that the dose of osimertinib in the combination group was reduced to 1 mg/kg/day. Treatments were stopped on day 54 (21 days for the afatinib group). Tumor volumes (panel A) were followed for 23 additional days, and animal survival (panel B) was followed for 126 additional days. Mice were euthanized when tumor size reached 1,500 mm^3. Data are means ± SEM from 5-9 mice per group. (**C**) Statistical analysis of tumor volumes corresponding to the last measurement for each mouse was performed using one-way ANOVA followed by Tukey's multiple comparison test. Note that only significant comparisons are shown. See a list of p-values in Appendix Table S2. (**D**) Tumor volumes of individual mice in each group are shown in different colors. The numbers (N) of tumor-free mice in each group are indicated.

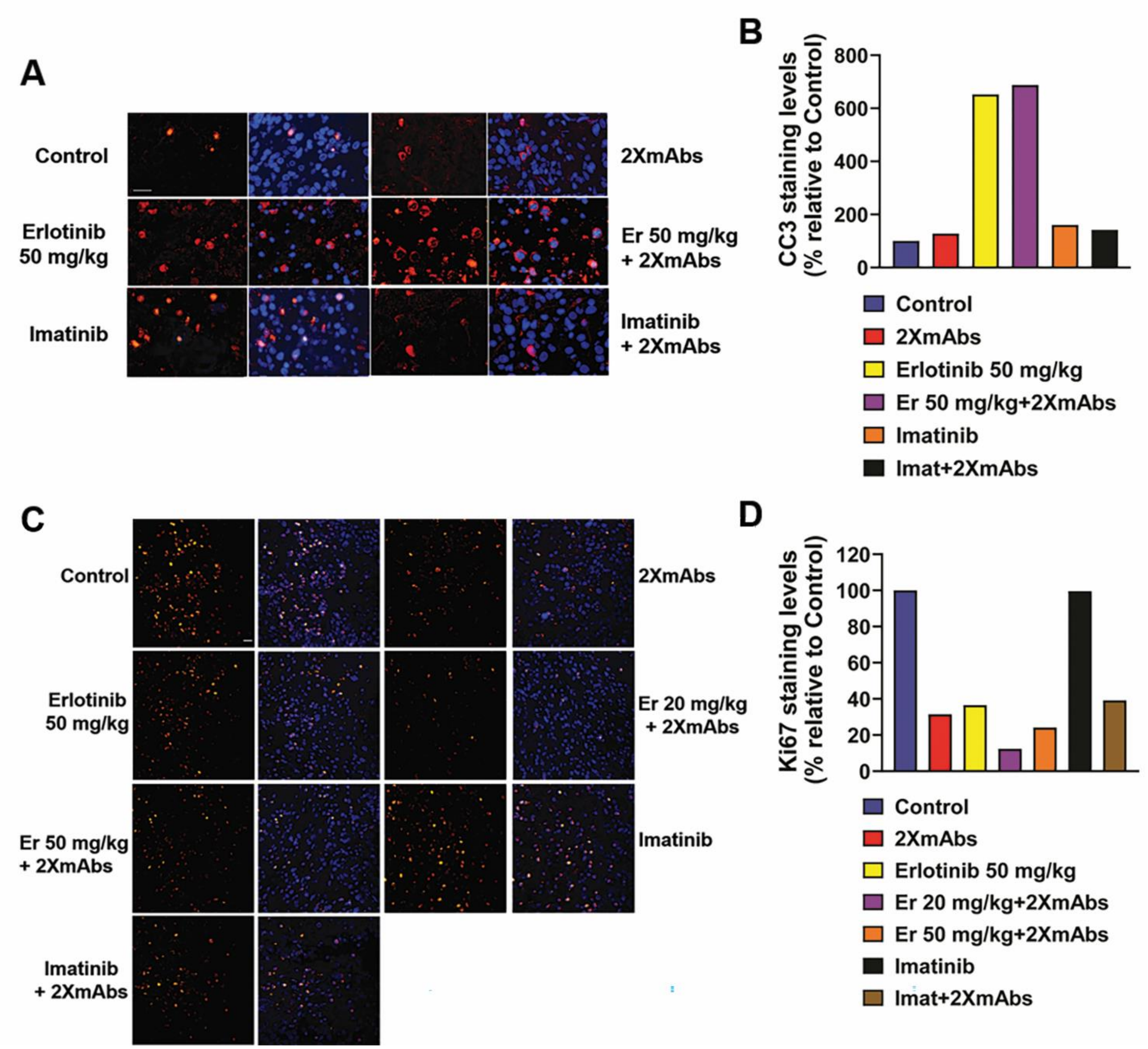

Appendix Figures S4. Combinations of antibodies and TKIs increase apoptosis and decrease the levels of the proliferation marker Ki67 in vivo. Mice were treated as in Figures 4 and EV4. Sections (4 µm) were obtained from the following tumors shown in Figure 4 and EV4: Control - mouse 3, 2XmAbs - mouse 1, erlotinib - mouse 2, erlotinib 20+2XmAbs - mouse 2, erlotinib 50+2XmAbs - mouse 2 (all from Figure 4), and both imatinib - mouse 3 and imatinib+2XmAbs - mouse 1 (both from Figure EV4). Tumor sections were analyzed by means of immunofluorescence analysis, which utilized an anti-cleaved caspase 3 (CC3) antibody and a Cy3-secondary antibody (pseudo colored in red). Images were captured using a fluorescence microscope (24X magnification). Bar, 50 µm (**A**). In (**C**), tumor sections were stained with an anti-Ki67 antibody

followed by a Cy3-secondary antibody. Images were captured using a fluorescence microscope (20X magnification). Bar, 20 µm. DAPI was used to stain nuclei. CC3-positive or Ki67 positive cells were counted using the Fiji software, and the relative histograms are shown in (**B**) and (**D**), respectively.

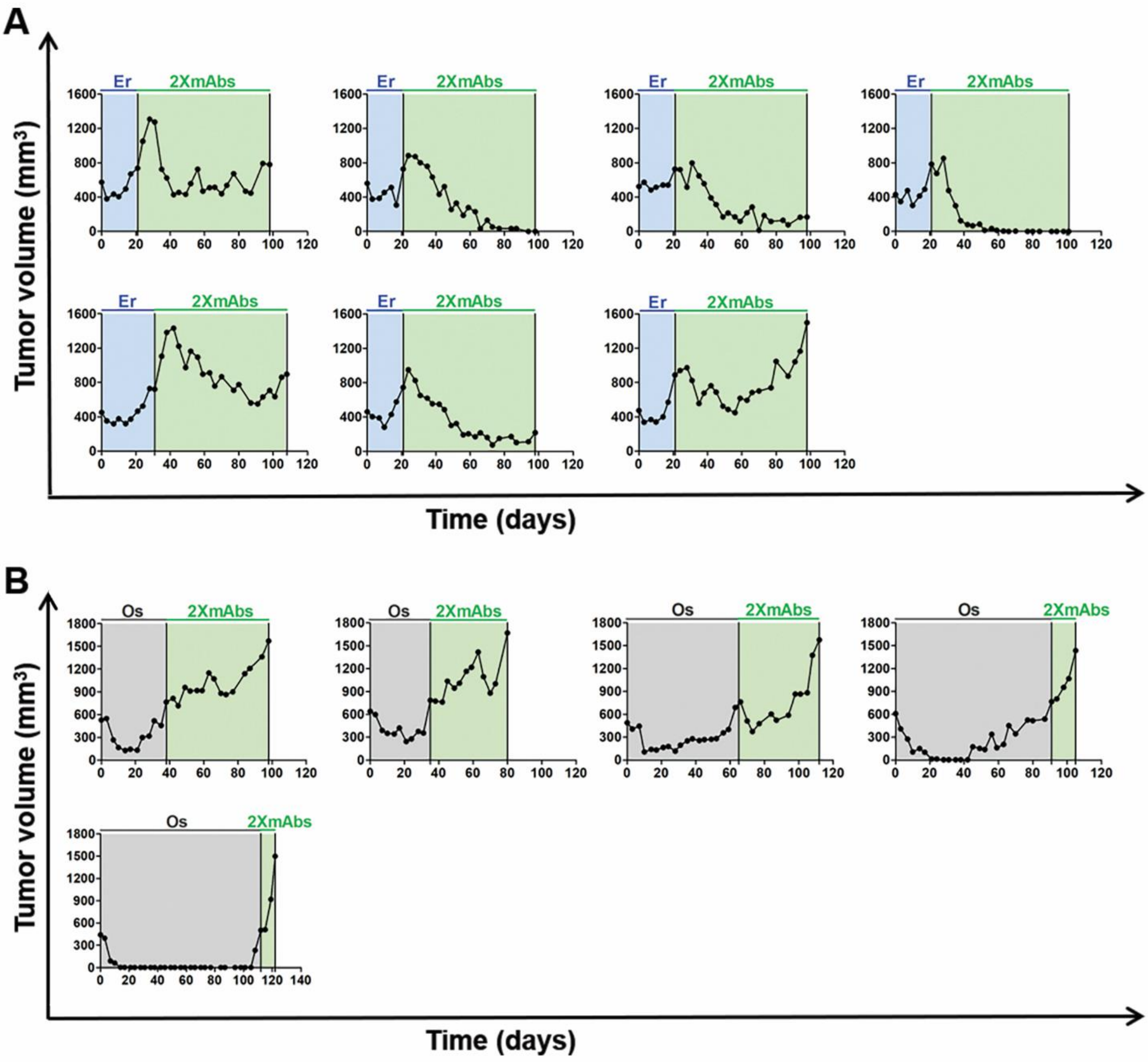

Appendix Figure S5. Sequential treatments with a TKI, which is followed, after relapse, by two monoclonal antibodies, variably affect tumor growth in an animal model. CD1-nu/nu mice bearing PC9 xenografts, were orally treated daily with erlotinib (50 mg/kg, blue area; panel **A**) or osimertinib (5 mg/kg, grey area; panel **B**). Once tumor relapsed under erlotinib or osimertinib

and reached 800 mm^3, mice were treated twice a week with 2XmAbs (cetuximab and trastuzumab, each at 0.1 mg/mouse/injection; green area) in the absence of TKIs. Each panel corresponds to an individual animal.

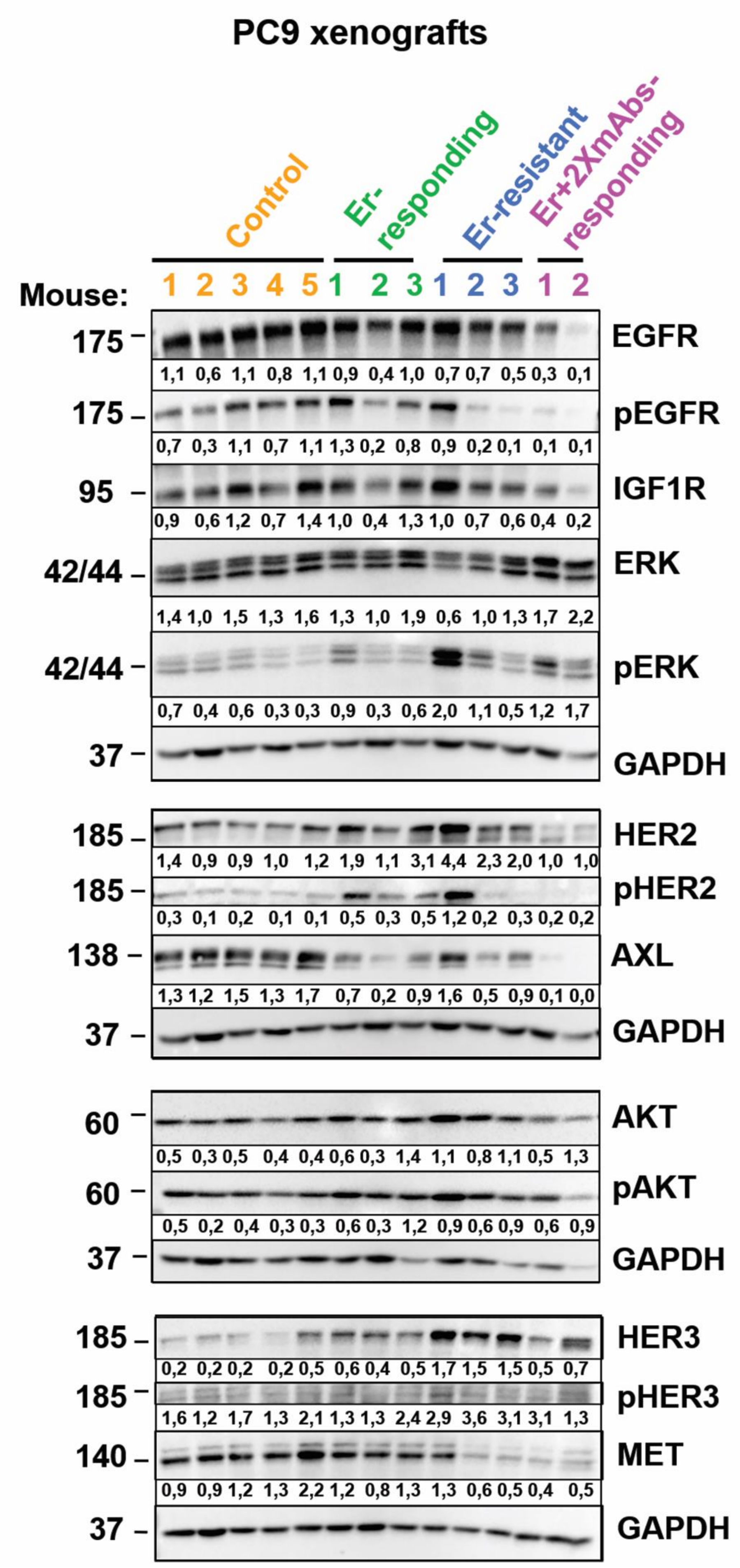
PC9 xenografts
Control
Er-responding
Er-resistant
Er+2XmAbs-responding
Mouse: 1 2 3 4 5 1 2 3 1 2 3 1 2
175
EGFR
1,1 0,6 1,1 0,8 1,1 0,9 0,4 1,0 0,7 0,7 0,5 0,3 0,1
175
pEGFR
0,7 0,3 1,1 0,7 1,1 1,3 0,2 0,8 0,9 0,2 0,1 0,1 0,1
95
IGF1R
0,9 0,6 1,2 0,7 1,4 1,0 0,4 1,3 1,0 0,7 0,6 0,4 0,2
42/44
ERK
1,4 1,0 1,5 1,3 1,6 1,3 1,0 1,9 0,6 1,0 1,3 1,7 2,2
42/44
pERK
0,7 0,4 0,6 0,3 0,3 0,9 0,3 0,6 2,0 1,1 0,5 1,2 1,7
37
GAPDH
185
HER2
1,4 0,9 0,9 1,0 1,2 1,9 1,1 3,1 4,4 2,3 2,0 1,0 1,0
185
pHER2
0,3 0,1 0,2 0,1 0,1 0,5 0,3 0,5 1,2 0,2 0,3 0,2 0,2
138
AXL
1,3 1,2 1,5 1,3 1,7 0,7 0,2 0,9 1,6 0,5 0,9 0,1 0,0
37
GAPDH
60
AKT
0,5 0,3 0,5 0,4 0,4 0,6 0,3 1,4 1,1 0,8 1,1 0,5 1,3
60
pAKT
0,5 0,2 0,4 0,3 0,3 0,6 0,3 1,2 0,9 0,6 0,9 0,6 0,9
37
GAPDH
185
HER3
0,2 0,2 0,2 0,2 0,5 0,6 0,4 0,5 1,7 1,5 1,5 0,5 0,7
185
pHER3
1,6 1,2 1,7 1,3 2,1 1,3 1,3 2,4 2,9 3,6 3,1 3,1 1,3
140
MET
0,9 0,9 1,2 1,3 2,2 1,2 0,8 1,3 1,3 0,6 0,5 0,4 0,5
37
GAPDH

Appendix Figure S6. By downregulating EGFR, HER2 and additional RTKs, a combination of two antibodies and erlotinib overcomes drug resistance in an animal model. Protein extracts were prepared from the tumors presented in Figure 7. The extracts were analyzed using immunoblotting. Signals were quantified and normalized to the signals corresponding to GAPDH. Numerical signals are shown below each lane.

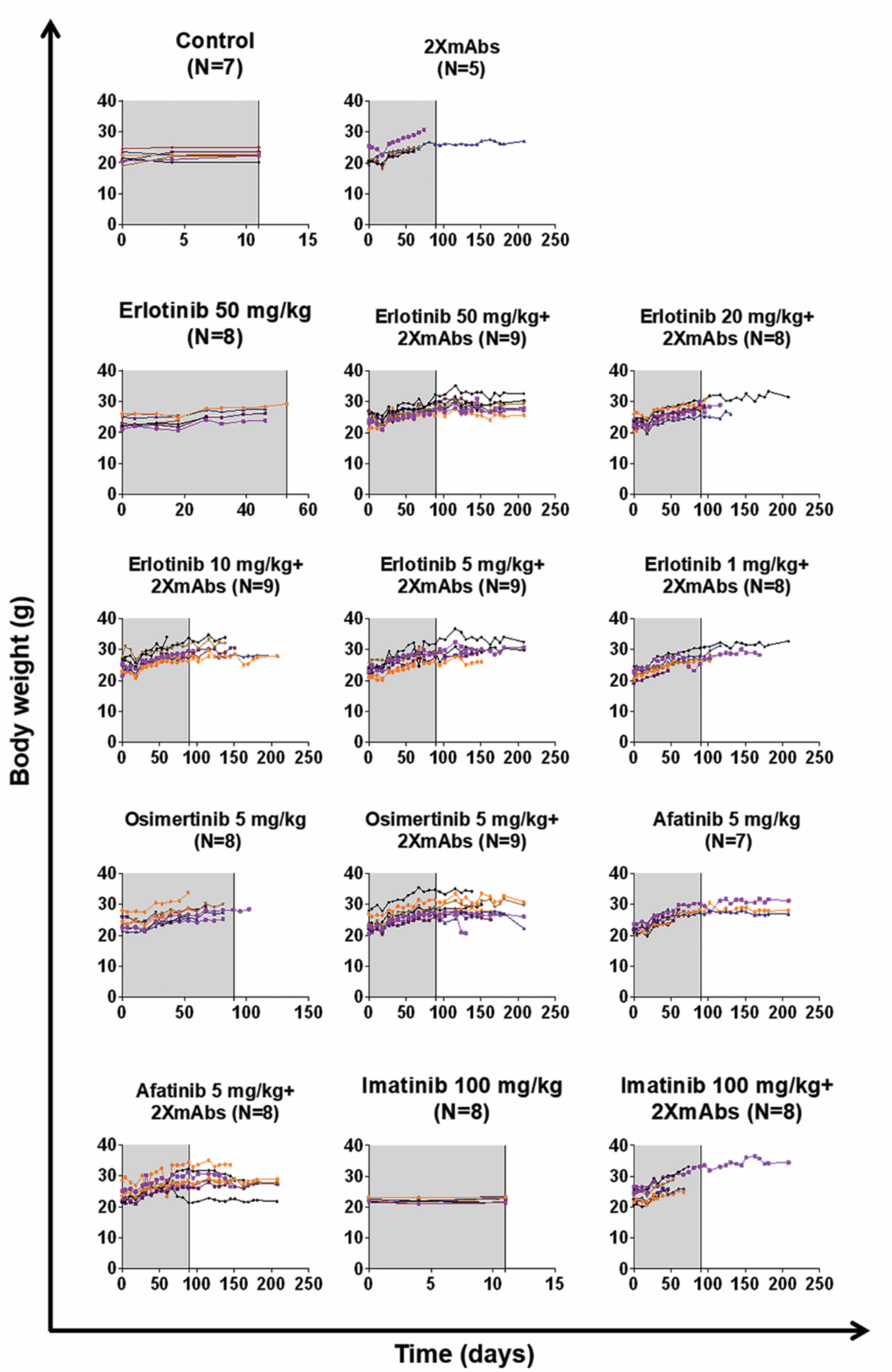

Appendix Figure S7. Combining monoclonal antibodies (cetuximab and trastuzumab) with EGFR-TKIs induces no evident effects on body weight of treated animals. PC9 cells (exon 19 deletion) were subcutaneously implanted in the flank of CD1-nu/nu mice ($3X10^6$/mouse). When tumors became palpable, mice were randomized into groups of 5-9 animals and treated for 90 days (grey areas) as described in figures 2 and 3. Body weight was measured once a week.

Appendix Table S1: List of primers used for RT-PCR.

Gene name	Forward primer	Reverse primer
FOXM1	CGTCGGCCACTGATTCTCAAA	GGCAGGGGATCTCTTAGGTTC
AURKA	GGAATATGCACCACTTGGAACA	TAAGACAGGGCATTTGCCAAT
TPX2	ATGGAACTGGAGGGCTTTTTC	TGTTGTCAACTGGTTTCAAAGGT
CENPA	AGCACACACCTCTTGATAAGGA	CACACCACGAGTGAATTTAACAC
CCNB1	AACTTTCGCCTGAGCCTATTTT	TTGGTCTGACTGCTTGCTCTT
BIRC5	AGGACCACCGCATCTCTACAT	AAGTCTGGCTCGTTCTCAGTG
PRC1	TAGACCACACCCCAGACACAAG	CCCCTCACACACTGCTTCATT
KIF4A	TGCAGCCCATTCAGTACCAA	GCGCTCACTCAACTTGGCTT
GAPDH	GGGTCATTGATGGCAAC	GAAGGTGAAGGTCGGA

Appendix Table S2: Statistical parameters corresponding to individual figures.

Figure	Groups	Symbol	p-Value	N1	N2
**1B_EGFR**	Control vs. 2XmAbs	**	0,0091	4	4
	Control vs. Erlotinib	ns	0,7136	4	4
	Control vs. Er+2XmAbs	**	0,0064	4	4
	Control vs. Osimertinib	ns	0,6805	4	4
	Control vs. Os+2XmAbs	**	0,002	4	4
	Control vs. Afatinib	ns	0,592	4	4
	Control vs. Af+2XmAbs	**	0,0031	4	4
**1B_HER2**	Control vs. 2XmAbs	ns	0,9979	4	4
	Control vs. Erlotinib	**	0,0015	4	4
	Control vs. Er+2XmAbs	ns	>0,9999	4	4
	Control vs. Osimertinib	ns	0,1342	4	4
	Control vs. Os+2XmAbs	ns	0,9999	4	4
	Control vs. Afatinib	*	0,0355	4	4
	Control vs. Af+2XmAbs	ns	0,9998	4	4
**1B_HER3**	Control vs. 2XmAbs	****	<0,0001	4	4
	Control vs. Erlotinib	****	<0,0001	4	4
	Control vs. Er+2XmAbs	****	<0,0001	4	4
	Control vs. Osimertinib	****	<0,0001	4	4
	Control vs. Os+2XmAbs	****	<0,0001	4	4
	Control vs. Afatinib	****	<0,0001	4	4
	Control vs. Af+2XmAbs	****	<0,0001	4	4
	Control vs. 2XmAbs	ns	0,9541	7	5
	Control vs. Erlotinib 50 mg/kg	ns	>0,9999	7	8
	Control vs. Er 50 mg/kg+2XmAbs	****	<0,0001	7	9
	Control vs. Er 20mg/kg+2XmAbs	ns	0,9966	7	8
	Control vs. Er 10 mg/kg+2XmAbs	ns	0,8969	7	9

	Control vs. Er 5 mg/kg+2XmAbs	ns	0,2468	7	9
	Control vs. Er 1 mg/kg+2XmAbs	ns	0,9893	7	8
	2XmAbs vs. Erlotinib 50 mg/kg	ns	0,9842	5	8
	2XmAbs vs. Er 50 mg/kg+2XmAbs	**	0,002	5	9
	2XmAbs vs. Er 20mg/kg+2XmAbs	ns	0,9997	5	8
	2XmAbs vs. Er 10 mg/kg+2XmAbs	ns	>0,9999	5	9
	2XmAbs vs. Er 5 mg/kg+2XmAbs	ns	0,9655	5	9
	2XmAbs vs. Er 1 mg/kg+2XmAbs	ns	>0,9999	5	8
**2C**	Erlotinib 50 mg/kg vs. Er 50 mg/kg+2XmAbs	****	<0,0001	8	9
	Erlotinib 50 mg/kg vs. Er 20mg/kg+2XmAbs	ns	0,9997	8	8
	Erlotinib 50 mg/kg vs. Er 10 mg/kg+2XmAbs	ns	0,9582	8	9
	Erlotinib 50 mg/kg vs. Er 5 mg/kg+2XmAbs	ns	0,3336	8	9
	Erlotinib 50 mg/kg vs. Er 1 mg/kg+2XmAbs	ns	0,9984	8	8
	Er 50 mg/kg+2XmAbs vs. Er 20mg/kg+2XmAbs	****	<0,0001	9	8
	Er 50 mg/kg+2XmAbs vs. Er 10 mg/kg+2XmAbs	***	0,0002	9	9
	Er 50 mg/kg+2XmAbs vs. Er 5 mg/kg+2XmAbs	**	0,0082	9	9
	Er 50 mg/kg+2XmAbs vs. Er 1 mg/kg+2XmAbs	****	<0,0001	9	8
	Er 20mg/kg+2XmAbs vs. Er 10 mg/kg+2XmAbs	ns	0,9988	8	9
	Er 20mg/kg+2XmAbs vs. Er 5 mg/kg+2XmAbs	ns	0,6399	8	9
	Er 20mg/kg+2XmAbs vs. Er 1 mg/kg+2XmAbs	ns	>0,9999	8	8
	Er 10 mg/kg+2XmAbs vs. Er 5 mg/kg+2XmAbs	ns	0,9237	9	9
	Er 10 mg/kg+2XmAbs vs. Er 1 mg/kg+2XmAbs	ns	0,9998	9	8
	Er 5 mg/kg+2XmAbs vs. Er 1 mg/kg+2XmAbs	ns	0,7368	9	8
	Control vs. 2XmAbs	ns	0,9593	7	5
	Control vs. Osimertinib	ns	0,9989	7	8
	Control vs. Os+2XmAbs	***	0,0001	7	9
	Control vs. Afatinib	ns	0,2511	7	7
	Control vs. Af+2XmAbs	**	0,0014	7	8
	Control vs. Imatinib	ns	>0,9999	7	8
	Control vs. Imat+2XmAbs	ns	0,99	7	8
	2XmAbs vs. Osimertinib	ns	0,9991	5	8
	2XmAbs vs. Os+2XmAbs	*	0,0241	5	9
	2XmAbs vs. Afatinib	ns	0,9447	5	7
	2XmAbs vs. Af+2XmAbs	ns	0,0972	5	8
	2XmAbs vs. Imatinib	ns	0,9289	5	8
	2XmAbs vs. Imat+2XmAbs	ns	>0,9999	5	8
	Osimertinib vs. Os+2XmAbs	***	0,0007	8	9
**3C**	Osimertinib vs. Afatinib	ns	0,5475	8	7
	Osimertinib vs. Af+2XmAbs	**	0,0057	8	8
	Osimertinib vs. Imatinib	ns	0,996	8	8

	Osimertinib vs. Imat+2XmAbs	ns	>0,9999	8	8
	Os+2XmAbs vs. Afatinib	ns	0,2287	9	7
	Os+2XmAbs vs. Af+2XmAbs	ns	0,9992	9	8
	Os+2XmAbs vs. Imatinib	****	<0,0001	9	8
	Os+2XmAbs vs. Imat+2XmAbs	**	0,0016	9	8
	Afatinib vs. Af+2XmAbs	ns	0,5664	7	8
	Afatinib vs. Imatinib	ns	0,1761	7	8
	Afatinib vs. Imat+2XmAbs	ns	0,7075	7	8
	Af+2XmAbs vs. Imatinib	***	0,0006	8	8
	Af+2XmAbs vs. Imat+2XmAbs	*	0,0121	8	8
	Imatinib vs. Imat+2XmAbs	ns	0,9761	8	8
	Control vs. 2XmAbs	ns	0,4834	4	7
	Control vs. Erlotinib	ns	0,9978	4	8
	Control vs. Er+2XmAbs	**	0,0034	4	10
	Control vs. Osimertinib	ns	0,3583	4	10
	Control vs. Os+2XmAbs	**	0,0084	4	10
	2XmAbs vs. Erlotinib	ns	0,5742	7	8
	2XmAbs vs. Er+2XmAbs	ns	0,1478	7	10
**EV3-C**	2XmAbs vs. Osimertinib	ns	>0,9999	7	10
	2XmAbs vs. Os+2XmAbs	ns	0,2976	7	10
	Erlotinib vs. Er+2XmAbs	***	0,001	8	10
	Erlotinib vs. Osimertinib	ns	0,3999	8	10
	Erlotinib vs. Os+2XmAbs	**	0,0032	8	10
	Er+2XmAbs vs. Osimertinib	ns	0,1166	10	10
	Er+2XmAbs vs. Os+2XmAbs	ns	0,9983	10	10
	Osimertinib vs. Os+2XmAbs	ns	0,2619	10	10
	Control vs. 2XmAbs	ns	0,9926	3	3
	Control vs. Erlotinib	ns	0,403	3	3
	Control vs. Er+2XmAbs	*	0,0292	3	3
	Control vs. Osimertinib	ns	0,5251	3	3
	Control vs. Os+2XmAbs	ns	0,3031	3	3
	Control vs. Afatinib	*	0,0359	3	3
	Control vs. Af+2XmAbs	**	0,0014	3	3
	2XmAbs vs. Erlotinib	ns	0,8328	3	3
	2XmAbs vs. Er+2XmAbs	ns	0,1203	3	3
	2XmAbs vs. Osimertinib	ns	0,9185	3	3
	2XmAbs vs. Os+2XmAbs	ns	0,7255	3	3
	2XmAbs vs. Afatinib	ns	0,1451	3	3
	2XmAbs vs. Af+2XmAbs	**	0,0062	3	3
**Appendix S1B**	Erlotinib vs. Er+2XmAbs	ns	0,7702	3	3

	Erlotinib vs. Osimertinib	ns	>0,9999	3	3
	Erlotinib vs. Os+2XmAbs	ns	>0,9999	3	3
	Erlotinib vs. Afatinib	ns	0,8252	3	3
	Erlotinib vs. Af+2XmAbs	ns	0,0938	3	3
	Er+2XmAbs vs. Osimertinib	ns	0,6458	3	3
	Er+2XmAbs vs. Os+2XmAbs	ns	0,8686	3	3
	Er+2XmAbs vs. Afatinib	ns	>0,9999	3	3
	Er+2XmAbs vs. Af+2XmAbs	ns	0,762	3	3
	Osimertinib vs. Os+2XmAbs	ns	0,9998	3	3
	Osimertinib vs. Afatinib	ns	0,7095	3	3
	Osimertinib vs. Af+2XmAbs	ns	0,063	3	3
	Os+2XmAbs vs. Afatinib	ns	0,9093	3	3
	Os+2XmAbs vs. Af+2XmAbs	ns	0,1349	3	3
	Afatinib vs. Af+2XmAbs	ns	0,7011	3	3
**Appendix S1D**	Control vs. 2XmAbs	ns	0,9679	3	3
	Control vs. Erlotinib	ns	>0,9999	3	3
	Control vs. Er+2XmAbs	ns	0,5811	3	3
	Control vs. Osimertinib	ns	0,1355	3	3
	Control vs. Os+2XmAbs	**	0,0028	3	3
	Control vs. Afatinib	ns	0,5206	3	3
	Control vs. Af+2XmAbs	*	0,0267	3	3
	2XmAbs vs. Erlotinib	ns	0,9847	3	3
	2XmAbs vs. Er+2XmAbs	ns	0,9838	3	3
	2XmAbs vs. Osimertinib	ns	0,5637	3	3
	2XmAbs vs. Os+2XmAbs	*	0,0194	3	3
	2XmAbs vs. Afatinib	ns	0,9705	3	3
	2XmAbs vs. Af+2XmAbs	ns	0,1646	3	3
	Erlotinib vs. Er+2XmAbs	ns	0,6566	3	3
	Erlotinib vs. Osimertinib	ns	0,1679	3	3
	Erlotinib vs. Os+2XmAbs	**	0,0036	3	3
	Erlotinib vs. Afatinib	ns	0,5957	3	3
	Erlotinib vs. Af+2XmAbs	*	0,0341	3	3
	Er+2XmAbs vs. Osimertinib	ns	0,9627	3	3
	Er+2XmAbs vs. Os+2XmAbs	ns	0,101	3	3
	Er+2XmAbs vs. Afatinib	ns	>0,9999	3	3
	Er+2XmAbs vs. Af+2XmAbs	ns	0,562	3	3
	Osimertinib vs. Os+2XmAbs	ns	0,4828	3	3
	Osimertinib vs. Afatinib	ns	0,9787	3	3
	Osimertinib vs. Af+2XmAbs	ns	0,9835	3	3
	Os+2XmAbs vs. Afatinib	ns	0,121	3	3

	Os+2XmAbs vs. Af+2XmAbs	ns	0,9309	3	3
	Afatinib vs. Af+2XmAbs	ns	0,623	3	3
**Appendix S2C**	Control vs. 2XmAbs	ns	>0,9999	3	3
	Control vs. Erl	ns	0,9515	3	3
	Control vs. Erl+2XmAbs	ns	0,4922	3	3
	Control vs. Osim	ns	0,2998	3	3
	Control vs. Osim+2XmAbs	ns	0,329	3	3
	Control vs. Afat	ns	0,3266	3	3
	Control vs. Afat+2XmAbs	ns	0,3261	3	3
**Appendix S2D**	Control vs. 2XmAbs	**	0,0055	3	3
	Control vs. Erl	****	<0,0001	3	3
	Control vs. Erl+2XmAbs	****	<0,0001	3	3
	Control vs. Osim	****	<0,0001	3	3
	Control vs. Osim+2XmAbs	****	<0,0001	3	3
	Control vs. Afat	****	<0,0001	3	3
	Control vs. Afat+2XmAbs	****	<0,0001	3	3
**Appendix S2E**	Control vs. 2XmAbs	ns	0,9999	3	3
	Control vs. Erl	ns	0,71	3	3
	Control vs. Erl+2XmAbs	ns	0,2913	3	3
	Control vs. Osim	ns	0,083	3	3
	Control vs. Osim+2XmAbs	ns	0,1711	3	3
	Control vs. Afat	ns	0,1102	3	3
	Control vs. Afat+2XmAbs	ns	0,1165	3	3
**Appendix S3C**	Control vs. 2XmAbs	ns	0,9953	5	7
	Control vs. Erlotinib	ns	>0,9999	5	7
	Control vs. Er+2XmAbs	**	0,0081	5	9
	Control vs. Osimertinib	ns	>0,9999	5	7
	Control vs. Os+2XmAbs	ns	0,9985	5	9
	Control vs. Afatinib	ns	>0,9999	5	8
	Control vs. Af+2XmAbs	ns	0,9035	5	9
	2XmAbs vs. Erlotinib	ns	0,9978	7	7
	2XmAbs vs. Er+2XmAbs	*	0,0293	7	9
	2XmAbs vs. Osimertinib	ns	0,9988	7	7
	2XmAbs vs. Os+2XmAbs	ns	>0,9999	7	9
	2XmAbs vs. Afatinib	ns	0,9734	7	8
	2XmAbs vs. Af+2XmAbs	ns	0,9992	7	9
	Erlotinib vs. Er+2XmAbs	**	0,0039	7	9
	Erlotinib vs. Osimertinib	ns	>0,9999	7	7
	Erlotinib vs. Os+2XmAbs	ns	0,9995	7	9
	Erlotinib vs. Afatinib	ns	>0,9999	7	8

	Erlotinib vs. Af+2XmAbs	ns	0,9126	7	9
	Er+2XmAbs vs. Osimertinib	**	0,0047	9	7
	Er+2XmAbs vs. Os+2XmAbs	**	0,009	9	9
	Er+2XmAbs vs. Afatinib	***	0,0008	9	8
	Er+2XmAbs vs. Af+2XmAbs	ns	0,0739	9	9
	Osimertinib vs. Os+2XmAbs	ns	0,9998	7	9
	Osimertinib vs. Afatinib	ns	>0,9999	7	8
	Osimertinib vs. Af+2XmAbs	ns	0,9328	7	9
	Os+2XmAbs vs. Afatinib	ns	0,9869	9	8
	Os+2XmAbs vs. Af+2XmAbs	ns	0,9935	9	9
	Afatinib vs. Af+2XmAbs	ns	0,7282	8	9
