# Supplementary material for: Upfront admixing antibodies and EGFR inhibitors preempts sequential treatments in lung cancer models
Source: EMBO Mol Med. 2021 Mar 4;13(4):e13144. doi: 10.15252/emmm.202013144 (PMC8033519; doi:10.15252/emmm.202013144)
Supplement: Supplementary file 2 — Expanded View Figures PDF [file EMMM-13-e13144-s004.pdf]

Expanded View Figures

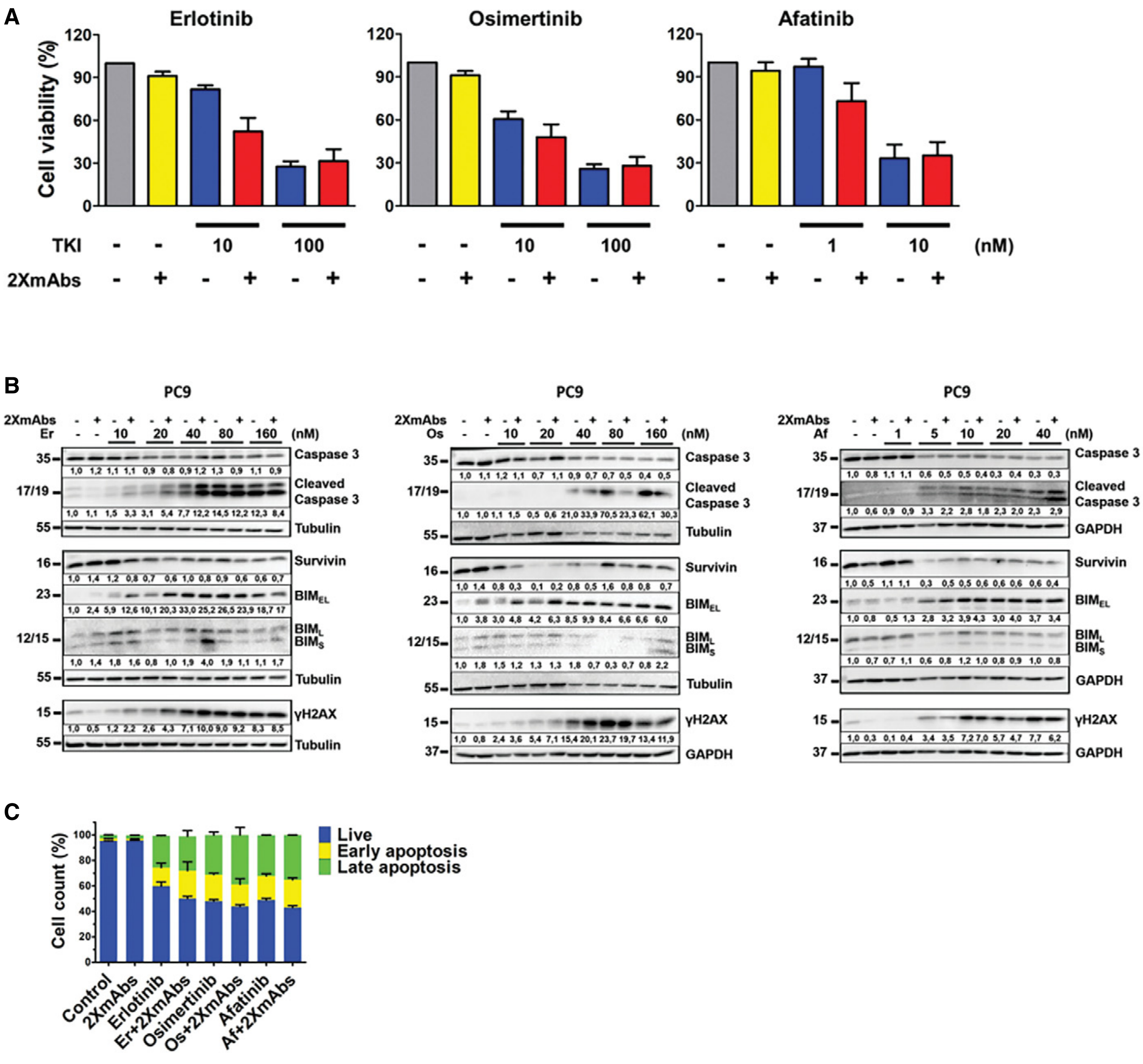

Figure EV1. The addition of antibodies reduces viability and enhances TKI-induced apoptosis.

A PC9 cells (5×10^3) were seeded in 96-well plates and later treated for 72 h with two different concentrations of EGFR-specific TKIs (erlotinib or osimertinib, at 10 or 100 nM; afatinib, at 1 or 10 nM), either alone or combined with 2XmAbs (cetuximab and trastuzumab, each at 5 μg/ml). Cell viability was assessed using the MTT (3-(4,5-dimethylthiazol-2-yl)-2,5-diphenyltetrazolium bromide) assay. Results are presented as mean + SEM of two independent experiments.

B PC9 cells were treated for 48 h with increasing concentrations of EGFR-specific TKIs (erlotinib or osimertinib at 10, 20, 40, 80, and 160 nM; afatinib at 1, 5, 10, 20 and 40 nM), either alone or in combination with 2XmAbs (cetuximab plus trastuzumab, each at 5 μg/ml). Protein extracts were resolved, blotted, and probed with antibodies specific to the indicated apoptosis markers. Tubulin (or GAPDH) was used as loading control. Signals (relative to control) were quantified and normalized to the signals of GAPDH or tubulin (numbers shown below each lane).

C PC9 cells were treated for 48 h with erlotinib (40 nM), osimertinib (40 nM), afatinib (10 nM), 2XmAbs (cetuximab and trastuzumab, each at 5 μg/ml), or combinations of mAbs and TKIs. Apoptosis was assayed using cytometry and the Annexin V/7-AAD kit (from BioLegend, Inc). The histogram shows the means + SEM of two experiments.

Source data are available online for this figure.

© 2021 The Authors

Figure EV2. Combinations of 2XmAbs and EGFR-specific TKIs reduce viability and enhance apoptosis of cells expressing L858R-EGFR.

A H3255 cells (3×10^4) were seeded in 96-well plates and treated for 72 h with increasing concentrations of TKIs (erlotinib or osimertinib at 1, 5, 10, 50, and 100 nM; afatinib at 0.1, 0.5, 1, 2, and 10 nM), either alone or in combination with 2XmAbs (cetuximab and trastuzumab, each at 5 μg/ml). Cell viability was assessed using the MTT assay. Results are shown as mean of four experiments + standard error.

B H3255 and PC9 cells were treated for 60 min with 2XmAbs (cetuximab and trastuzumab, each at 5 μg/ml), either alone or in combination with the following TKIs: erlotinib or osimertinib (each at 50 nM) or afatinib (10 nM). Protein extracts were immunoblotted using the indicated antibodies. Signals (relative to control) were quantified and normalized to the signals of GAPDH (numbers shown below each lane).

C H3255 cells were treated for 48 h with increasing concentrations of EGFR-specific TKIs (erlotinib or osimertinib at 5, 10, 20, 40, and 80 nM, or afatinib at 0.1, 0.5, 1, 2, and 10 nM), either alone or in combination with 2XmAbs (cetuximab and trastuzumab, each at 5 μg/ml). Protein extracts were resolved using electrophoresis, transferred to nitrocellulose membranes, and blotted with the indicated antibodies. GAPDH was used as a loading control. Signals (relative to control) were quantified and normalized to the signals of GAPDH (numbers shown below each lane).

Source data are available online for this figure.

 © 2021 The Authors

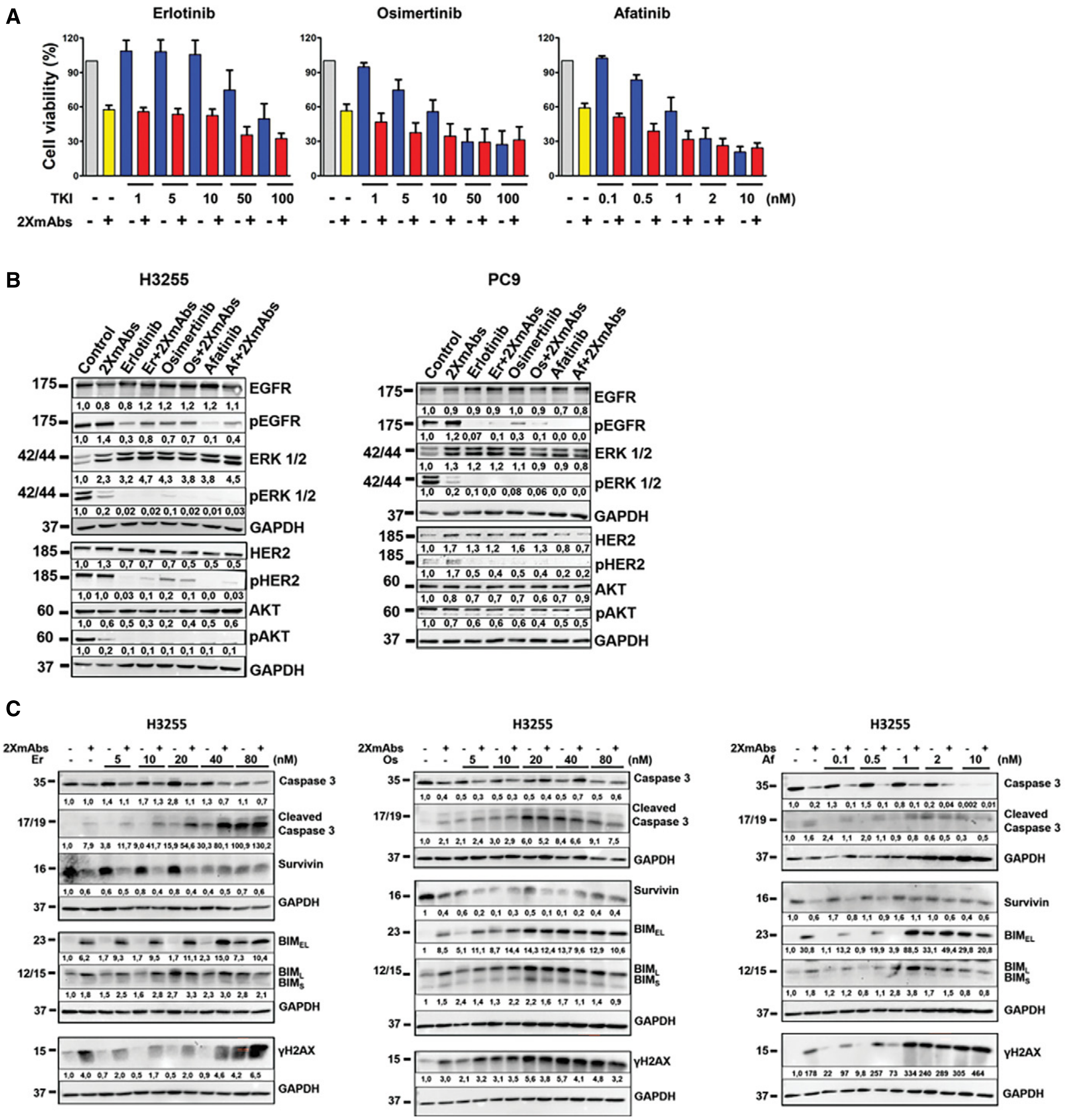

Figure EV2.

Figure EV3. Short-term treatments of xenografts with combinations of monoclonal antibodies and either erlotinib or osimertinib achieve similar anti-tumor efficacies.

A, B PC9 cells (exon 19 deletion) were subcutaneously implanted in the flanks of CD1-nu/nu mice (3×10^6/mouse). When tumors became palpable, mice were randomized into groups of 4–10 animals, which were treated for 30 days with 2XmAbs (cetuximab and trastuzumab, each at 0.1 mg/mouse/injection) once every three days, or daily with the indicated TKIs, osimertinib (10 mg/kg) or erlotinib (50 mg/kg). A third group was treated with the respective combinations of a TKI and the two mAbs. Tumor growth (A) and animal survival (B) are shown. Mice were euthanized when tumor size reached 1,500 mm3. Data are means ± SEM from 4–10 mice per group. Note that the gray areas mark time windows of animal treatment.

C Statistical analysis of tumor volumes corresponding to the last measurement for each mouse was performed using one-way ANOVA followed by Tukey's multiple comparison test. Results are shown as mean ± SEM. See *P*-values and number of mice per group (N) in Appendix Table S2. Note that only the significant comparisons are shown.

D Shown are tumor volumes corresponding to individual mice. The days of treatment are indicated by the gray area.

© 2021 The Authors

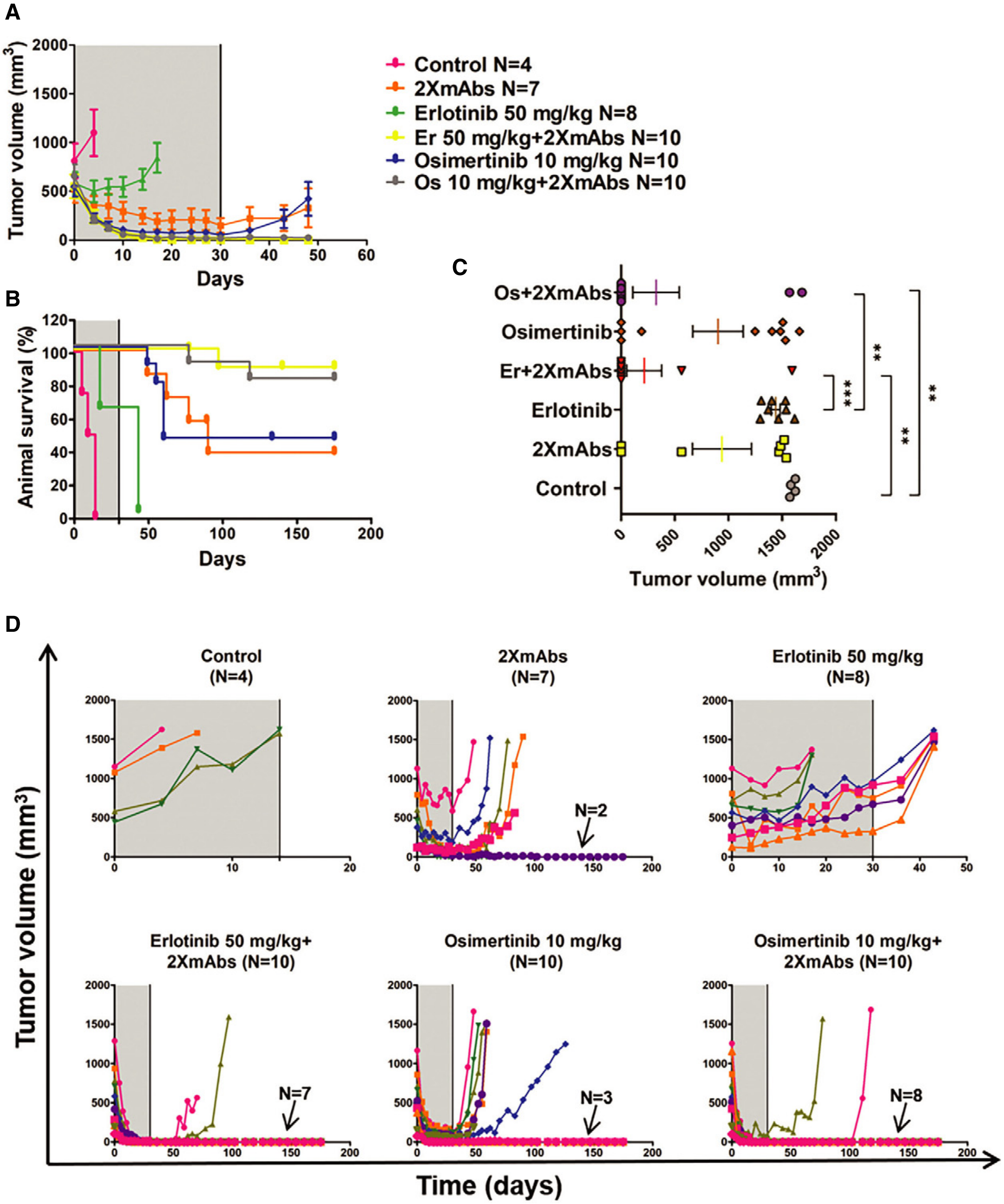
A
Tumor volume (mm3)
Days
Control N=4
2XmAbs N=7
Erlotinib 50 mg/kg N=8
Er 50 mg/kg+2XmAbs N=10
Osimertinib 10 mg/kg N=10
Os 10 mg/kg+2XmAbs N=10
B
Animal survival (%)
Days
C
Os+2XmAbs
Osimertinib
Er+2XmAbs
Erlotinib
2XmAbs
Control
Tumor volume (mm3)
D
Control (N=4)
2XmAbs (N=7)
N=2
Erlotinib 50 mg/kg (N=8)
Erlotinib 50 mg/kg+ 2XmAbs (N=10)
N=7
Osimertinib 10 mg/kg (N=10)
N=3
Osimertinib 10 mg/kg+ 2XmAbs (N=10)
N=8
Tumor volume (mm3)
Time (days)

Figure EV3.

Figure EV4. When tested in vivo, the BCR-ABL-specific inhibitor, imatinib, only weakly affects the abundance of survivin and RTKs, or levels of activation of downstream pathways.

A Nude mice bearing PC9 xenografts (3 per group) were treated for one week with 2XmAbs (each at 0.1 mg/injection/mouse), imatinib (100 mg/kg/day), or the respective drug combination. The fold change in tumor volume after 7 days of treatment is shown. The horizontal line indicates no change in tumor volume (fold change) between day 0 and day 7 of treatment.

B Immunoblots of extracts prepared from the animals and tumors presented in A are shown. GAPDH and tubulin were used as measures of total protein loaded. Note that mice in the control and 2XmAbs groups are presented in Fig 4. Signals were quantified and normalized to the signals of GAPDH or tubulin (numbers shown below each lane).

C PC9 cells were treated for 4 h with increasing concentrations of different TKIs (erlotinib or osimertinib, at 12.5, 25, and 50 nM; afatinib, at 2.5, 5, and 10 nM; imatinib, at 12.5, 25, and 50 μM). Protein extracts were resolved and immunoblotted for the indicated proteins. Signals (relative to Control) were quantified and normalized to the signals of GAPDH (numbers shown below each lane).

Source data are available online for this figure.

© 2021 The Authors

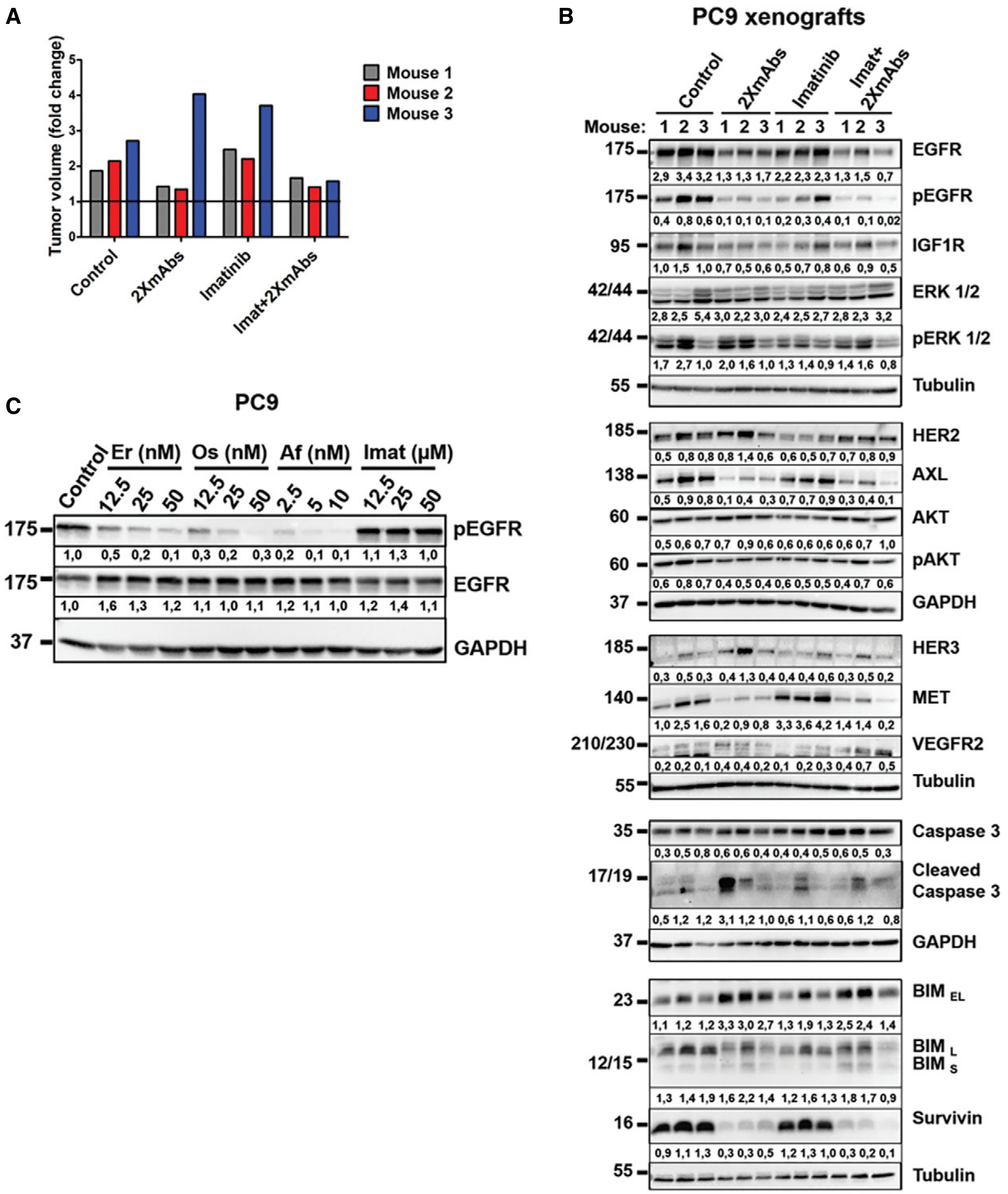
A
Tumor volume (fold change)
Mouse 1
Mouse 2
Mouse 3
Control
2XmAbs
Imatinib
Imat+2XmAbs
B
PC9 xenografts
Control
2XmAbs
Imatinib
Imat+ 2XmAbs
Mouse: 1 2 3 1 2 3 1 2 3 1 2 3
EGFR
pEGFR
IGF1R
ERK 1/2
pERK 1/2
Tubulin
HER2
AXL
AKT
pAKT
GAPDH
HER3
MET
VEGFR2
Tubulin
Caspase 3
Cleaved Caspase 3
GAPDH
BIM EL
BIM L
BIM S
Survivin
Tubulin
C
PC9
Control
Er (nM)
Os (nM)
Af (nM)
Imat (μM)
pEGFR
EGFR
GAPDH

Figure EV4.

Figure EV5. Combining two antibodies and erlotinib overcomes resistance to the TKI by modulating the FOXM1 transcription factor network.

PC9 xenografts were established and treated as described in Fig 7B.

A Tumor volumes are presented as fold change relative to the size of each original tumor (untreated; day 0). The horizontal line indicates no change in tumor volume (fold change) between day 0 and the day the mice were sacrificed.

B RNA was extracted from the tumors shown in (A) and subjected to RNA-seq analysis. Differentially expressed (DE) genes in the erlotinib (Er) + 2XmAbs compared with the control group are presented in the BioPlanet pathway enrichment analysis (over-representation). The x-axis shows the negative logarithm (-Log *P*-value) of the enrichment adjusted *P*-value. Pathways relevant to the cell cycle are shown in bold.

C RNA extracted from the tumors shown in (A) was analyzed using qPCR and primers specific to the indicated transcripts. Averages ± SD of 2–5 mice are shown. GAPDH was used for normalization.

D PC9 cells were seeded on coverslips and treated for 24 h with 2XmAbs (cetuximab + trastuzumab, 10 μg/ml), erlotinib (20 nM), or a combination of all three drugs. Cells were fixed in paraformaldehyde (4%) and incubated with a FOXO3a primary antibody, followed by an Alexa Fluor 555-conjugated secondary antibody (red). DAPI (blue) was used to stain nuclei. FITC-conjugated phalloidin was used to stain actin filaments (green). Images were captured using a confocal microscope (40× magnification). Scale bar, 20 μm.

E PC9 cells were treated for 24 or 48 h as described in (D). Protein extracts were immunoblotted with the indicated antibodies. GAPDH was used to control the amount of loaded protein. Signals (relative to Control) were quantified and normalized to the signals of GAPDH (numbers shown below each lane).

Source data are available online for this figure.

 © 2021 The Authors

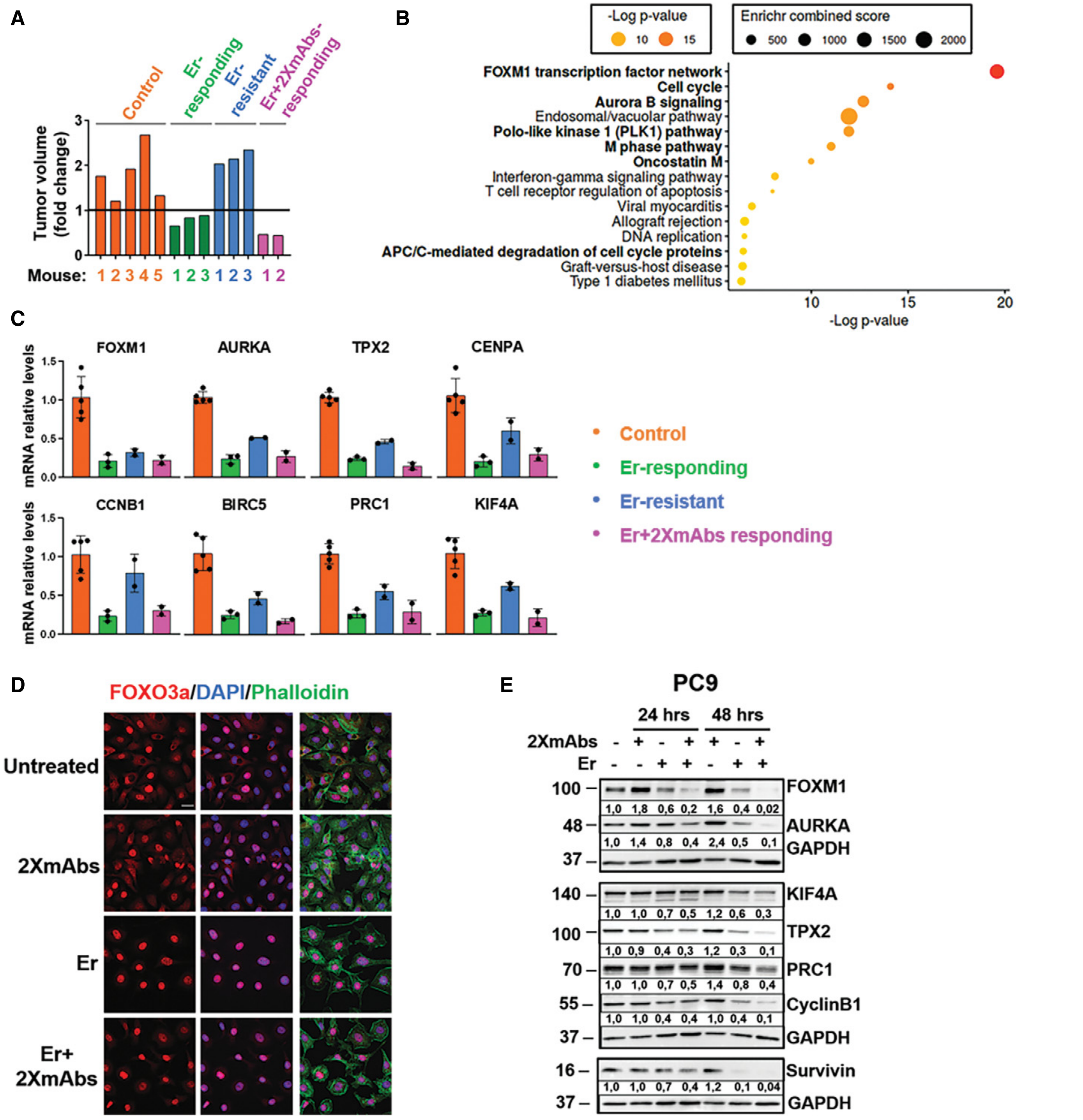
A
Control
Er-responding
Er-resistant
Er+2XmAbs-responding
Tumor volume (fold change)
0
1
2
3
Mouse: 1 2 3 4 5 1 2 3 1 2 3 1 2
B
-Log p-value
10
15
Enrichr combined score
500
1000
1500
2000
FOXM1 transcription factor network
Cell cycle
Aurora B signaling
Endosomal/vacuolar pathway
Polo-like kinase 1 (PLK1) pathway
M phase pathway
Oncostatin M
Interferon-gamma signaling pathway
T cell receptor regulation of apoptosis
Viral myocarditis
Allograft rejection
DNA replication
APC/C-mediated degradation of cell cycle proteins
Graft-versus-host disease
Type 1 diabetes mellitus
10
15
20
-Log p-value
C
FOXM1
AURKA
TPX2
CENPA
CCNB1
BIRC5
PRC1
KIF4A
mRNA relative levels
0.0
0.5
1.0
1.5
Control
Er-responding
Er-resistant
Er+2XmAbs responding
D
FOXO3a/DAPI/Phalloidin
Untreated
2XmAbs
Er
Er+ 2XmAbs
E
PC9
24 hrs
48 hrs
2XmAbs - + - + + - +
Er - - + + - + +
100 – FOXM1
1,0 1,8 0,6 0,2 1,6 0,4 0,02
48 – AURKA
1,0 1,4 0,8 0,4 2,4 0,5 0,1
GAPDH
37 –
140 – KIF4A
1,0 1,0 0,7 0,5 1,2 0,6 0,3
100 – TPX2
1,0 0,9 0,4 0,3 1,2 0,3 0,1
70 – PRC1
1,0 1,0 0,7 0,5 1,4 0,8 0,4
55 – CyclinB1
1,0 1,0 0,4 0,4 1,0 0,4 0,1
37 – GAPDH
16 – Survivin
1,0 1,0 0,7 0,4 1,2 0,1 0,04
37 – GAPDH

Figure EV5.
