# Supplementary material for: Upfront admixing antibodies and EGFR inhibitors preempts sequential treatments in lung cancer models
Source: EMBO Mol Med. 2021 Mar 4;13(4):e13144. doi: 10.15252/emmm.202013144 (PMC8033519; doi:10.15252/emmm.202013144)
Supplement: Supplementary file 5 — Source Data for Figure 1 [file EMMM-13-e13144-s001.pdf]

Figure 1A

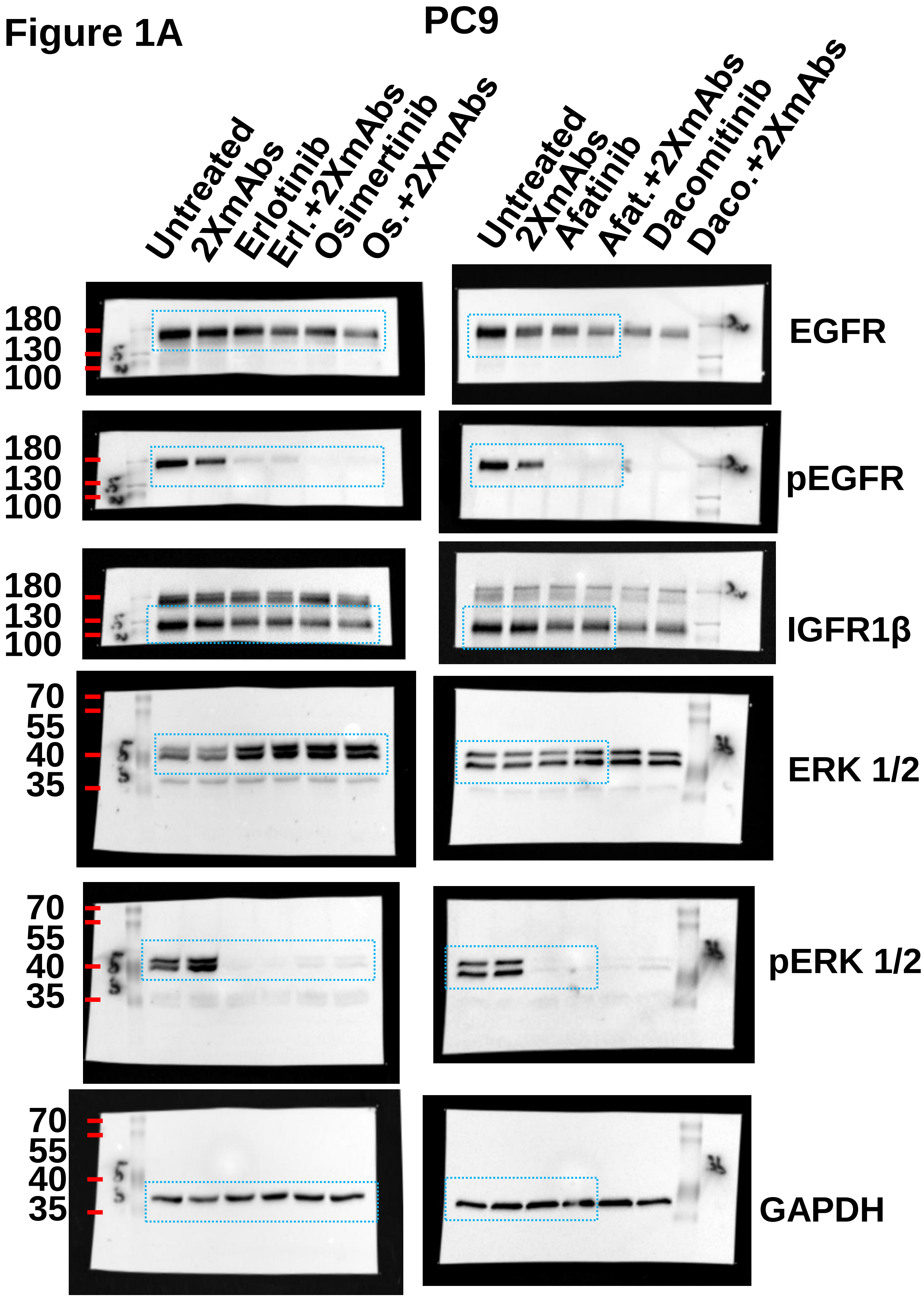
PC9
Untreated
2XmAbs
Erlotinib
Erl.+2XmAbs
Osimertinib
Os.+2XmAbs
Untreated
2XmAbs
Afatinib
Afat.+2XmAbs
Dacomitinib
Daco.+2XmAbs
180
130
100
EGFR
180
130
100
pEGFR
180
130
100
IGFR1β
70
55
40
35
ERK 1/2
70
55
40
35
pERK 1/2
70
55
40
35
GAPDH

Figure 1A

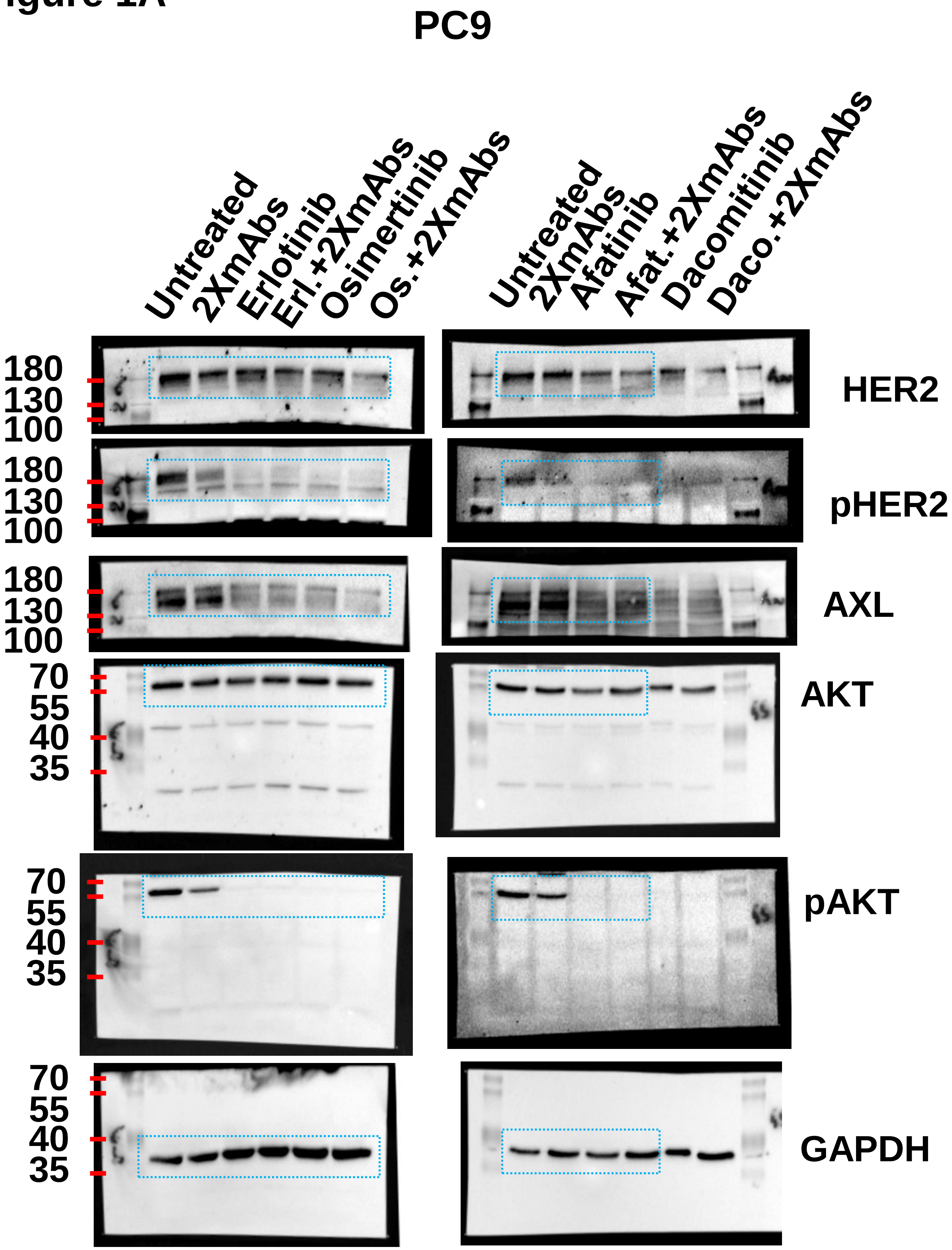
PC9
Untreated
2XmAbs
Erlotinib
Erl.+2XmAbs
Osimertinib
Os.+2XmAbs
Untreated
2XmAbs
Afatinib
Afat.+2XmAbs
Dacomitinib
Daco.+2XmAbs
180
130
100
HER2
180
130
100
pHER2
180
130
100
AXL
70
55
40
35
AKT
70
55
40
35
pAKT
70
55
40
35
GAPDH

Figure 1A

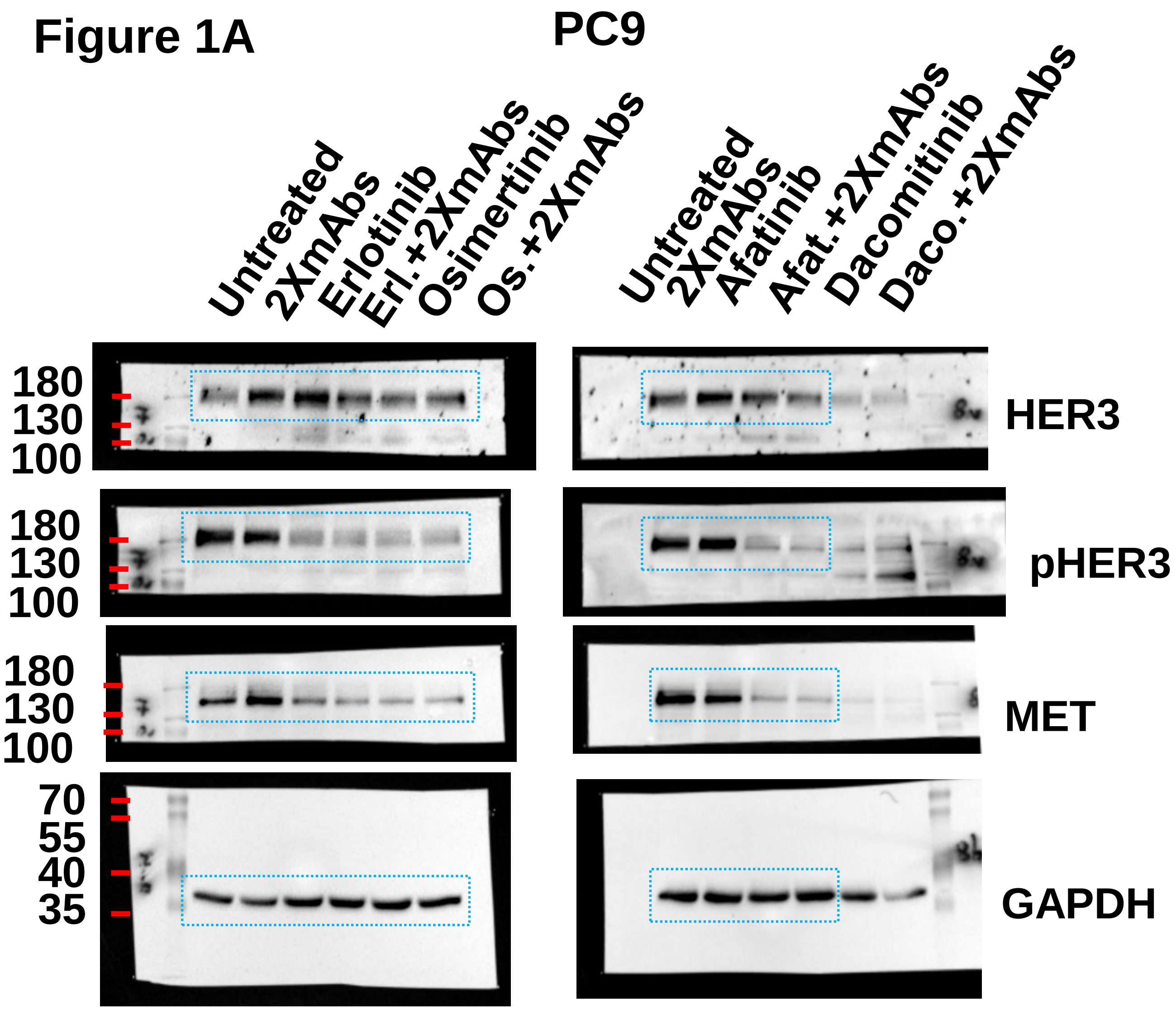
PC9
Untreated
2XmAbs
Erlotinib
Erl.+2XmAbs
Osimertinib
Os.+2XmAbs
Untreated
2XmAbs
Afatinib
Afat.+2XmAbs
Dacomitinib
Daco.+2XmAbs
180
130
100
HER3
180
130
100
pHER3
180
130
100
MET
70
55
40
35
GAPDH

Figure 1A

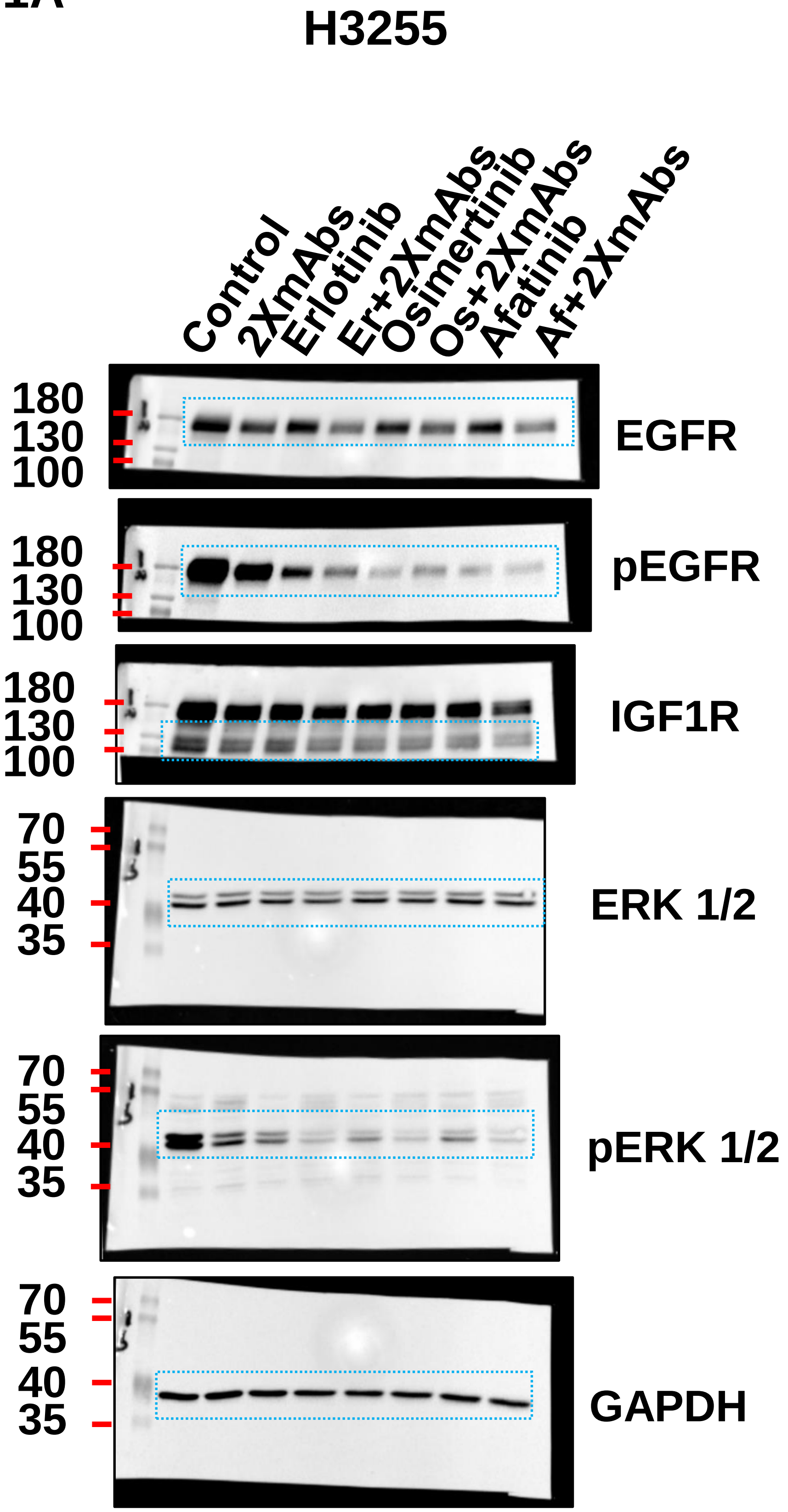
H3255
Control
2XmAbs
Erlotinib
Er+2XmAbs
Osimertinib
Os+2XmAbs
Afatinib
Af+2XmAbs
180
130
100
EGFR
180
130
100
pEGFR
180
130
100
IGF1R
70
55
40
35
ERK 1/2
70
55
40
35
pERK 1/2
70
55
40
35
GAPDH

Figure 1A

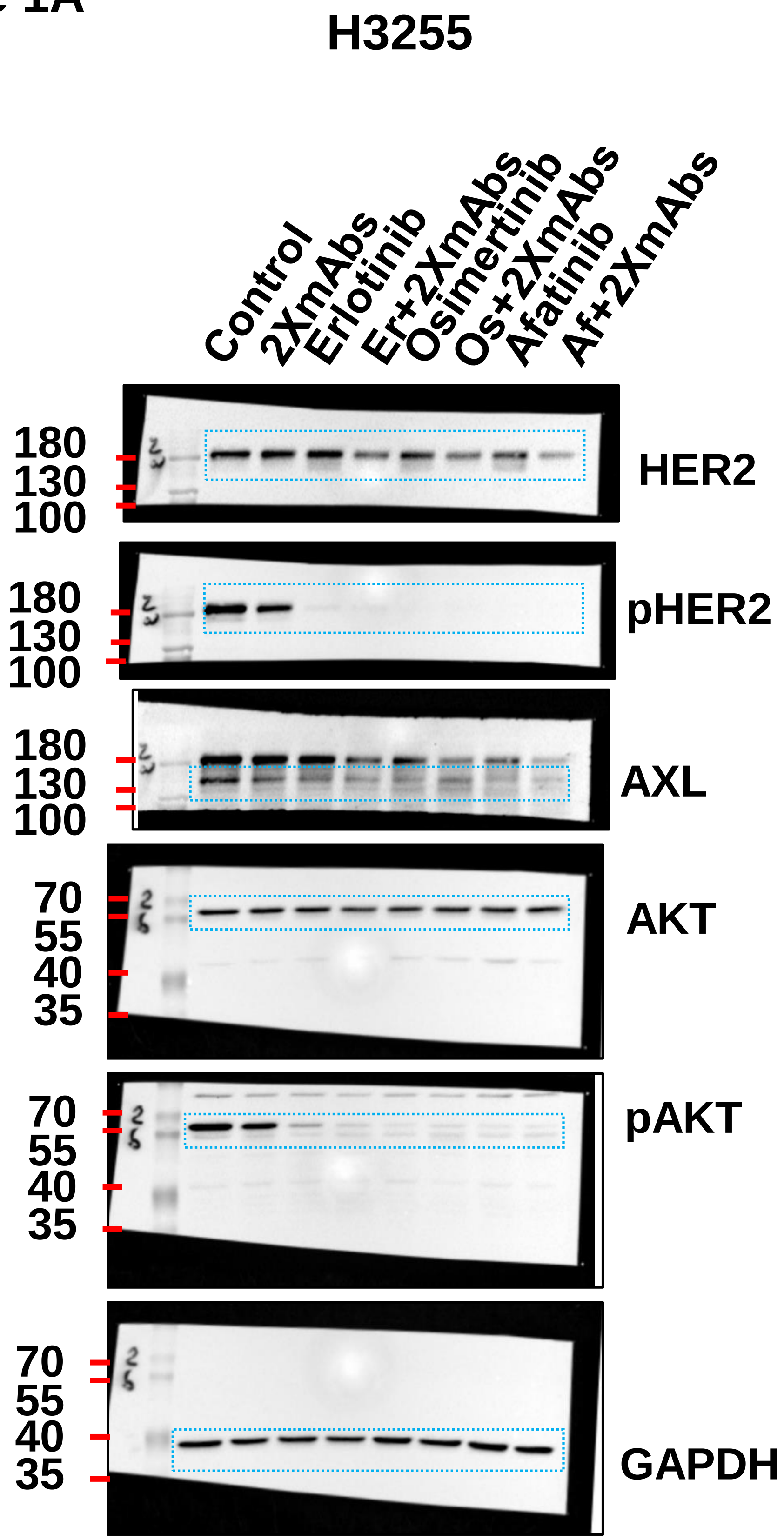
H3255
Control
2XmAbs
Erlotinib
Er+2XmAbs
Osimertinib
Os+2XmAbs
Afatinib
Af+2XmAbs
180
130
100
HER2
180
130
100
pHER2
180
130
100
AXL
70
55
40
35
AKT
70
55
40
35
pAKT
70
55
40
35
GAPDH

Figure 1A

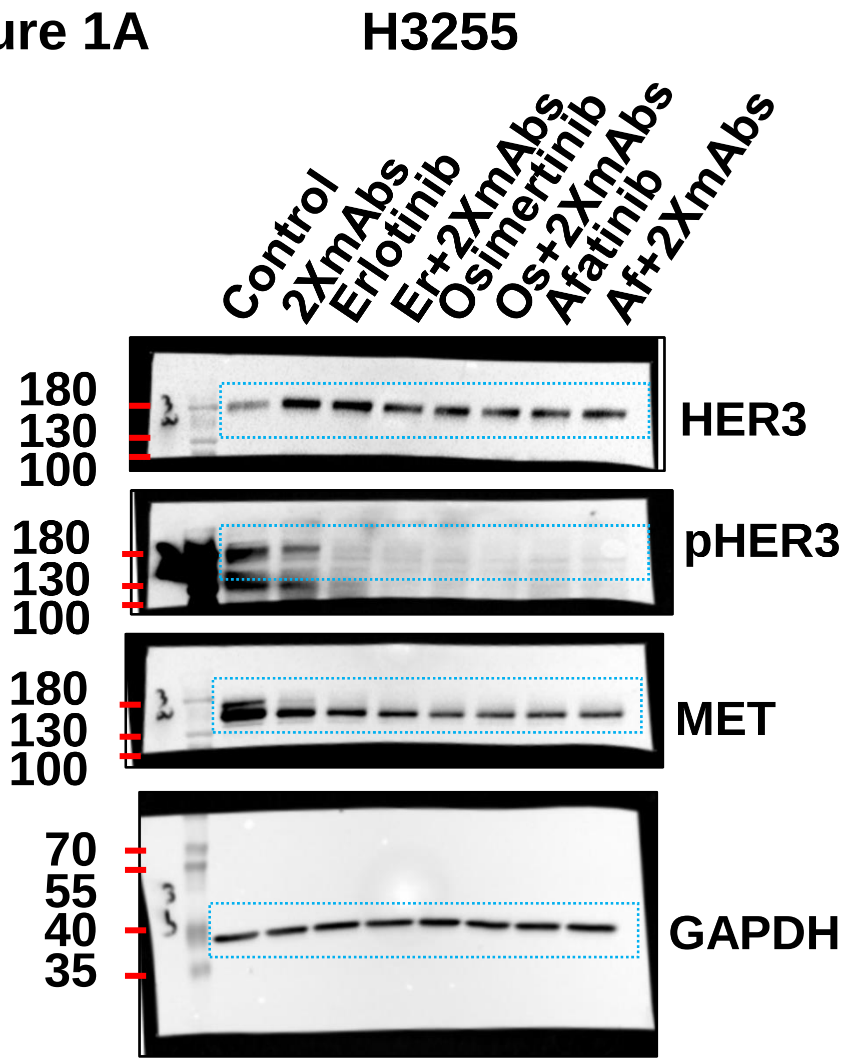
H3255
Control
2XmAbs
Erlotinib
Er+2XmAbs
Osimertinib
Os+2XmAbs
Afatinib
Af+2XmAbs
180
130
100
HER3
180
130
100
pHER3
180
130
100
MET
70
55
40
35
GAPDH

Figure 1C

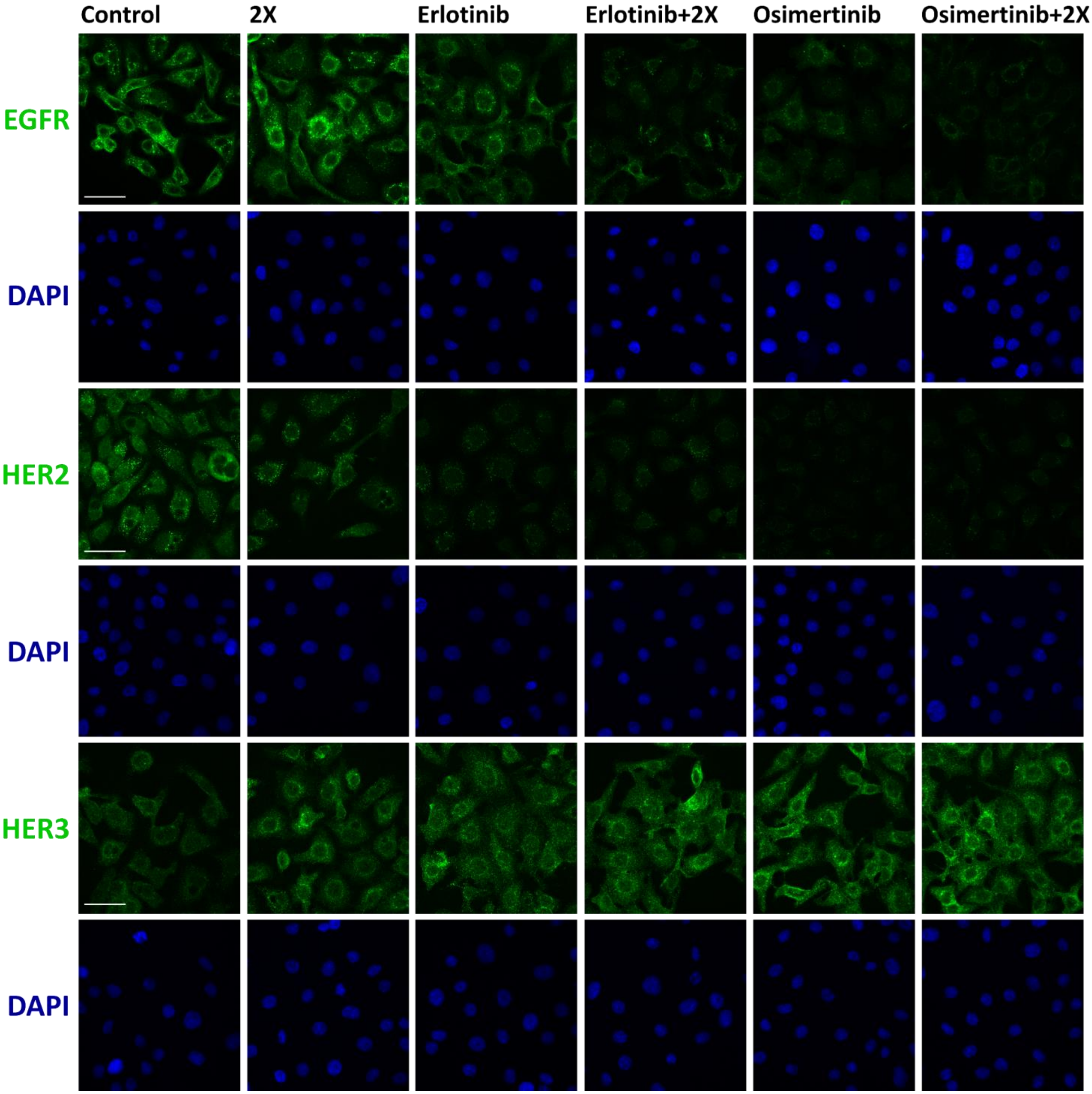

Control
2X
Erlotinib
Erlotinib+2X
Osimertinib
Osimertinib+2X
EGFR
DAPI
HER2
DAPI
HER3
DAPI
