# Supplementary material for: Upfront admixing antibodies and EGFR inhibitors preempts sequential treatments in lung cancer models
Source: EMBO Mol Med. 2021 Mar 4;13(4):e13144. doi: 10.15252/emmm.202013144 (PMC8033519; doi:10.15252/emmm.202013144)
Supplement: Supplementary file 6 — Source Data for Figure 4 [file EMMM-13-e13144-s006.pdf]

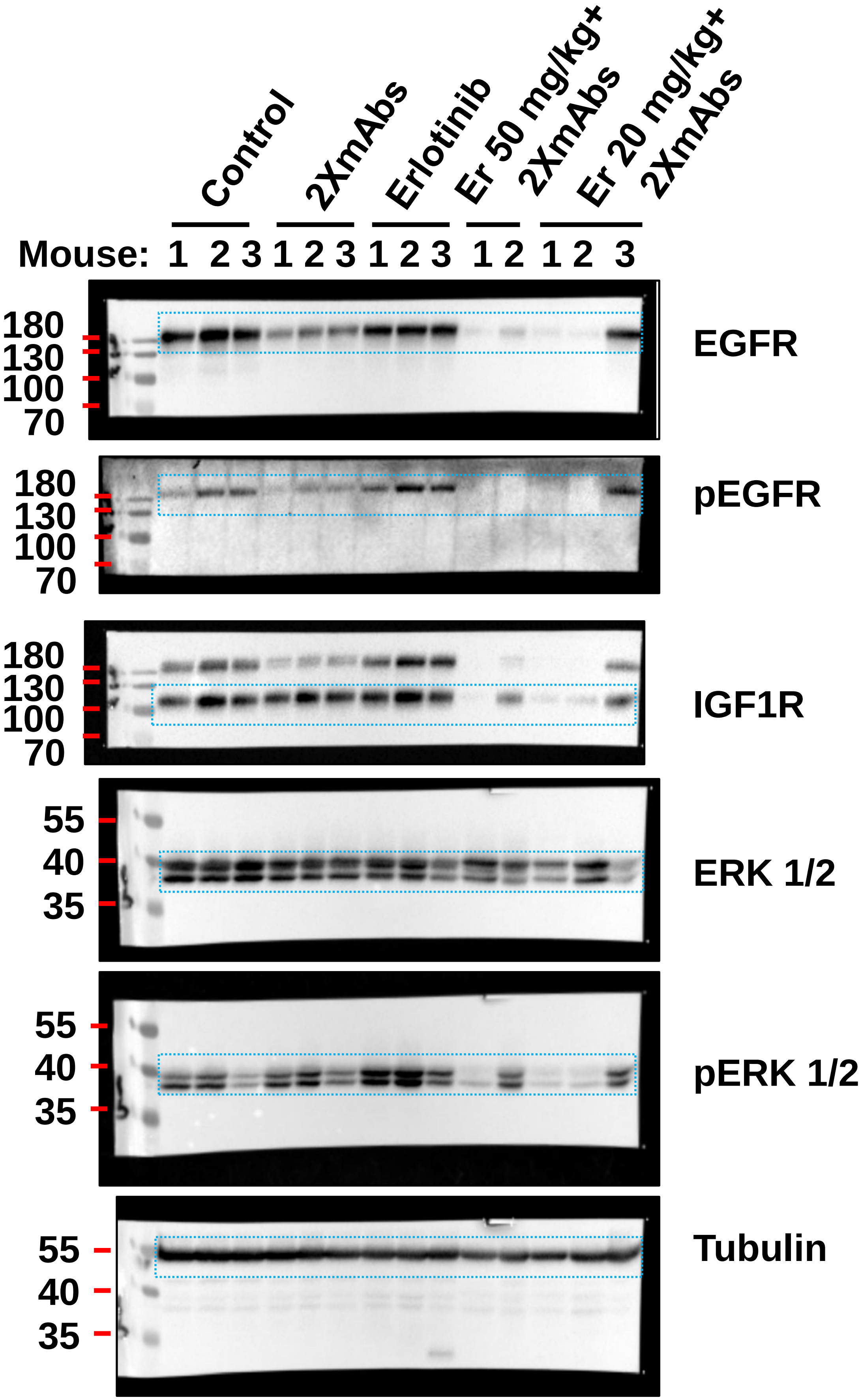

Control
2XmAbs
Erlotinib
Er 50 mg/kg+ 2XmAbs
Er 20 mg/kg+ 2XmAbs
Mouse: 1 2 3 1 2 3 1 2 3 1 2 1 2 3
180
130
100
70
EGFR
pEGFR
IGF1R
55
40
35
ERK 1/2
pERK 1/2
Tubulin

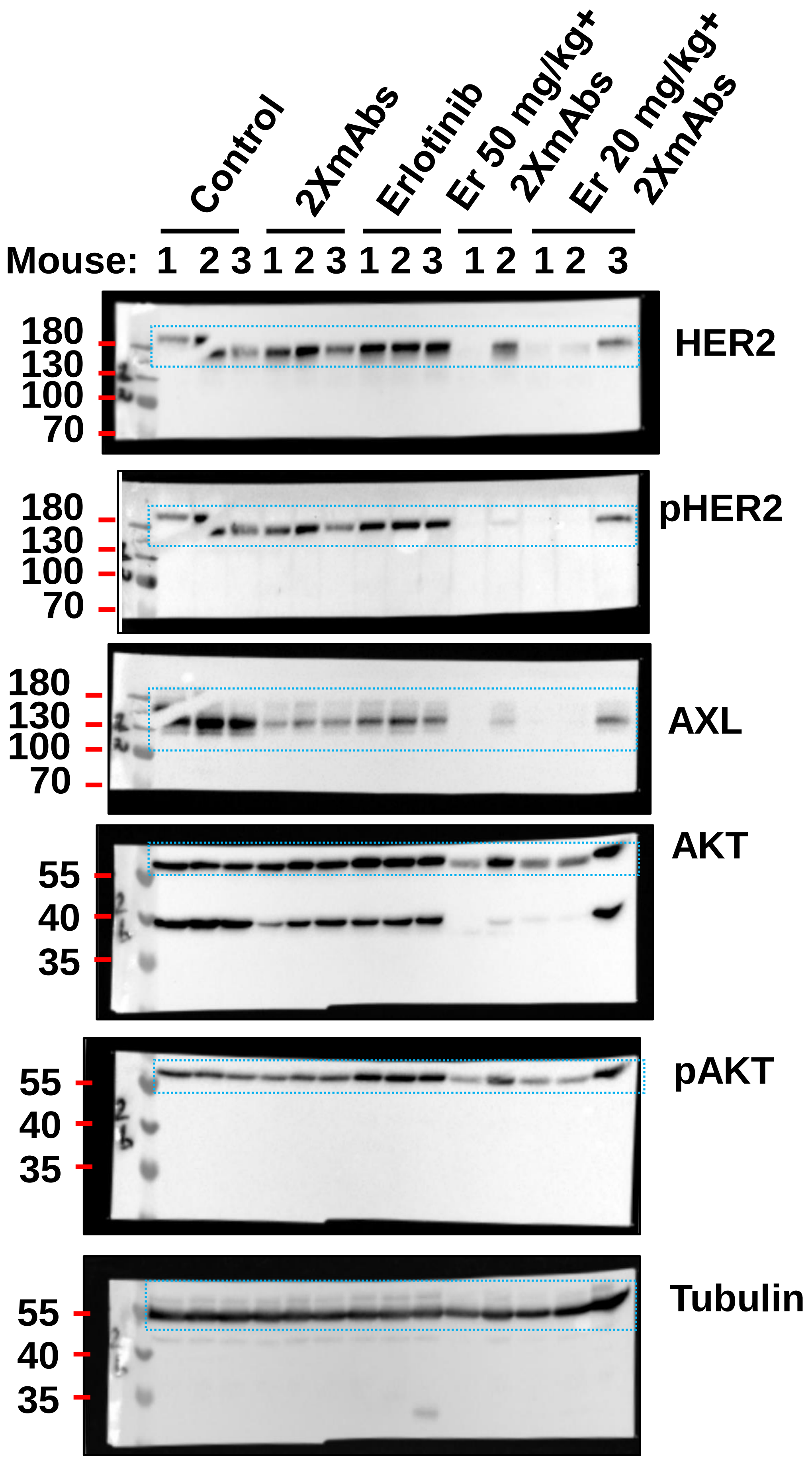

Control
2XmAbs
Erlotinib
Er 50 mg/kg+ 2XmAbs
Er 20 mg/kg+ 2XmAbs
Mouse: 1 2 3 1 2 3 1 2 3 1 2 1 2 3
180
130
100
70
HER2
pHER2
AXL
55
40
35
AKT
pAKT
Tubulin

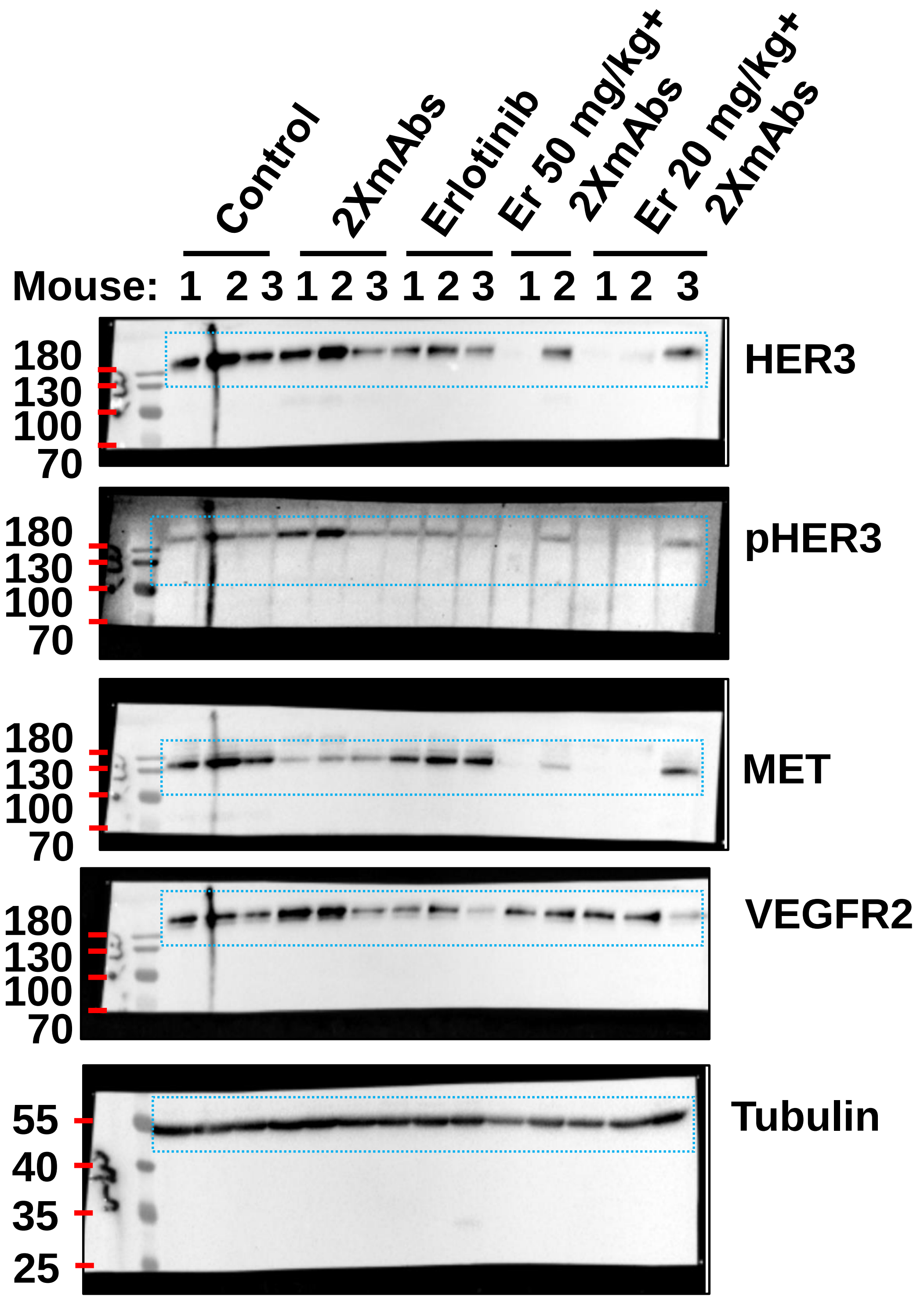

Control
2XmAbs
Erlotinib
Er 50 mg/kg+ 2XmAbs
Er 20 mg/kg+ 2XmAbs
Mouse: 1 2 3 1 2 3 1 2 3 1 2 1 2 3
180
130
100
70
HER3
pHER3
MET
VEGFR2
55
40
35
25
Tubulin

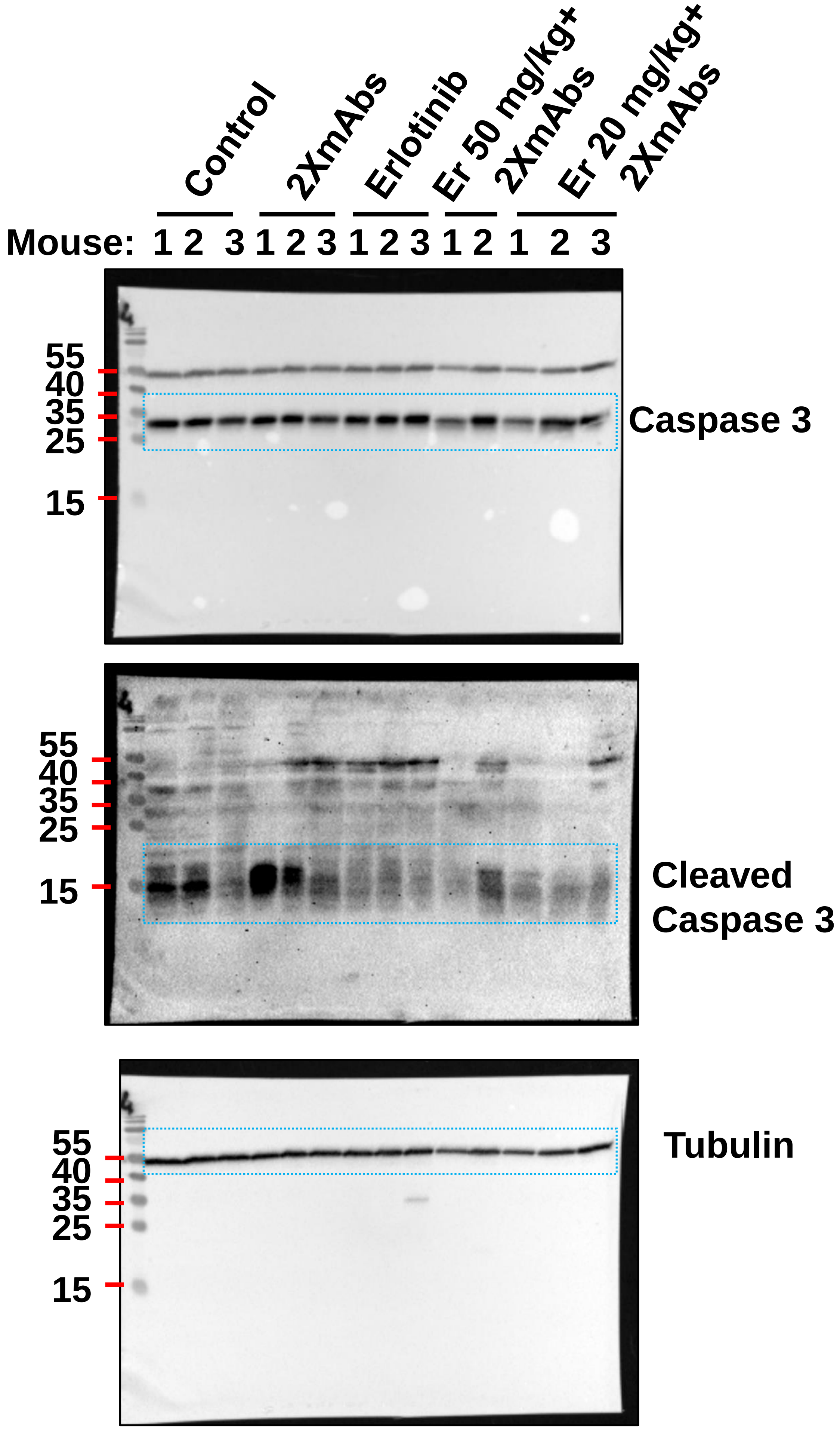
Control
2XmAbs
Erlotinib
Er 50 mg/kg+ 2XmAbs
Er 20 mg/kg+ 2XmAbs
Mouse: 1 2 3 1 2 3 1 2 3 1 2 1 2 3
55
40
35
25
15
Caspase 3
Cleaved Caspase 3
Tubulin

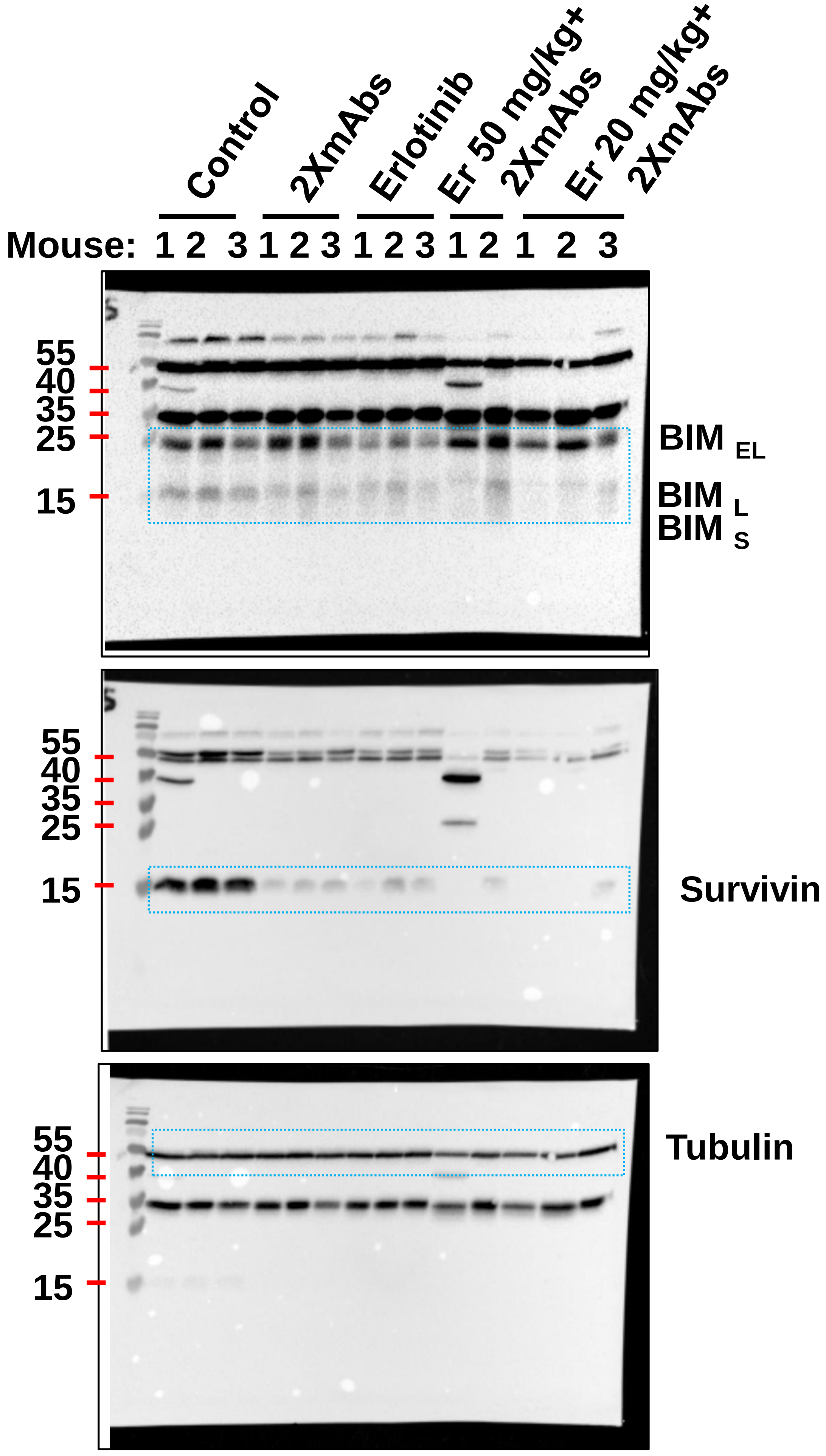

Control
2XmAbs
Erlotinib
Er 50 mg/kg+ 2XmAbs
Er 20 mg/kg+ 2XmAbs
Mouse: 1 2 3 1 2 3 1 2 3 1 2 1 2 3
55
40
35
25
15
BIM EL
BIM L
BIM S
Survivin
Tubulin

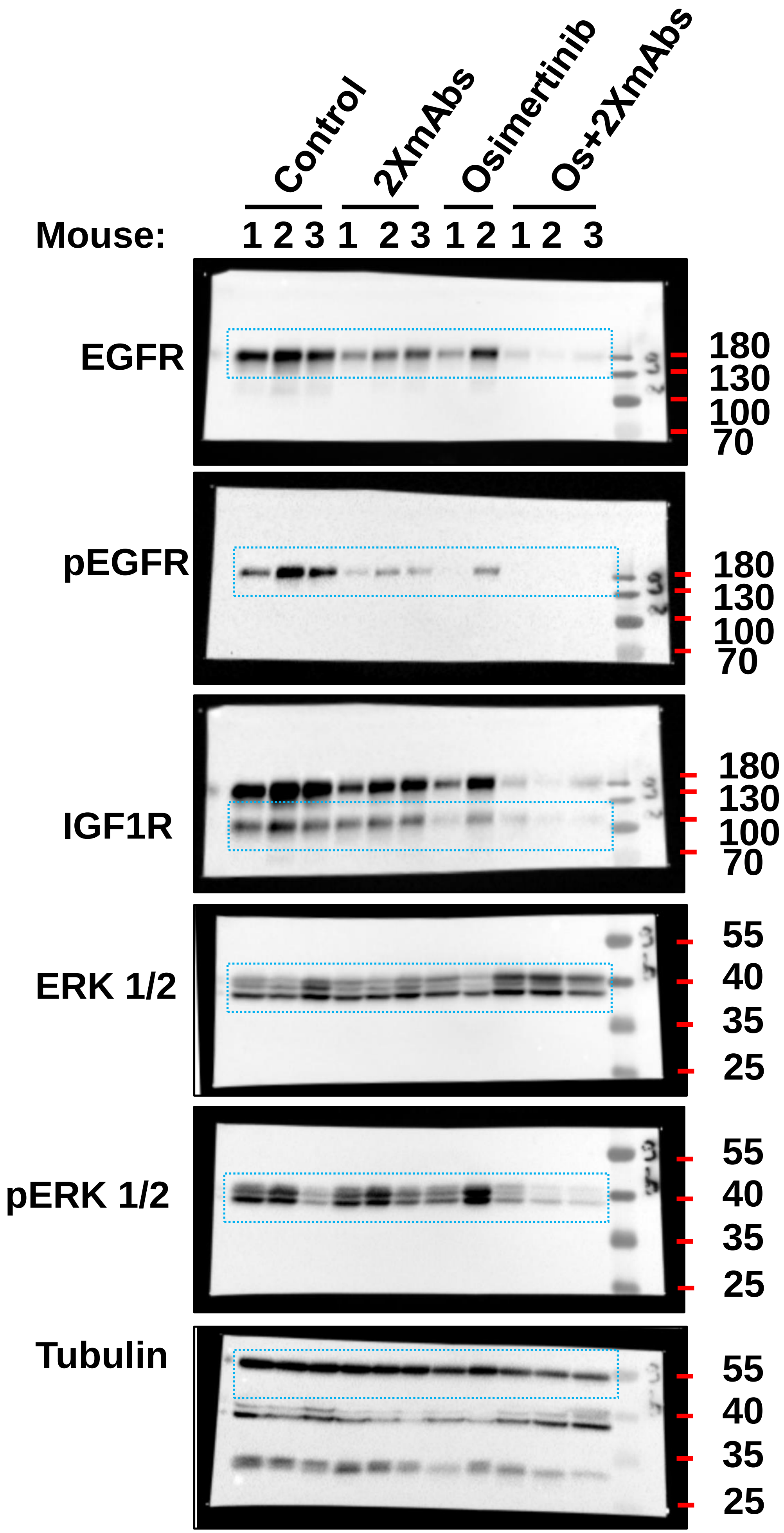

Control
2XmAbs
Osimertinib
Os+2XmAbs
Mouse:
1 2 3 1 2 3 1 2 1 2 3
EGFR
pEGFR
IGF1R
ERK 1/2
pERK 1/2
Tubulin
180
130
100
70
55
40
35
25

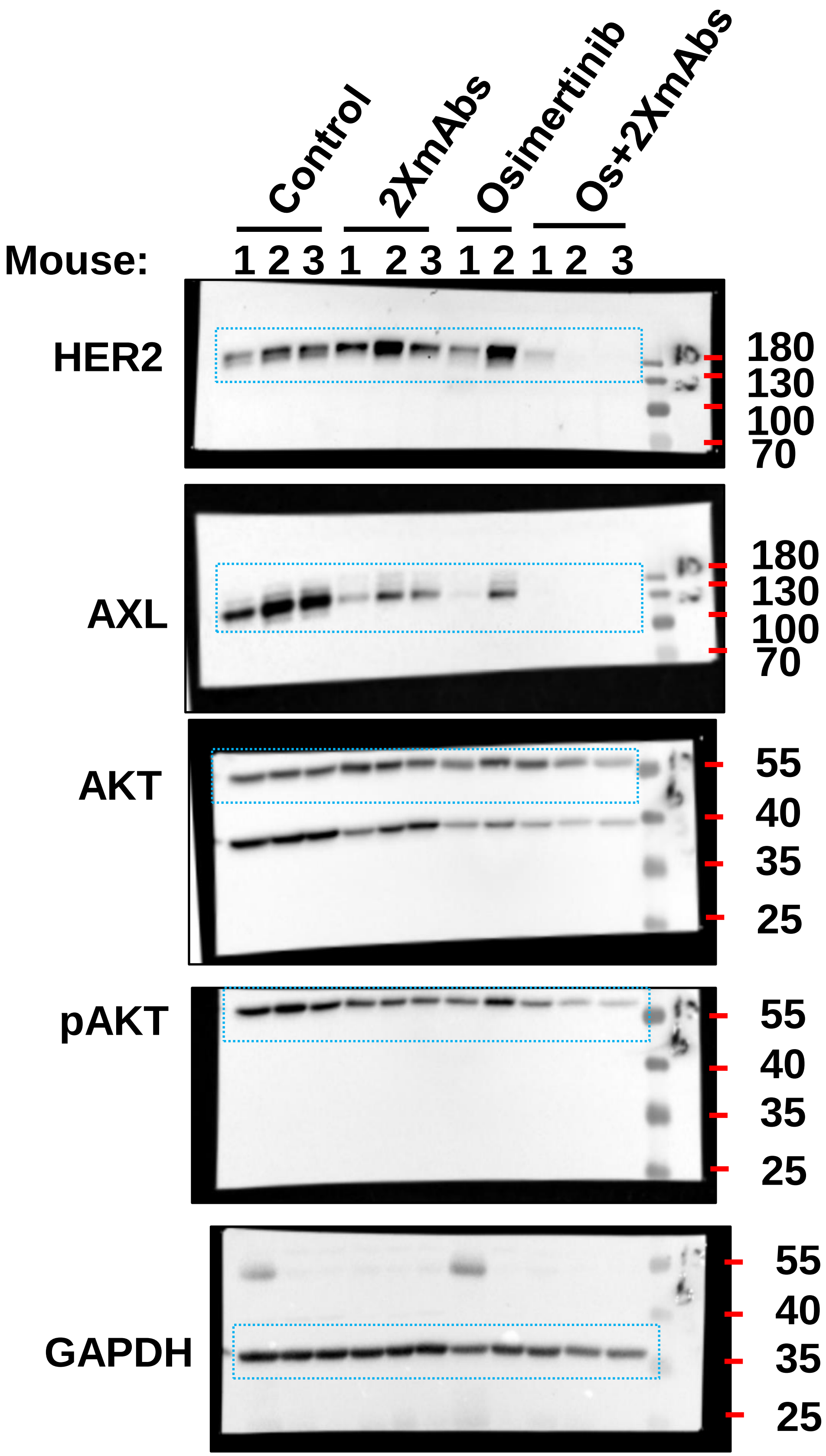

Control
2XmAbs
Osimertinib
Os+2XmAbs
Mouse:
1 2 3 1 2 3 1 2 1 2 3
HER2
180
130
100
70
AXL
180
130
100
70
AKT
55
40
35
25
pAKT
55
40
35
25
GAPDH
55
40
35
25

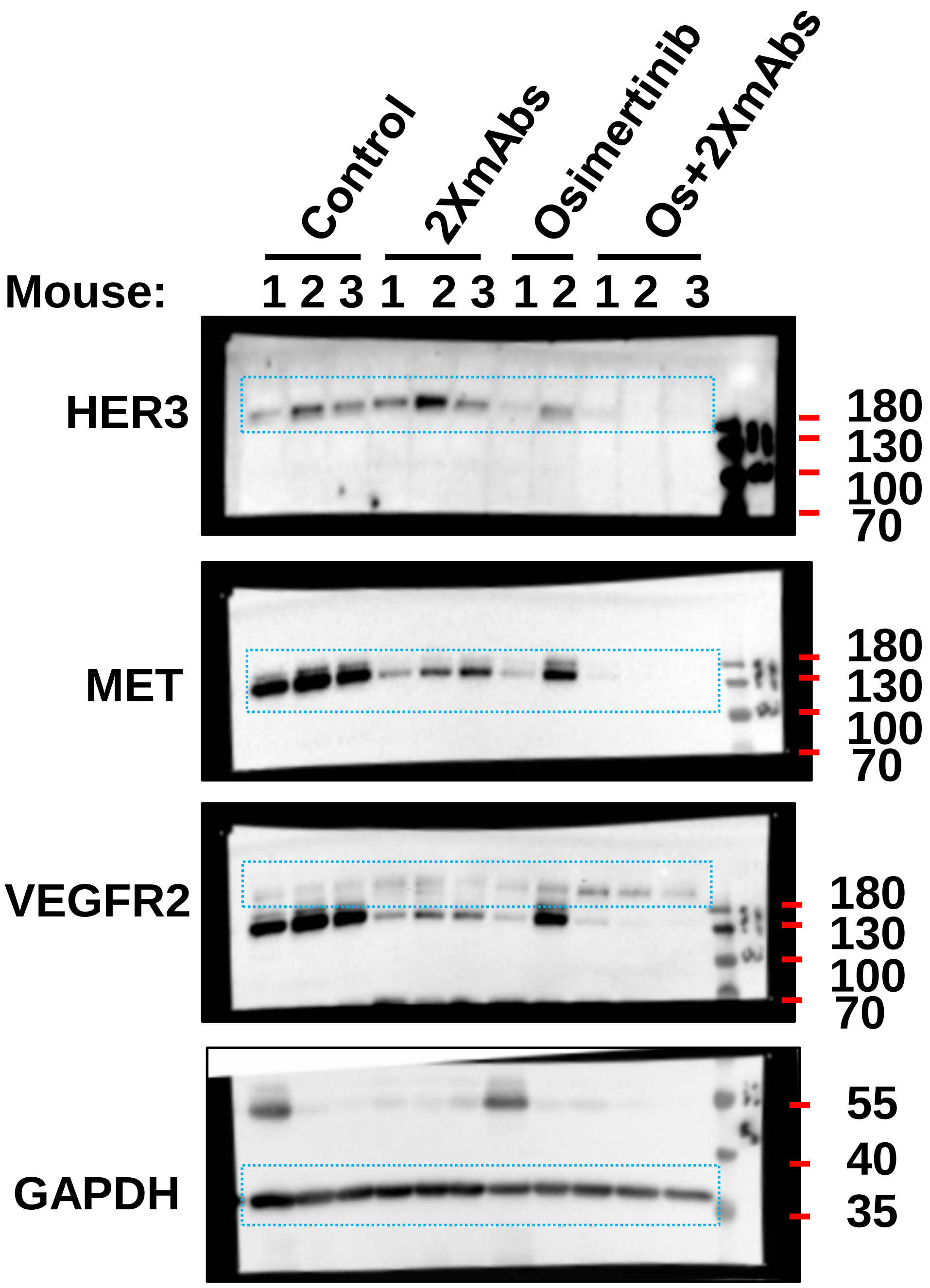
Control
2XmAbs
Osimertinib
Os+2XmAbs
Mouse:
1 2 3 1 2 3 1 2 1 2 3
HER3
MET
VEGFR2
GAPDH
180
130
100
70
180
130
100
70
180
130
100
70
55
40
35

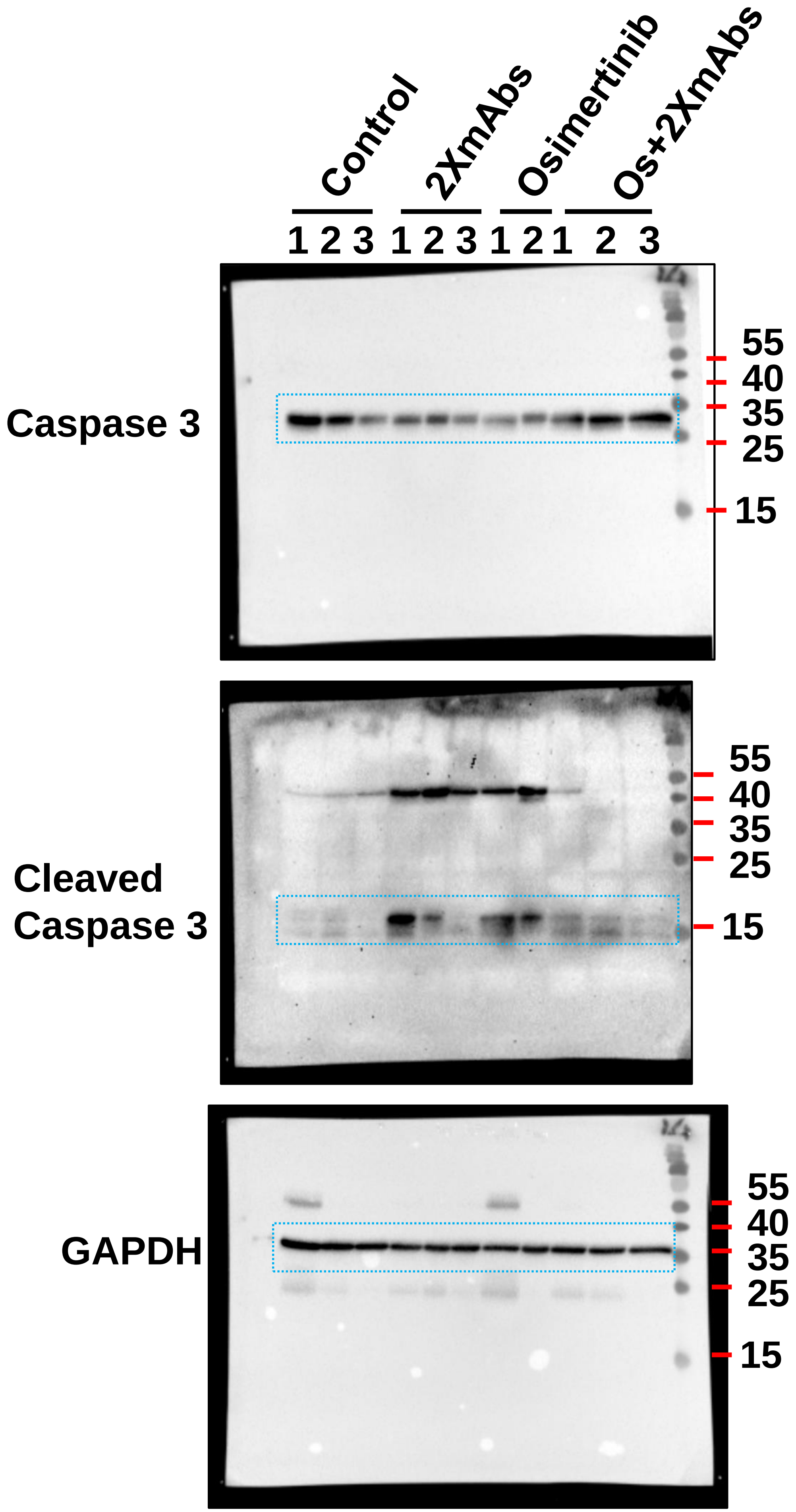

Control
2XmAbs
Osimertinib
Os+2XmAbs
1 2 3 1 2 3 1 2 1 2 3
Caspase 3
55
40
35
25
15
Cleaved
Caspase 3
55
40
35
25
15
GAPDH
55
40
35
25
15

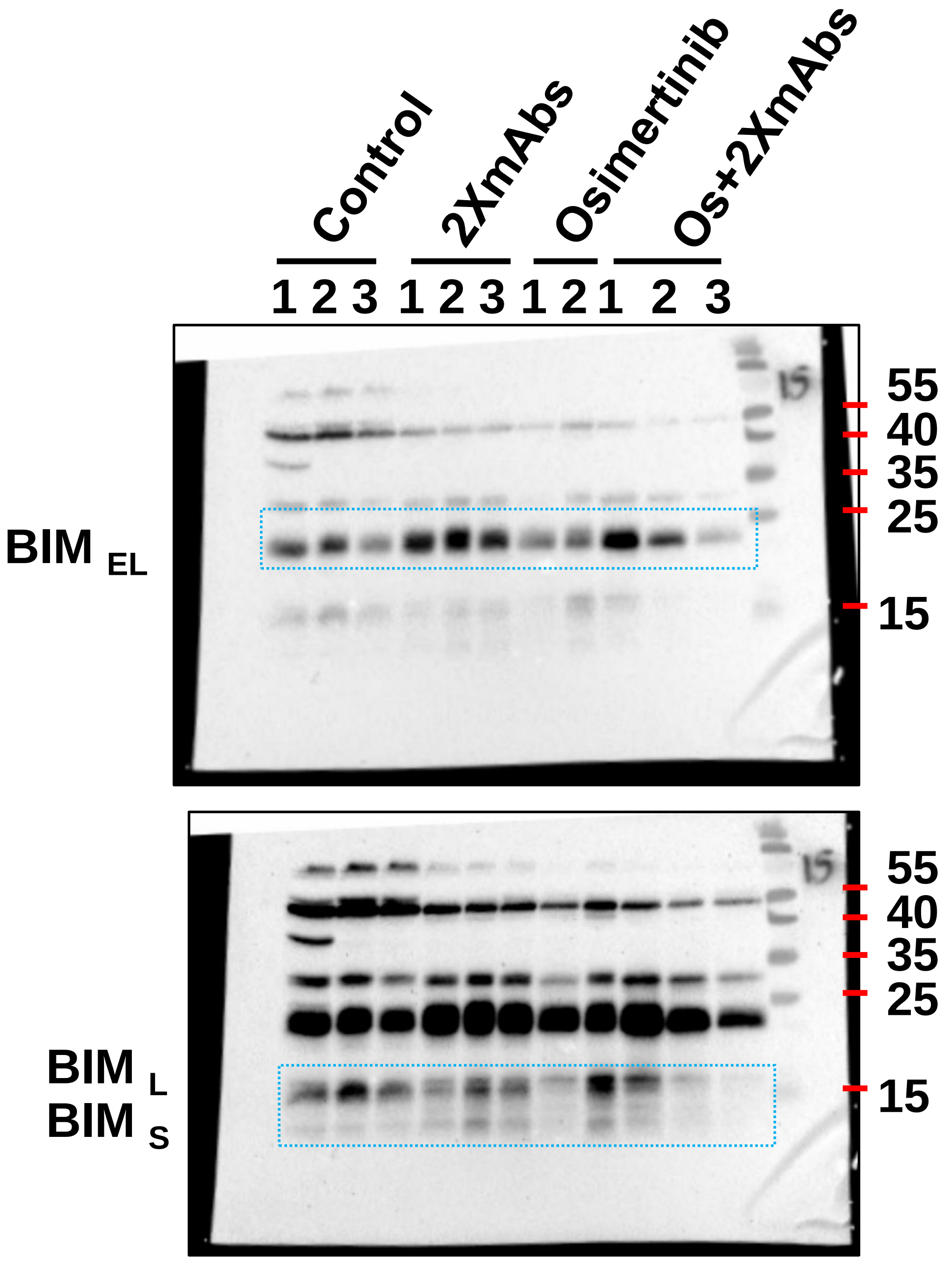
Control
2XmAbs
Osimertinib
Os+2XmAbs
1 2 3 1 2 3 1 2 1 2 3
BIM EL
55
40
35
25
15
BIM L
BIM S
55
40
35
25
15

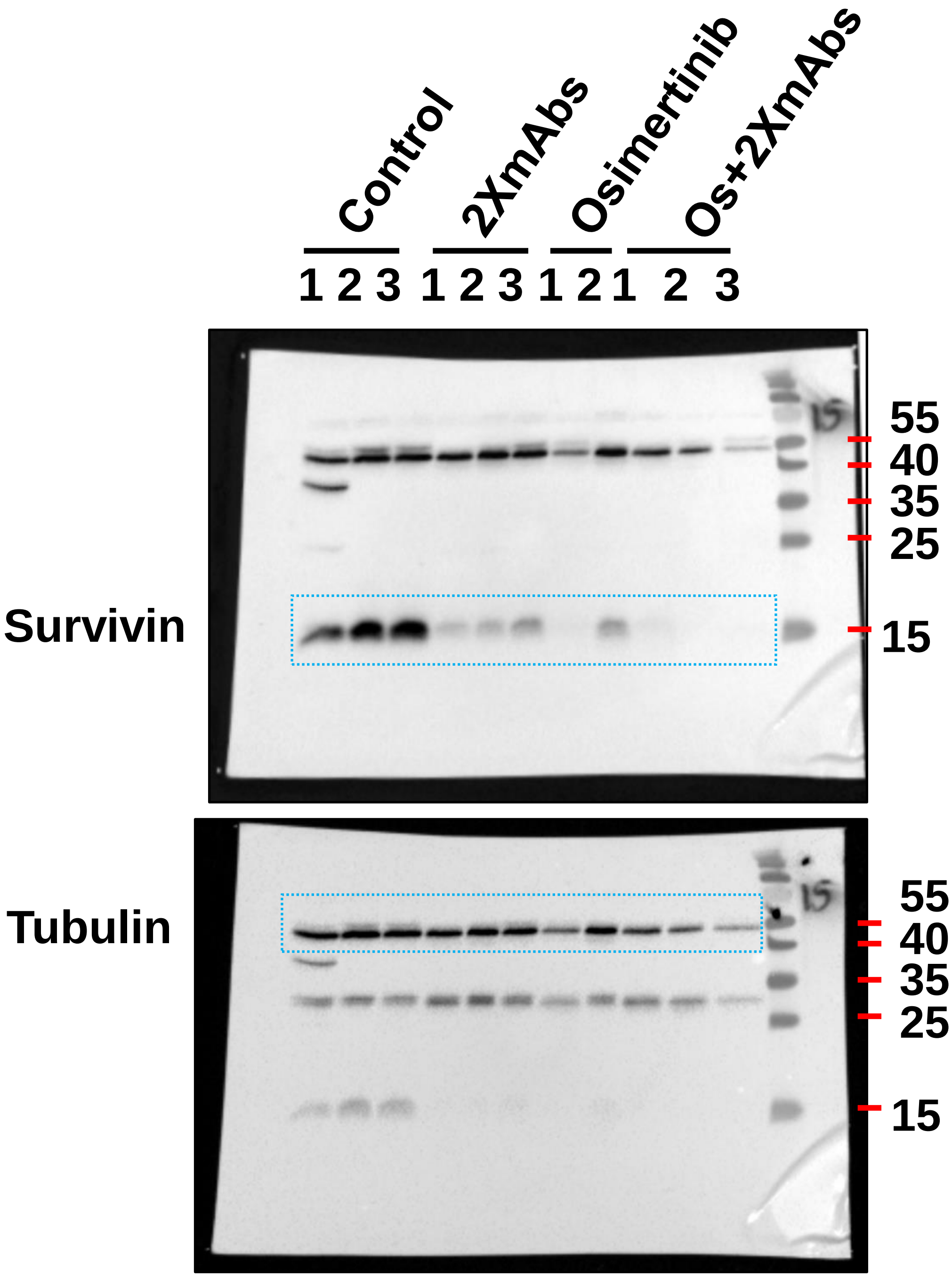

Control
2XmAbs
Osimertinib
Os+2XmAbs
1 2 3 1 2 3 1 2 1 2 3
Survivin
55
40
35
25
15
Tubulin
55
40
35
25
15

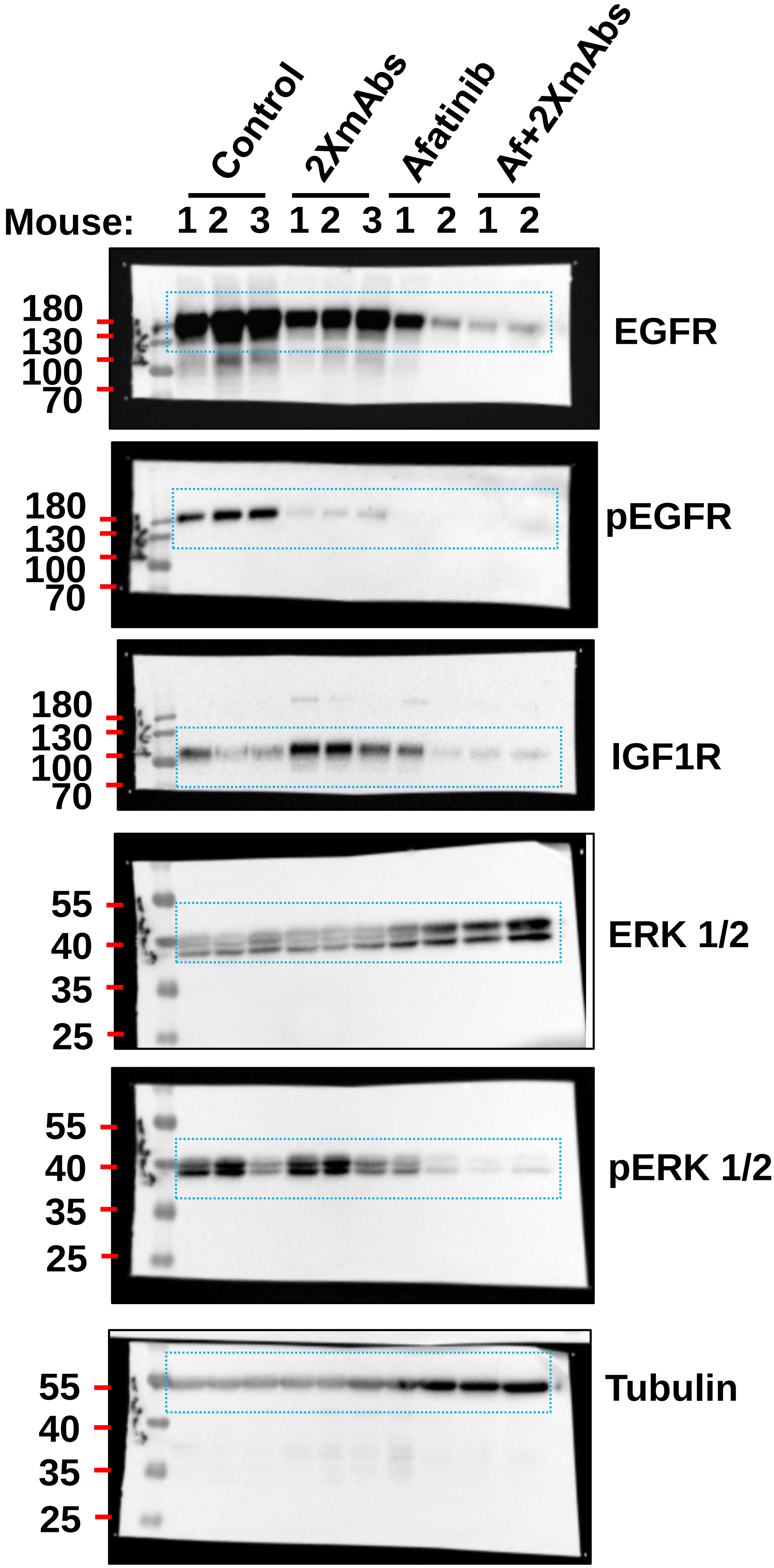
Control
2XmAbs
Afatinib
Af+2XmAbs
Mouse: 1 2 3 1 2 3 1 2 1 2
180
130
100
70
EGFR
pEGFR
IGF1R
55
40
35
25
ERK 1/2
pERK 1/2
Tubulin

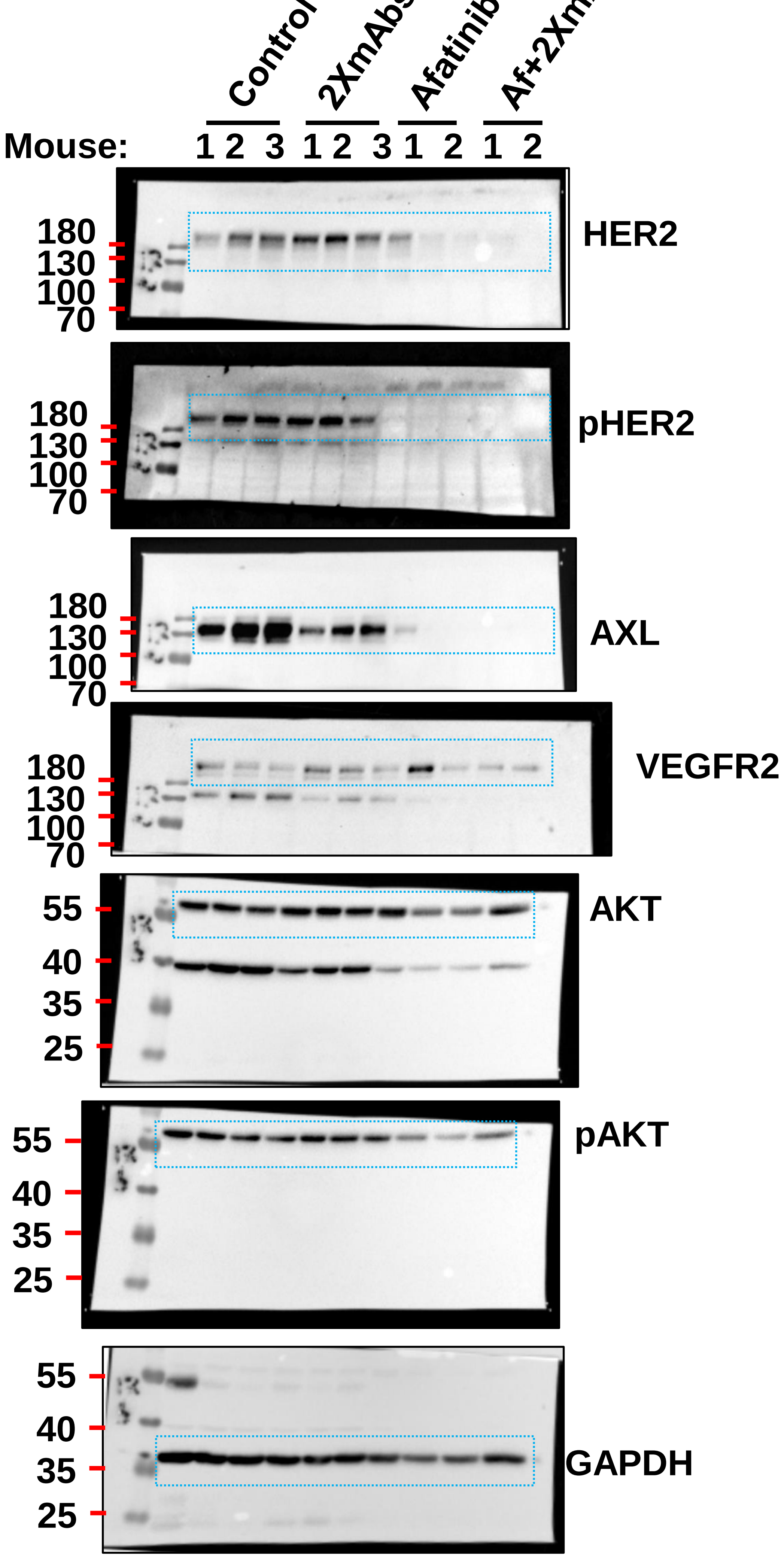

Control
2XmAbs
Afatinib
Af+2Xm
Mouse:
1 2 3 1 2 3 1 2 1 2
HER2
pHER2
AXL
VEGFR2
AKT
pAKT
GAPDH
180
130
100
70
55
40
35
25

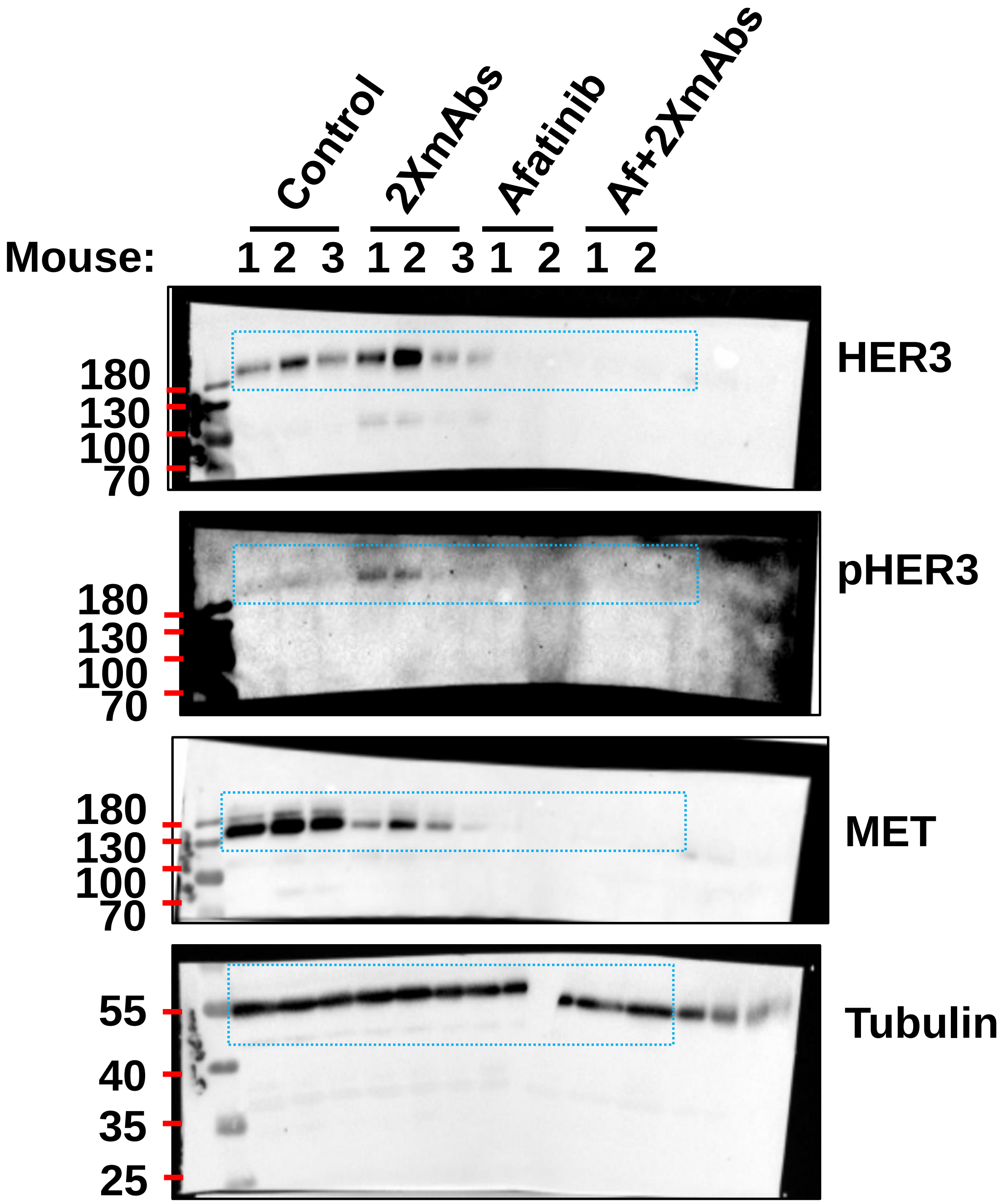

Control
2XmAbs
Afatinib
Af+2XmAbs
Mouse:
1 2 3 1 2 3 1 2 1 2
HER3
pHER3
MET
Tubulin
180
130
100
70
55
40
35
25

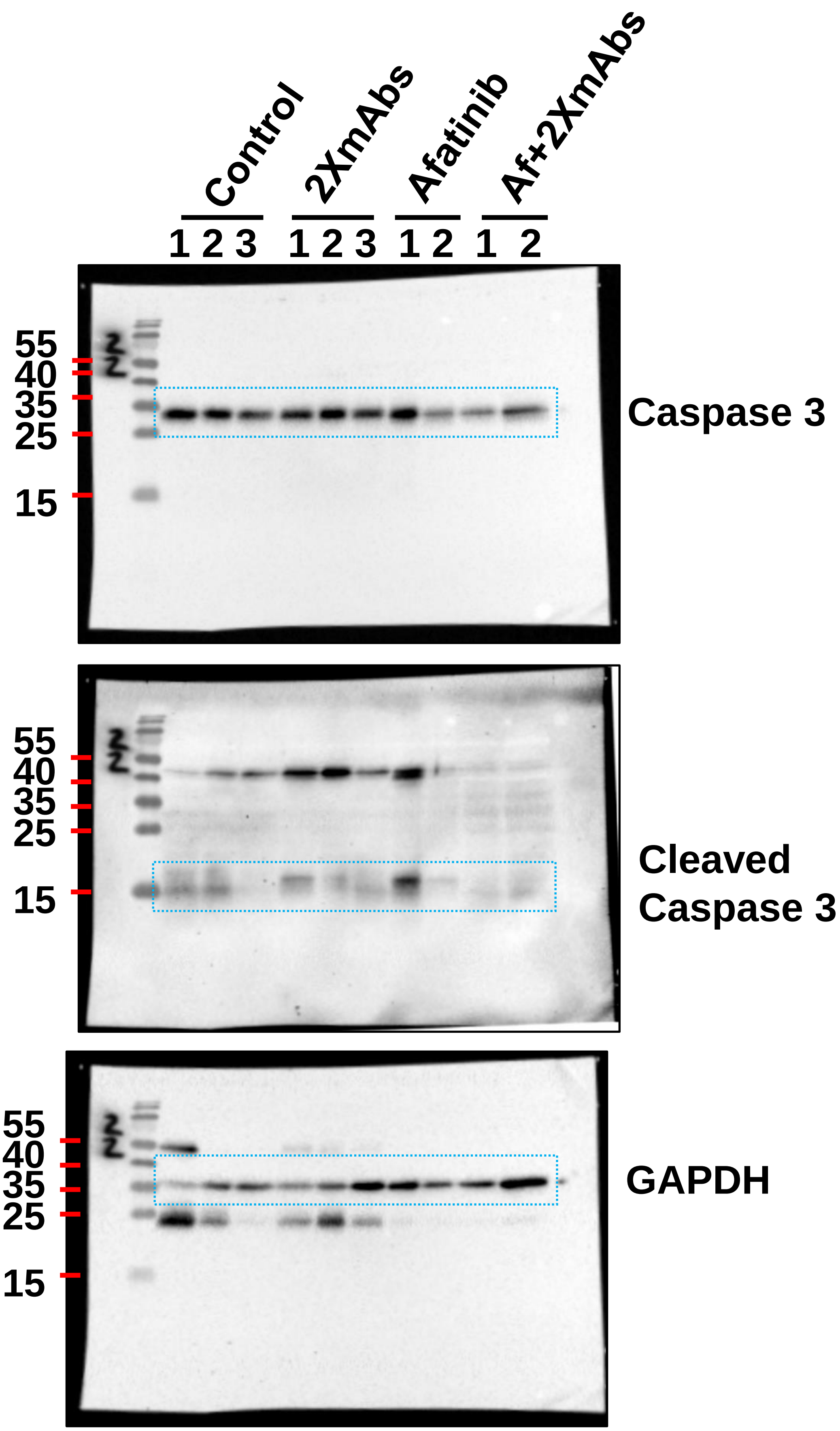

Control
2XmAbs
Afatinib
Af+2XmAbs
1 2 3 1 2 3 1 2 1 2
55
40
35
25
15
Caspase 3
Cleaved Caspase 3
GAPDH

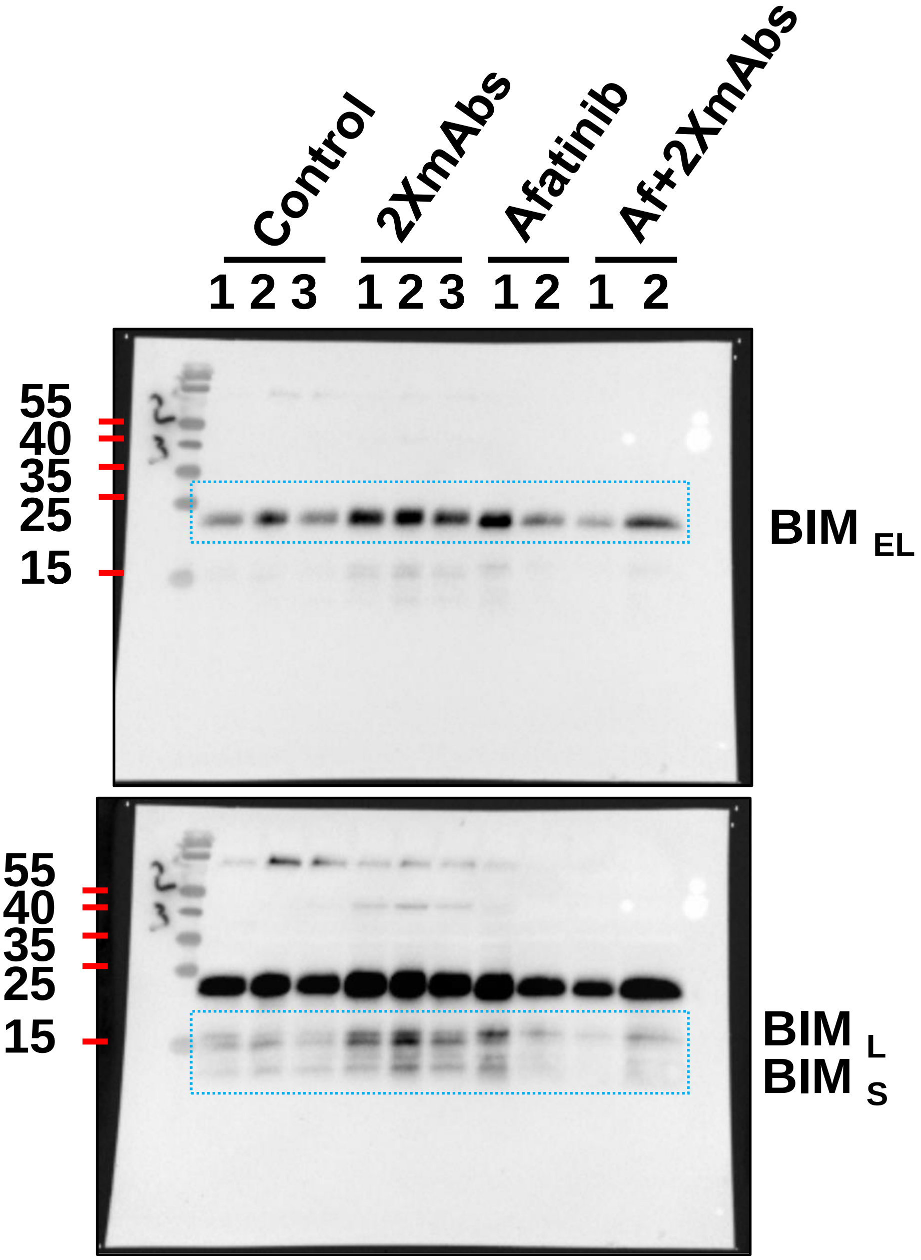

Control
2XmAbs
Afatinib
Af+2XmAbs
1 2 3
1 2 3
1 2
1 2
55
40
35
25
15
BIM EL
55
40
35
25
15
BIM L
BIM S

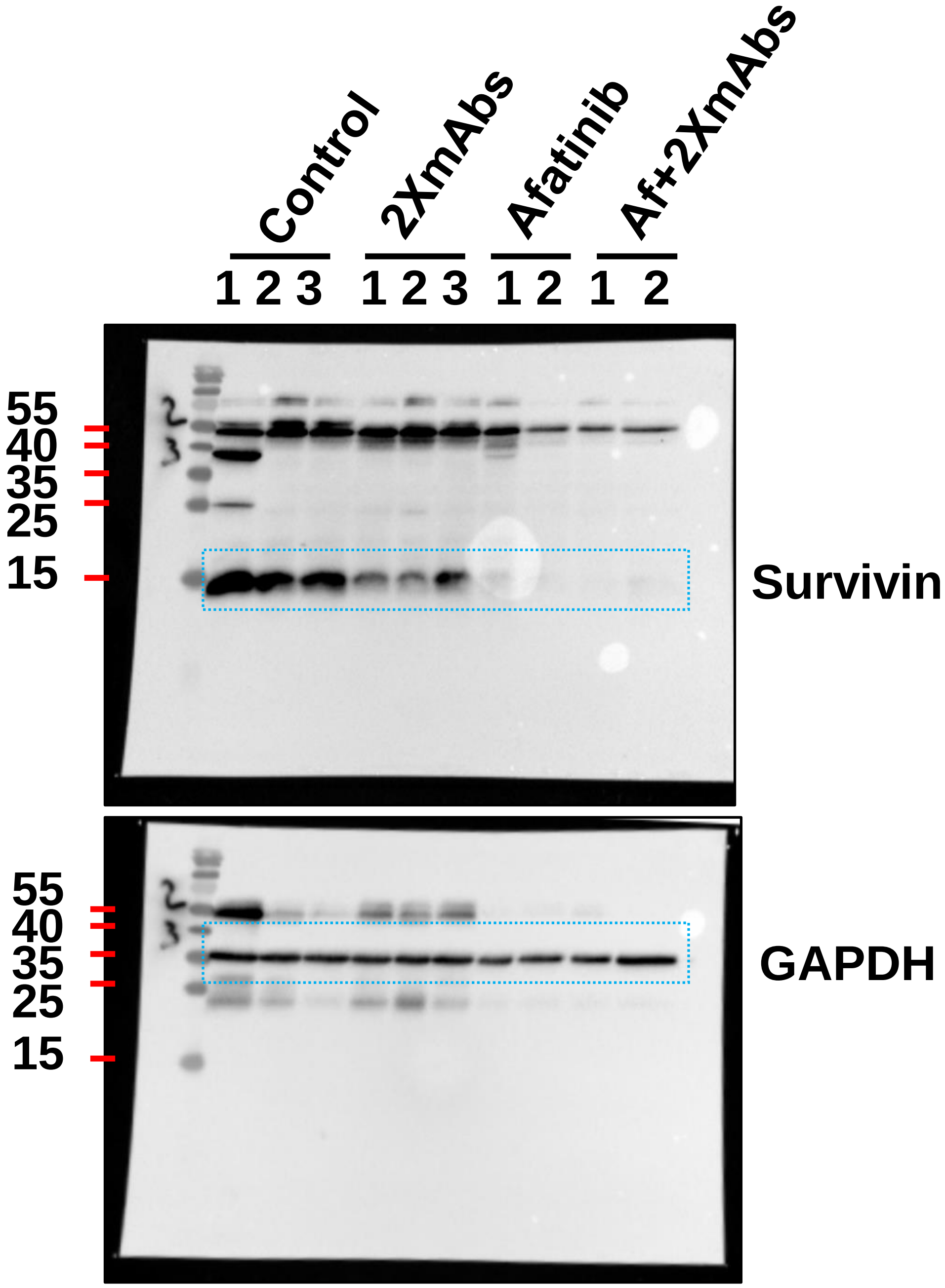

Control
2XmAbs
Afatinib
Af+2XmAbs
1 2 3 1 2 3 1 2 1 2
55
40
35
25
15
Survivin
55
40
35
25
15
GAPDH
