# Supplementary figures and images for: Upfront admixing antibodies and EGFR inhibitors preempts sequential treatments in lung cancer models
Source: EMBO Mol Med. 2021 Mar 4;13(4):e13144. doi: 10.15252/emmm.202013144 (PMC8033519; doi:10.15252/emmm.202013144)

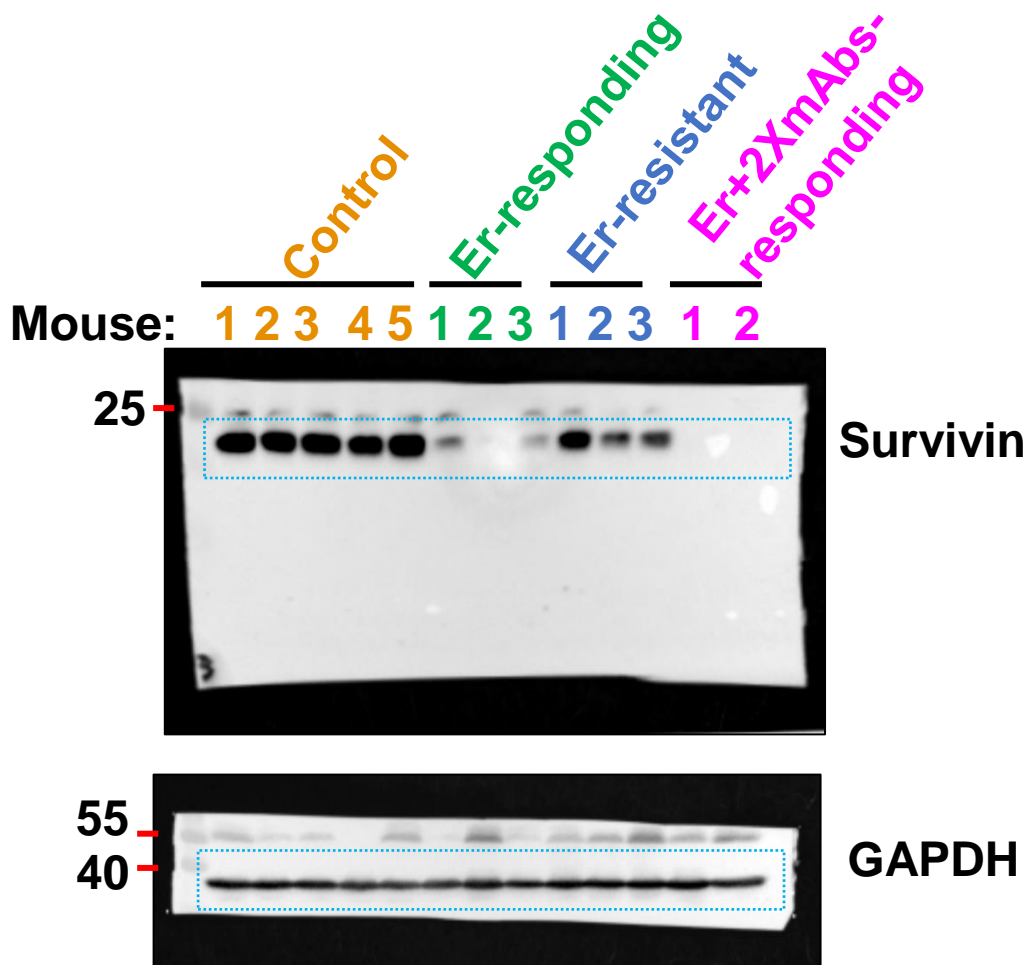

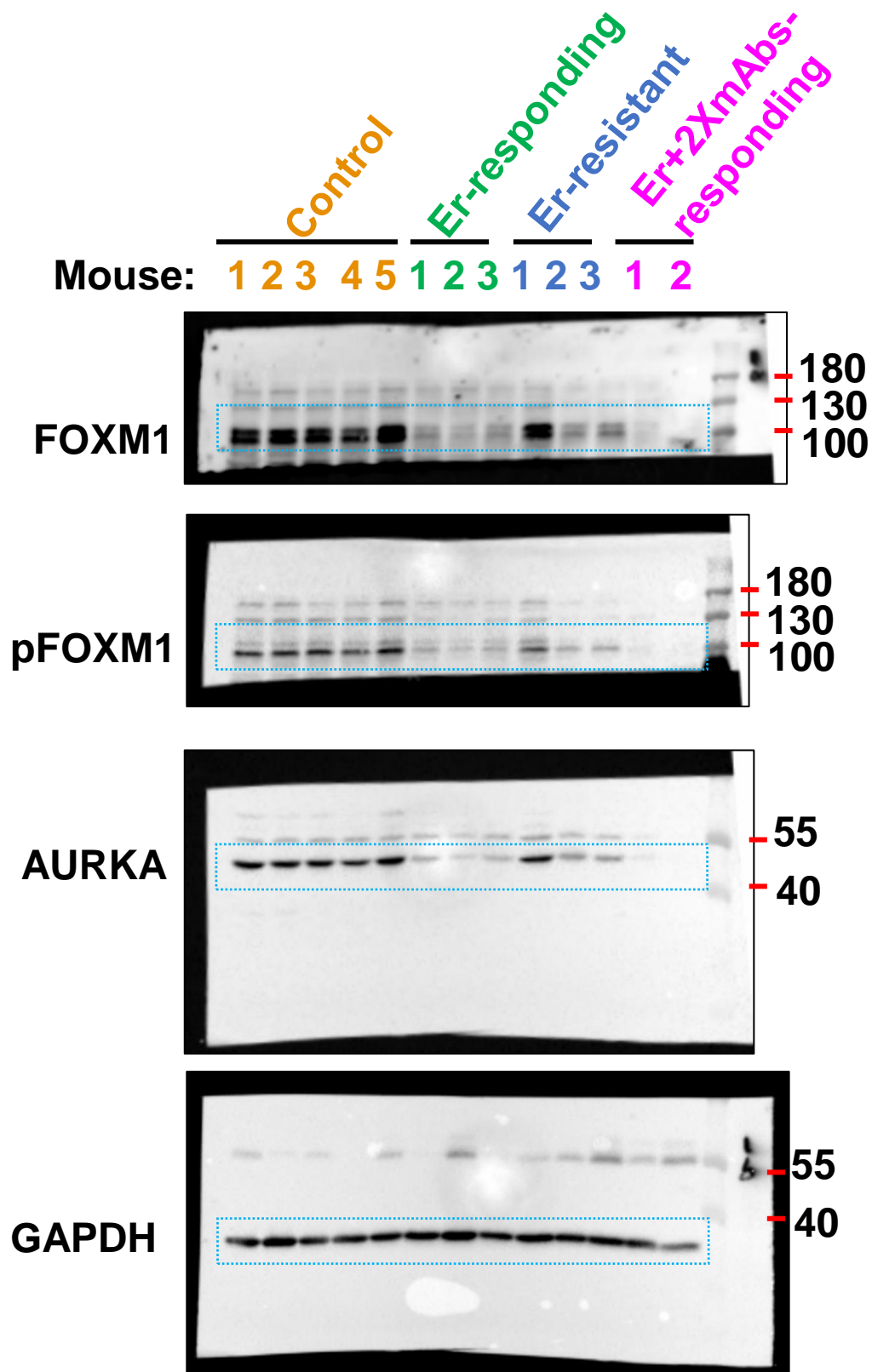

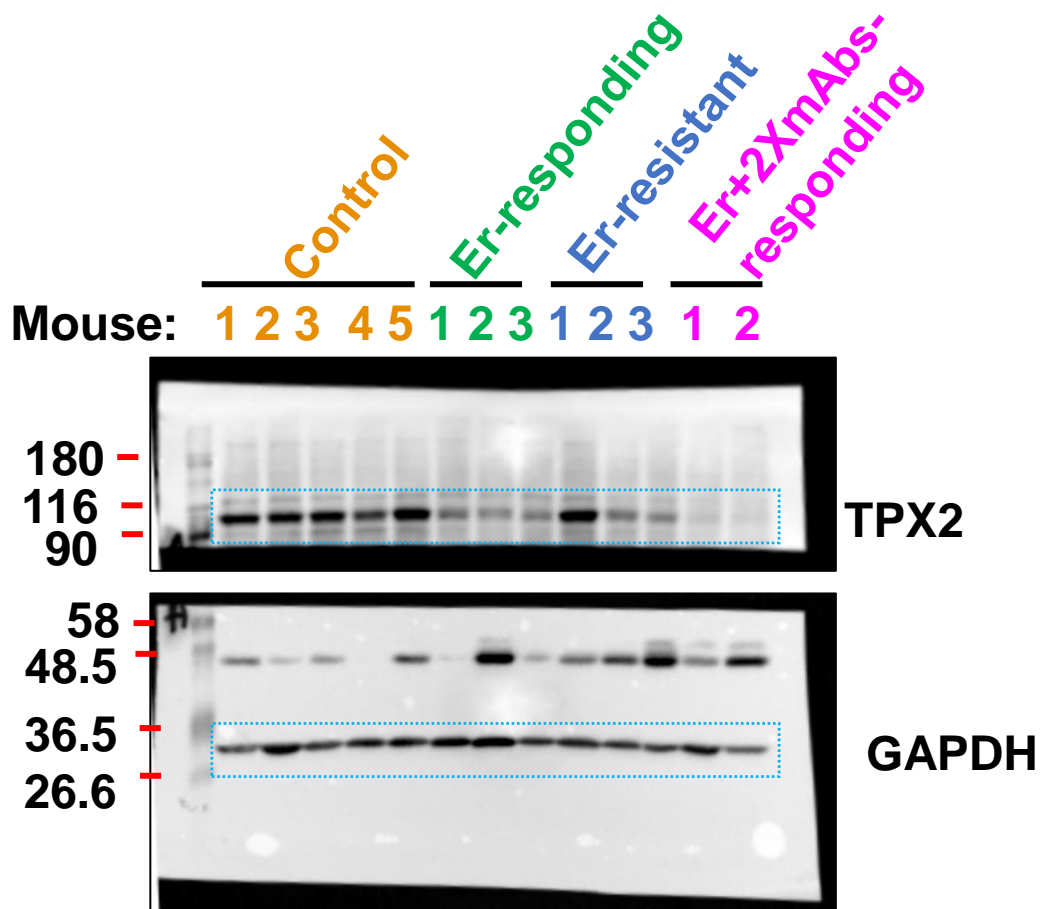

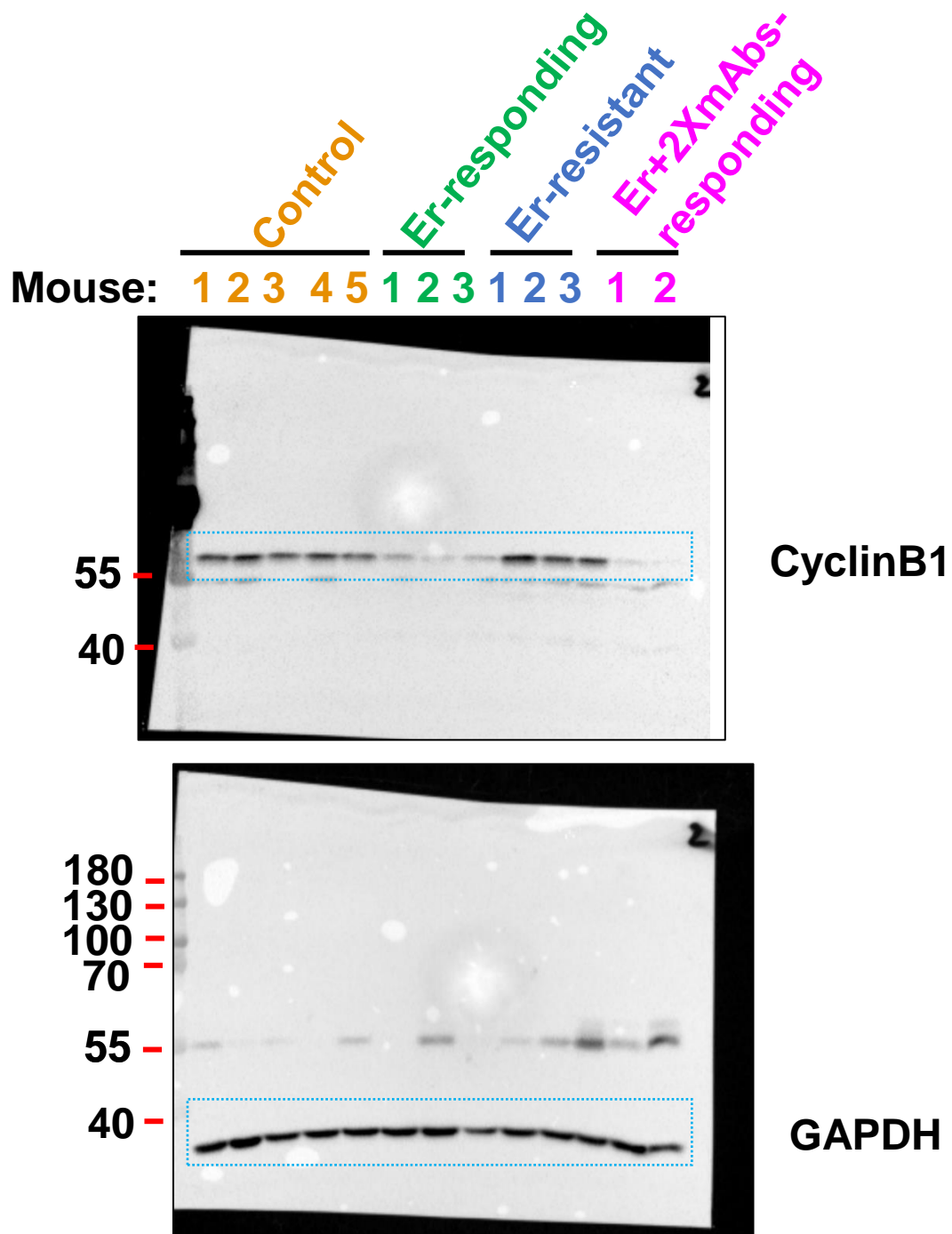

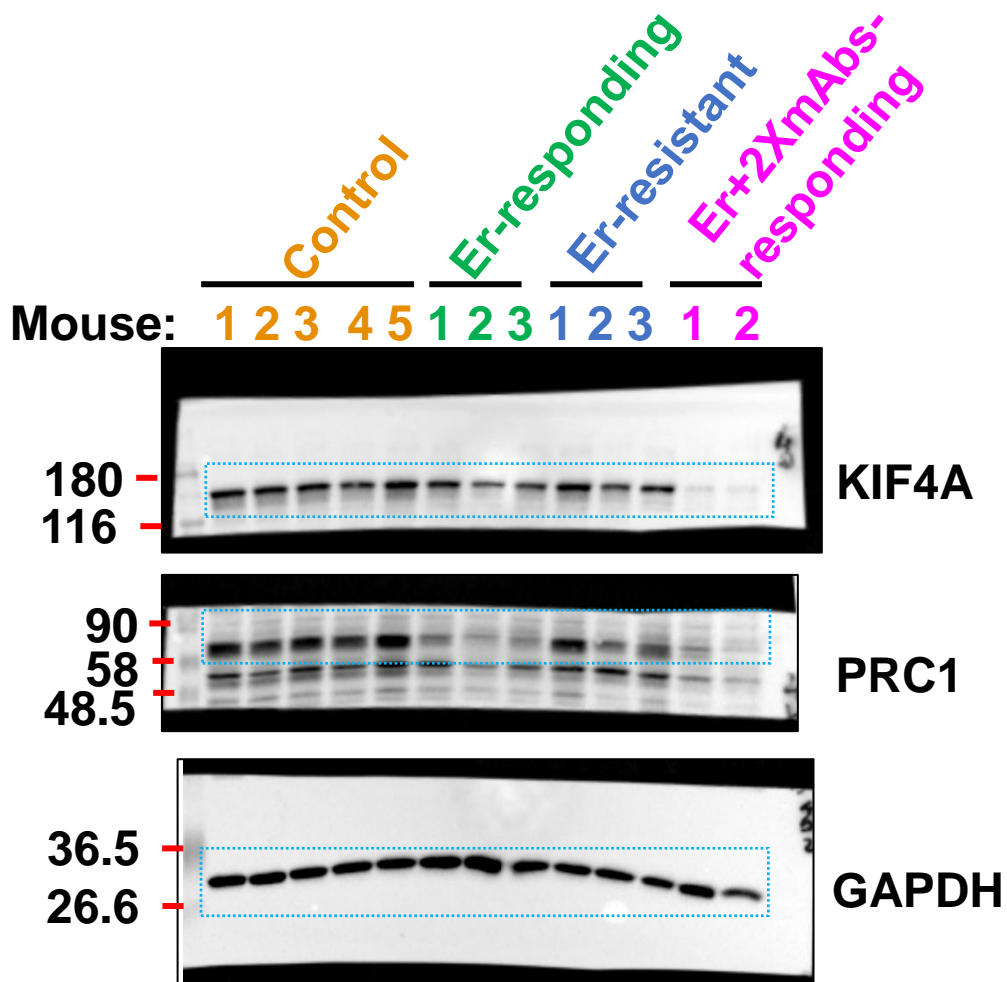

Supplement: Supplementary file 7 — Source Data for Figure 7 [file EMMM-13-e13144-s005.pdf]
