# Supplementary material for: Upfront admixing antibodies and EGFR inhibitors preempts sequential treatments in lung cancer models
Source: EMBO Mol Med. 2021 Mar 4;13(4):e13144. doi: 10.15252/emmm.202013144 (PMC8033519; doi:10.15252/emmm.202013144)

Appendix Figure S1A

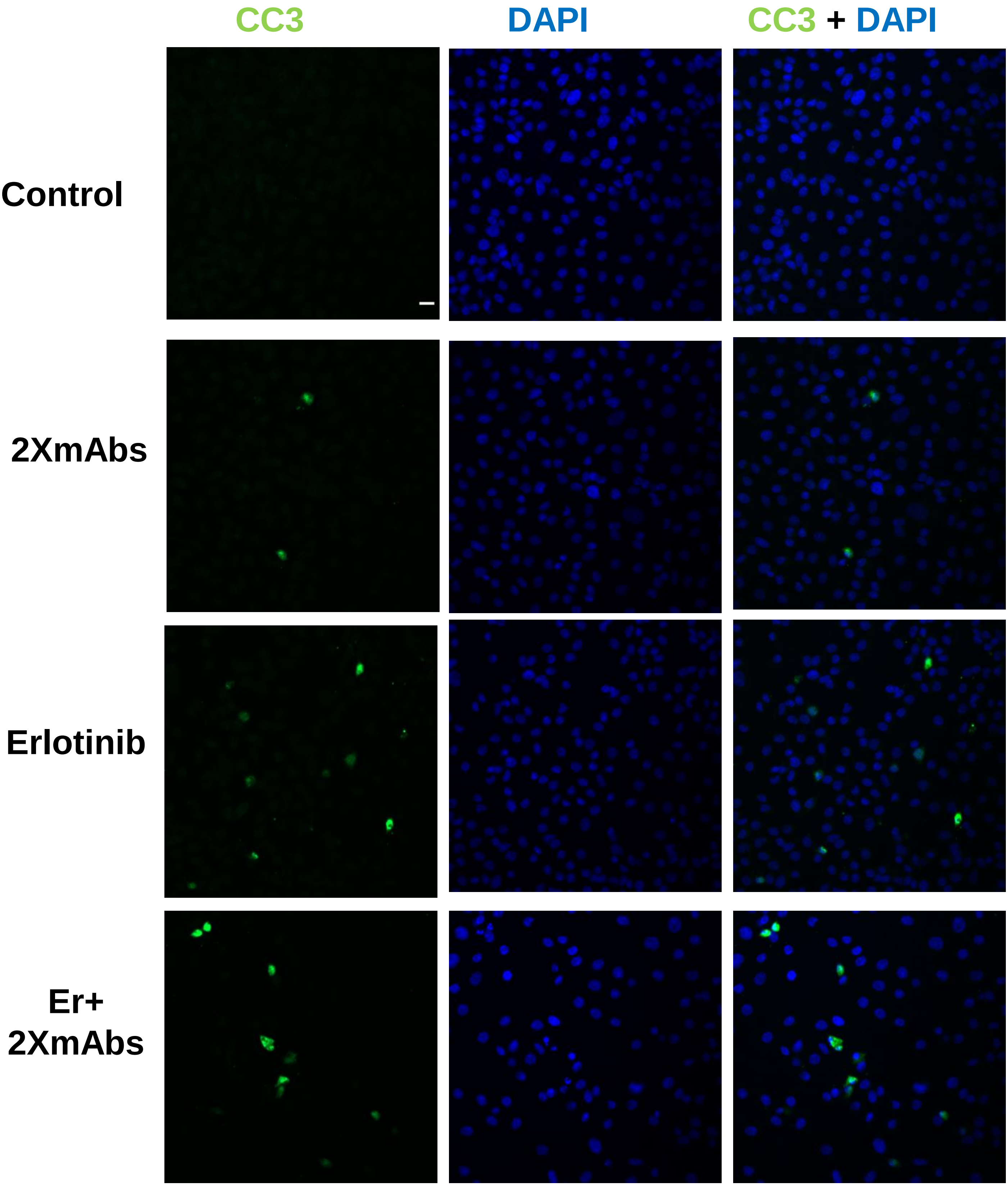

Appendix Figure S1A

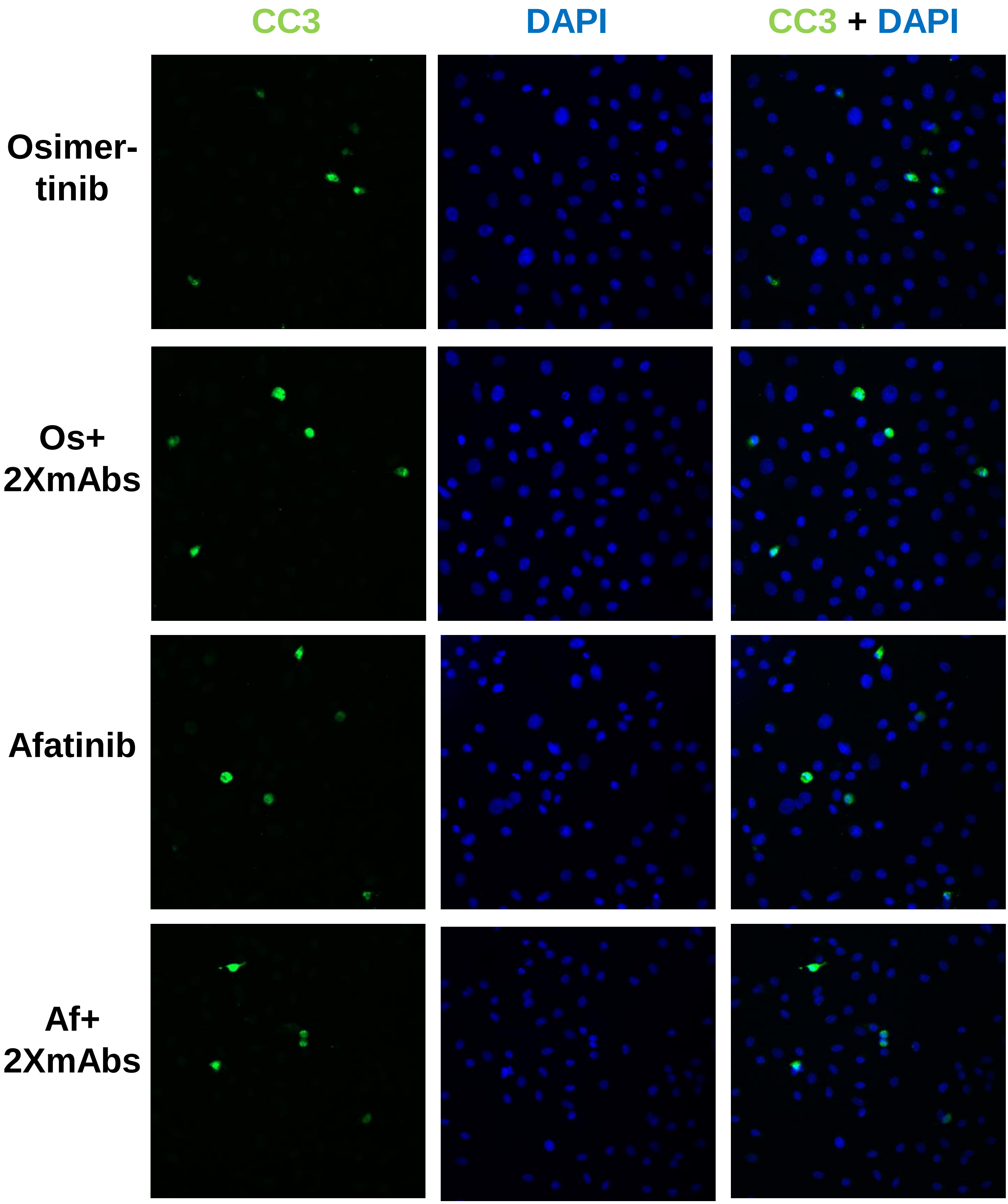

Appendix Figure S1C

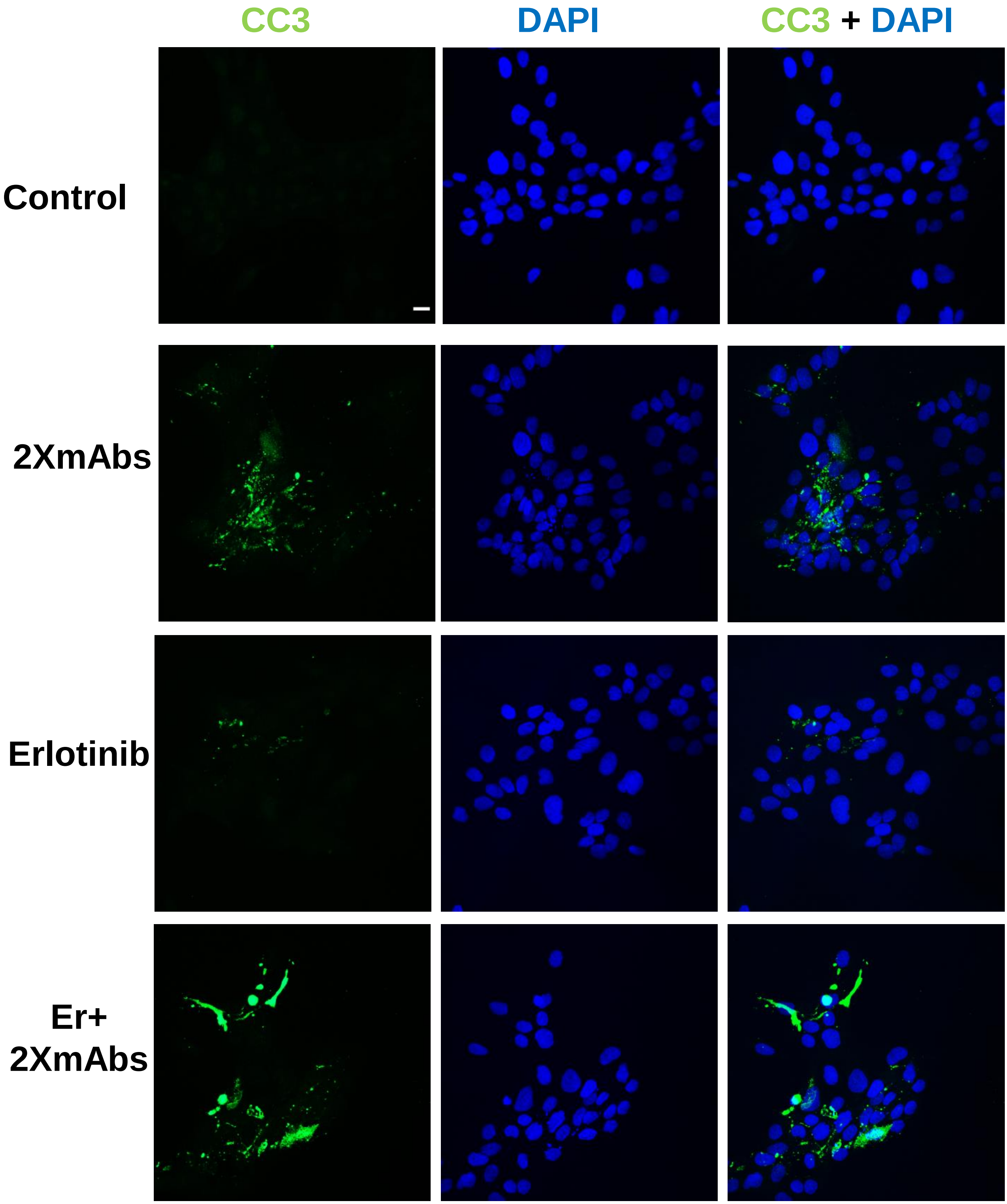

Appendix Figure S1C

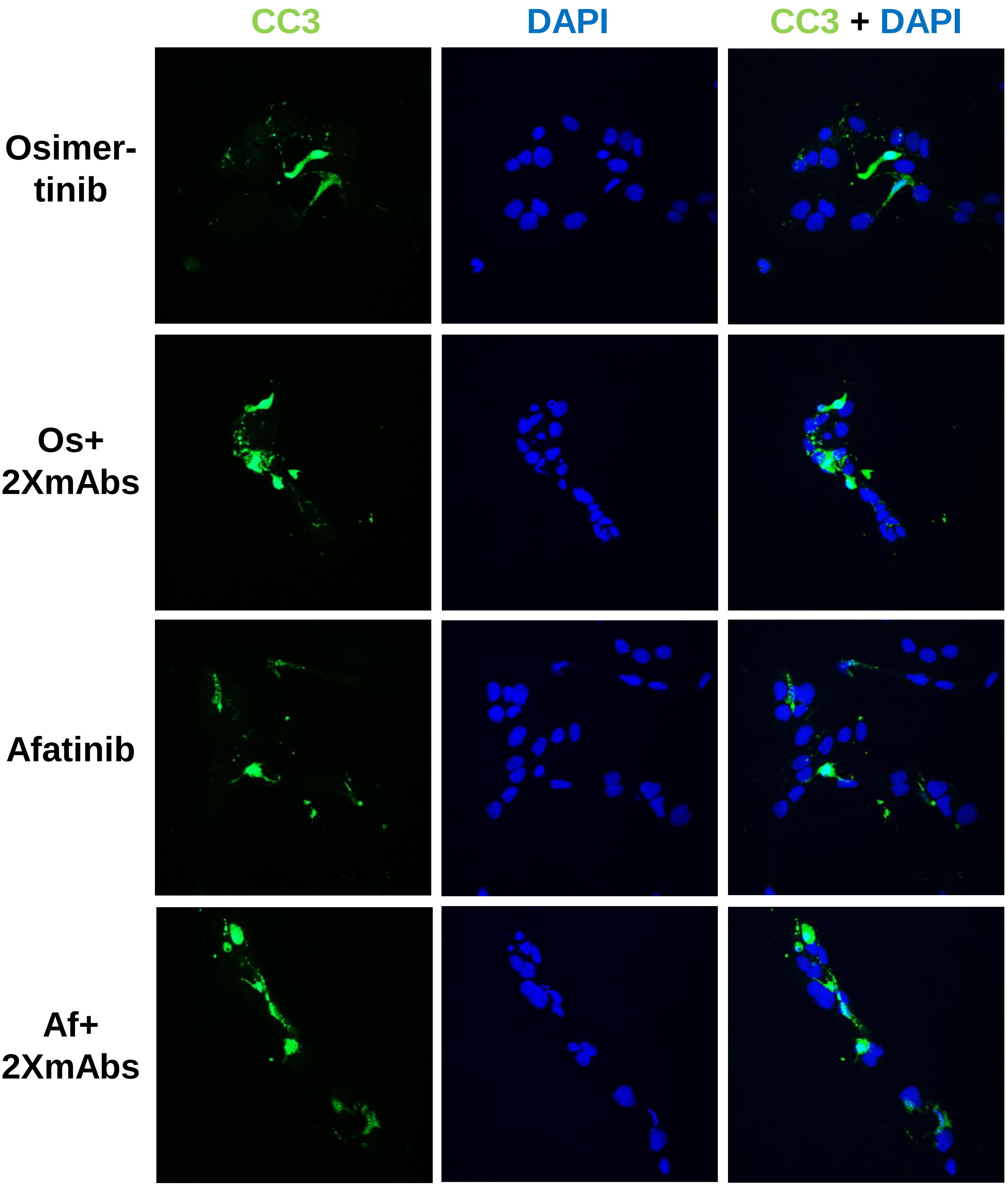

Supplement: Supplementary file 3 — Source Data for Expanded View and Appendix [file EMMM-13-e13144-s003.zip › SourceDataForAppendixFigures.zip!/SourceDataForAppendixFigureS1A_C.pdf]
