# Supplementary material for: Upfront admixing antibodies and EGFR inhibitors preempts sequential treatments in lung cancer models
Source: EMBO Mol Med. 2021 Mar 4;13(4):e13144. doi: 10.15252/emmm.202013144 (PMC8033519; doi:10.15252/emmm.202013144)
Supplement: Supplementary file 3 — Source Data for Expanded View and Appendix [file EMMM-13-e13144-s003.zip › SourceDataForAppendixFigures.zip!/SourceDataForAppendixFigureS6.pdf]

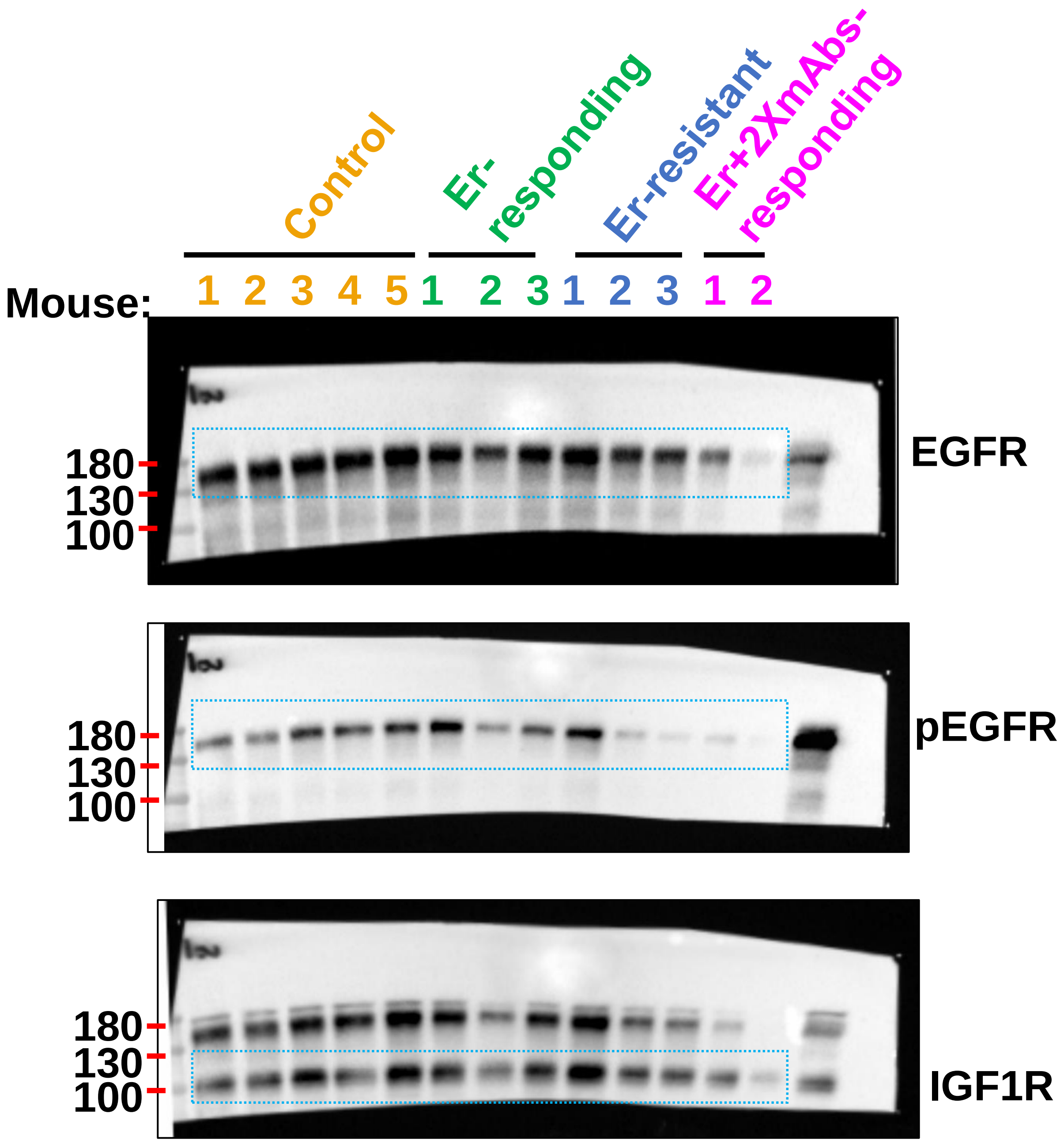
Control
Er-responding
Er-resistant
Er+2XmAbs-responding
Mouse:
1 2 3 4 5 1 2 3 1 2 3 1 2
180
130
100
EGFR
pEGFR
IGF1R

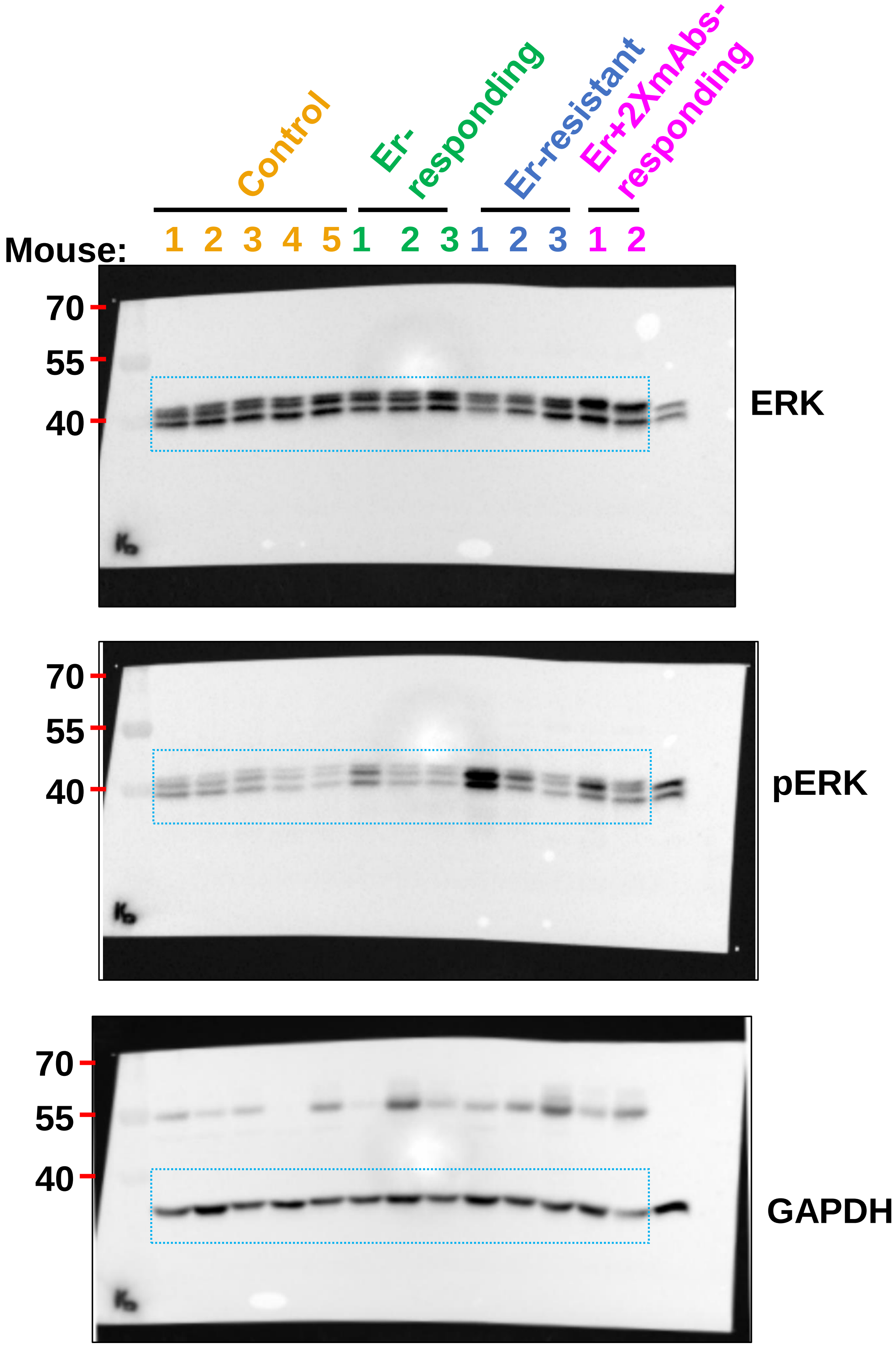
Control
Er-responding
Er-resistant
Er+2XmAbs-responding
Mouse:
1 2 3 4 5 1 2 3 1 2 3 1 2
70
55
40
ERK
70
55
40
pERK
70
55
40
GAPDH

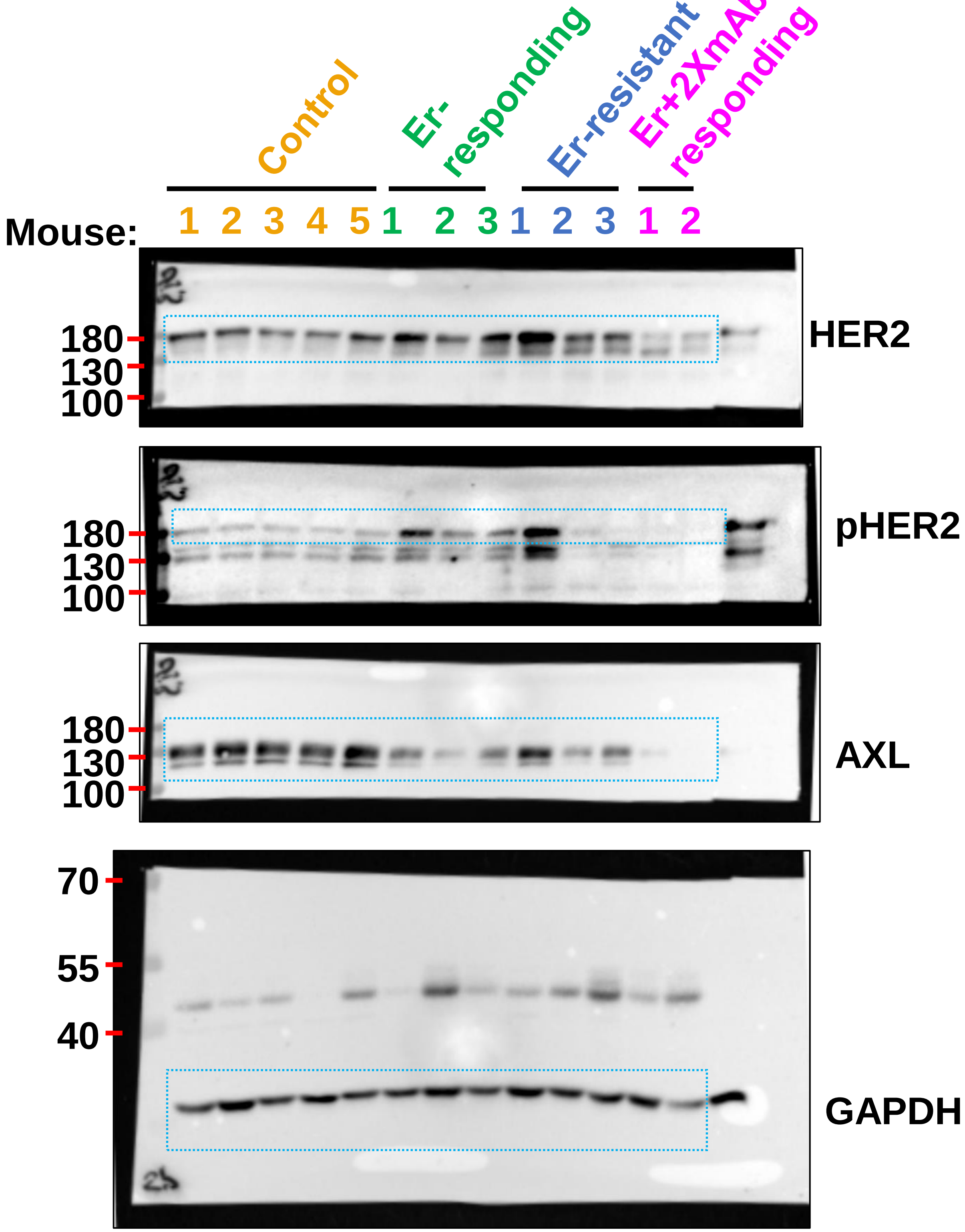
Control
Er-responding
Er-resistant
Er+2XmAb responding
Mouse: 1 2 3 4 5 1 2 3 1 2 3 1 2
180
130
100
HER2
pHER2
AXL
70
55
40
GAPDH

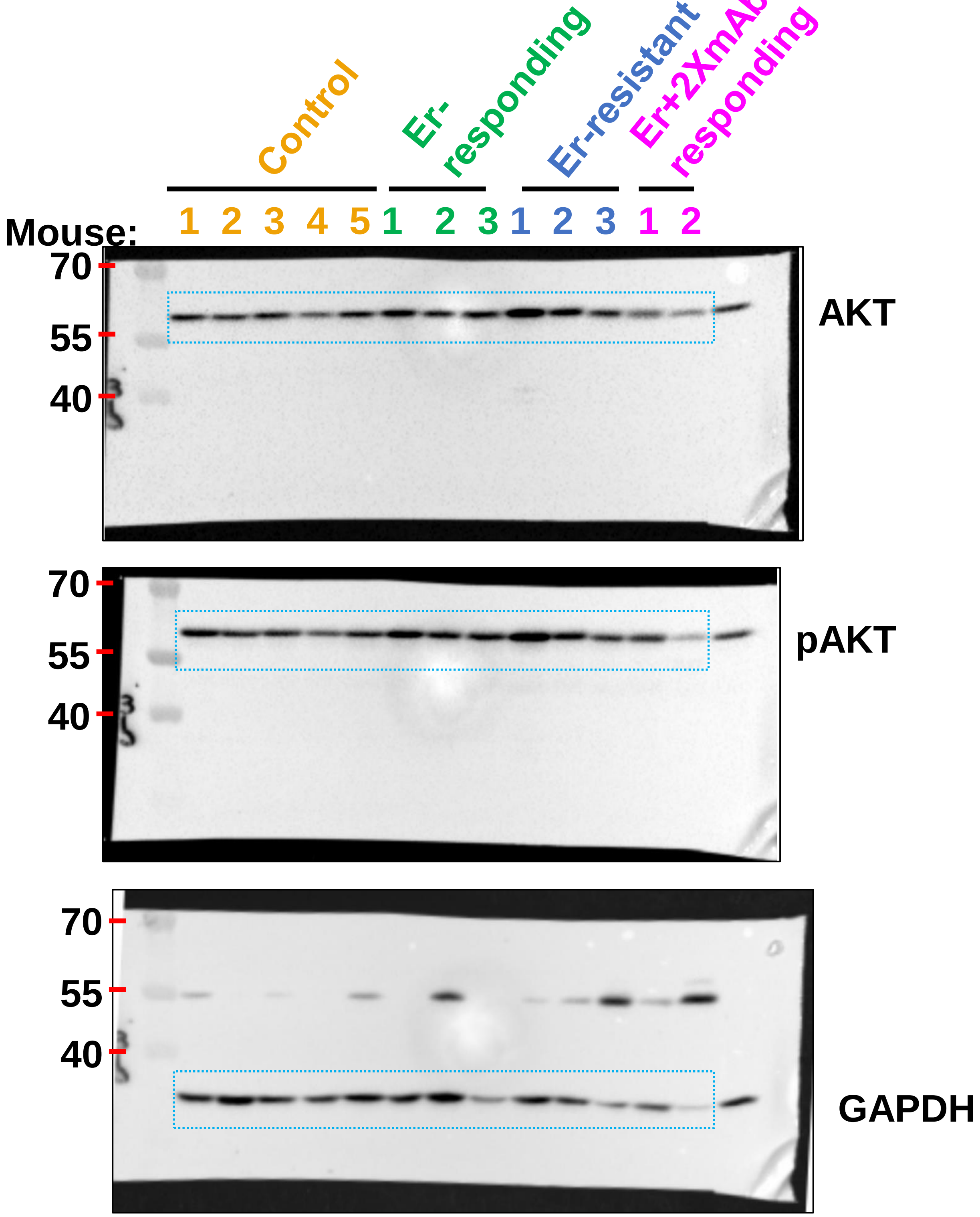

Control
Er-
responding
Er-resistant
Er+2XmAb
responding
Mouse:
1 2 3 4 5 1 2 3 1 2 3 1 2
70
55
40
AKT
70
55
40
pAKT
70
55
40
GAPDH

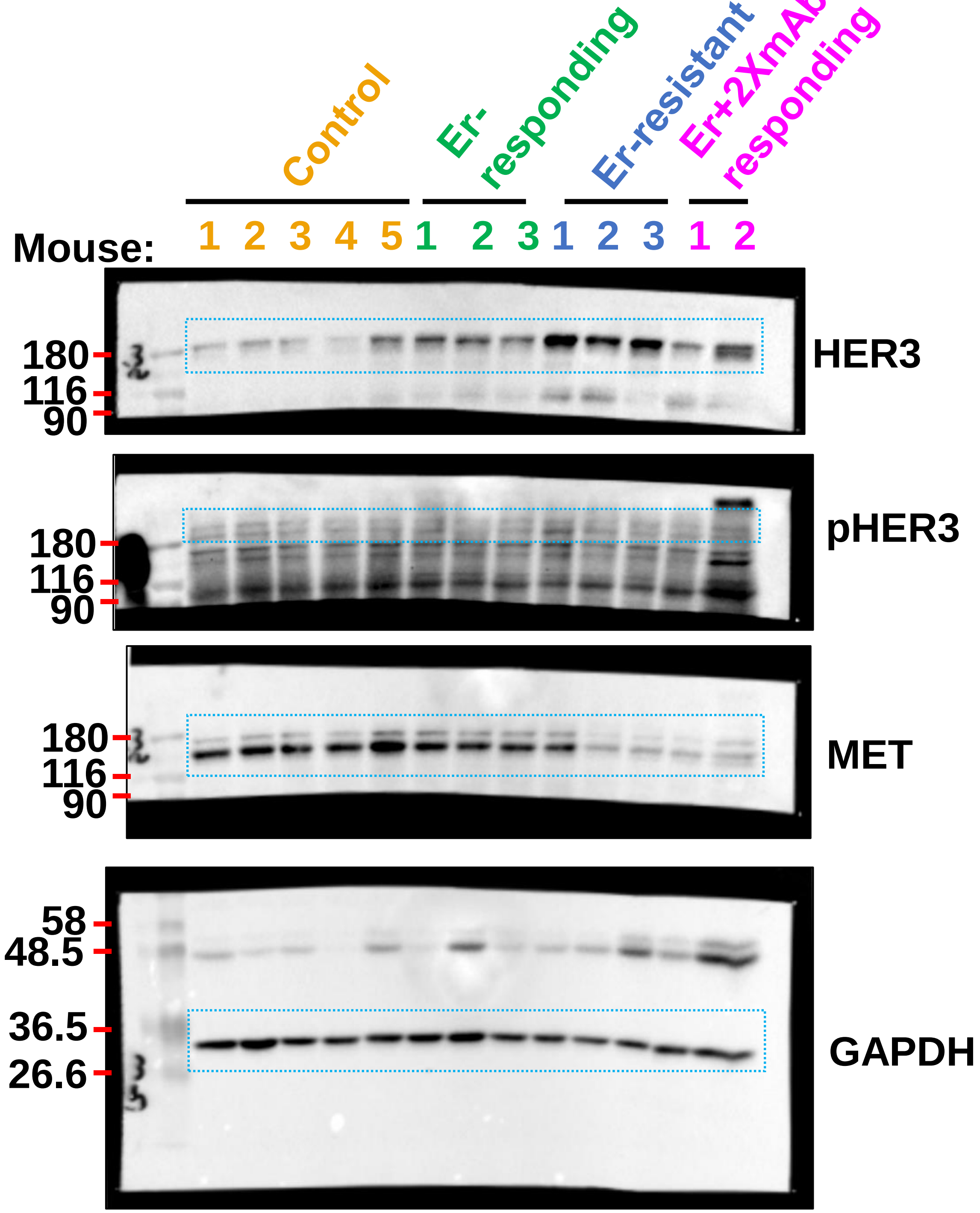

Control
Er-
responding
Er-resistant
Er+2XmAb
responding
Mouse:
1 2 3 4 5 1 2 3 1 2 3 1 2
180
116
90
HER3
180
116
90
pHER3
180
116
90
MET
58
48.5
36.5
26.6
GAPDH
