# Supplementary material for: Upfront admixing antibodies and EGFR inhibitors preempts sequential treatments in lung cancer models
Source: EMBO Mol Med. 2021 Mar 4;13(4):e13144. doi: 10.15252/emmm.202013144 (PMC8033519; doi:10.15252/emmm.202013144)
Supplement: Supplementary file 3 — Source Data for Expanded View and Appendix [file EMMM-13-e13144-s003.zip › SourceDataForEVFigures.zip!/SourceDataForFigureEV4B_C.pdf]

Figure EV4B

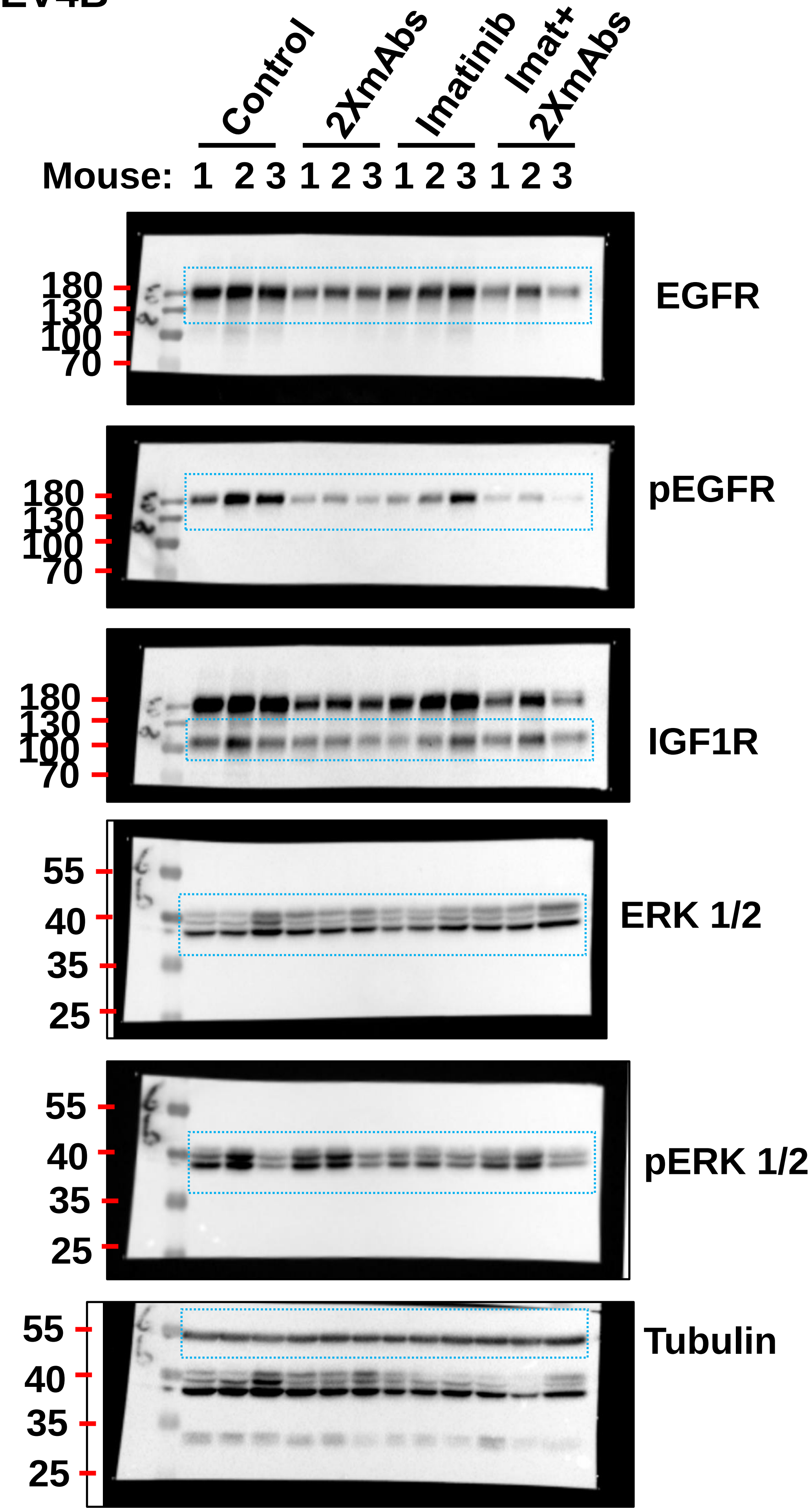

Control
2XmAbs
Imatinib
Imat+ 2XmAbs
Mouse: 1 2 3 1 2 3 1 2 3 1 2 3
180
130
100
70
EGFR
180
130
100
70
pEGFR
180
130
100
70
IGF1R
55
40
35
25
ERK 1/2
55
40
35
25
pERK 1/2
55
40
35
25
Tubulin

Figure EV4B

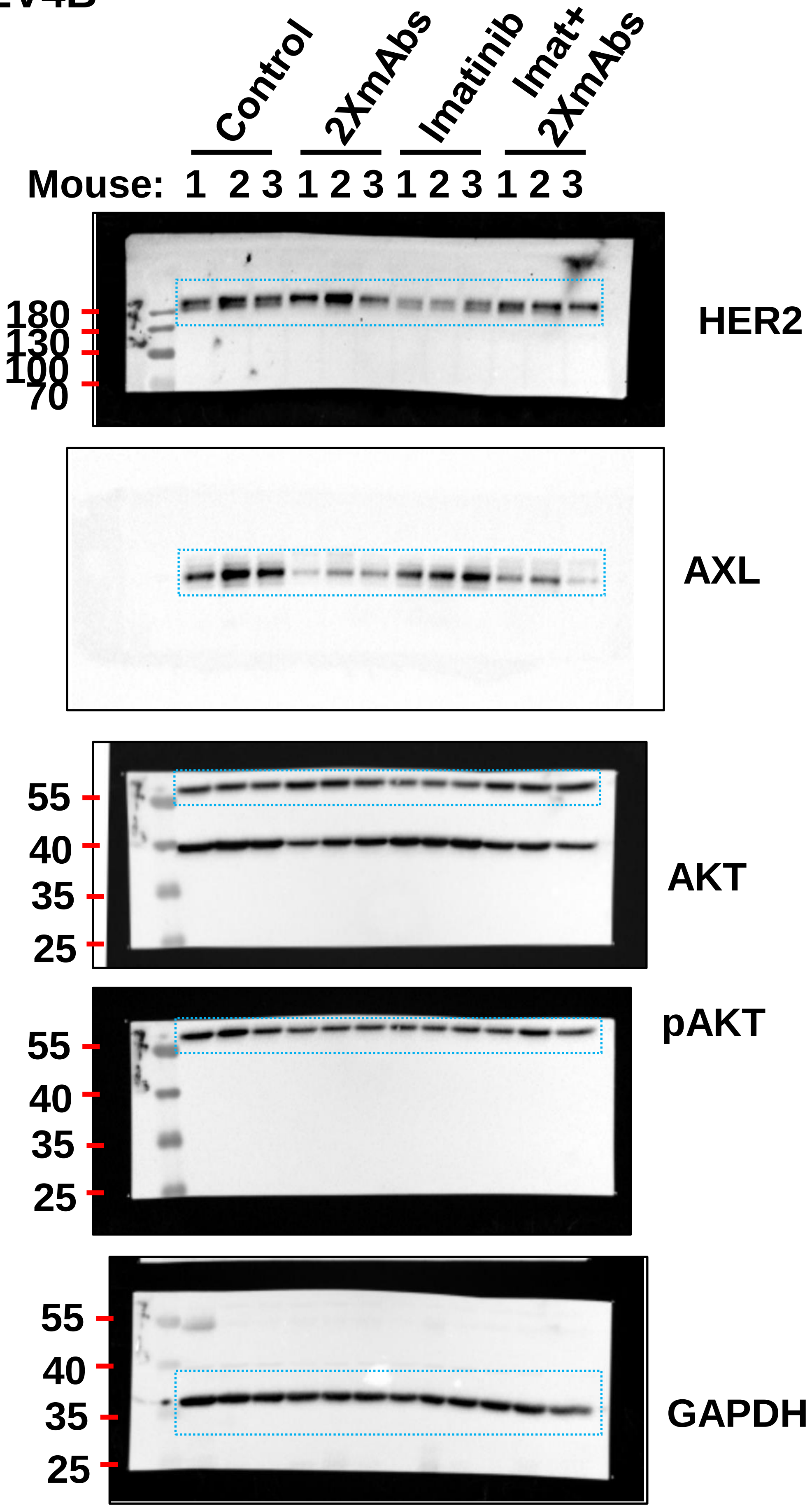

Control
2XmAbs
Imatinib
Imat+ 2XmAbs
Mouse: 1 2 3 1 2 3 1 2 3 1 2 3
180
130
100
70
HER2
AXL
55
40
35
25
AKT
pAKT
55
40
35
25
55
40
35
25
GAPDH

Figure EV4B

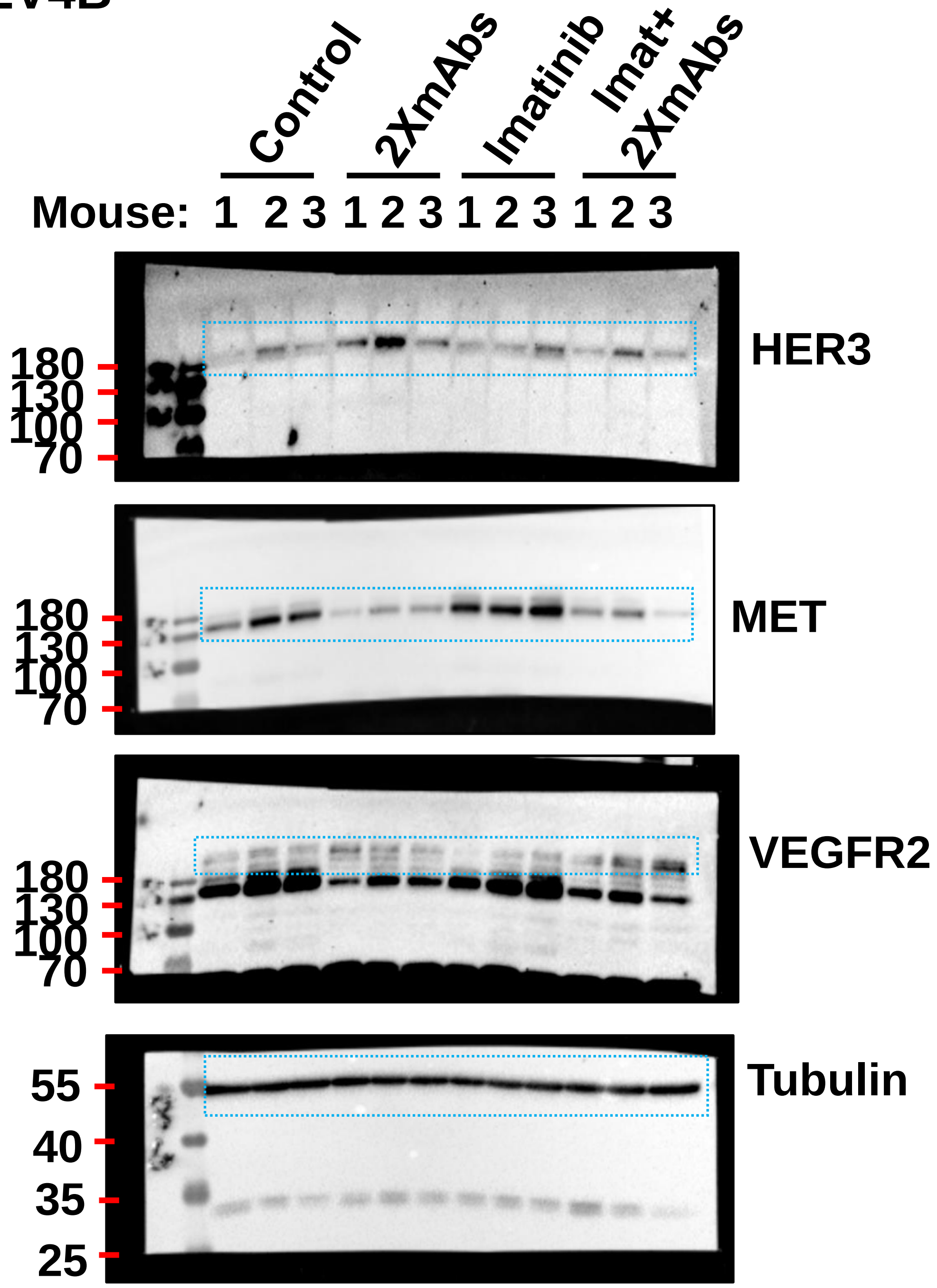

Control
2XmAbs
Imatinib
Imat+
2XmAbs
Mouse: 1 2 3 1 2 3 1 2 3 1 2 3
180
130
100
70
HER3
180
130
100
70
MET
180
130
100
70
VEGFR2
55
40
35
25
Tubulin

Figure EV4B

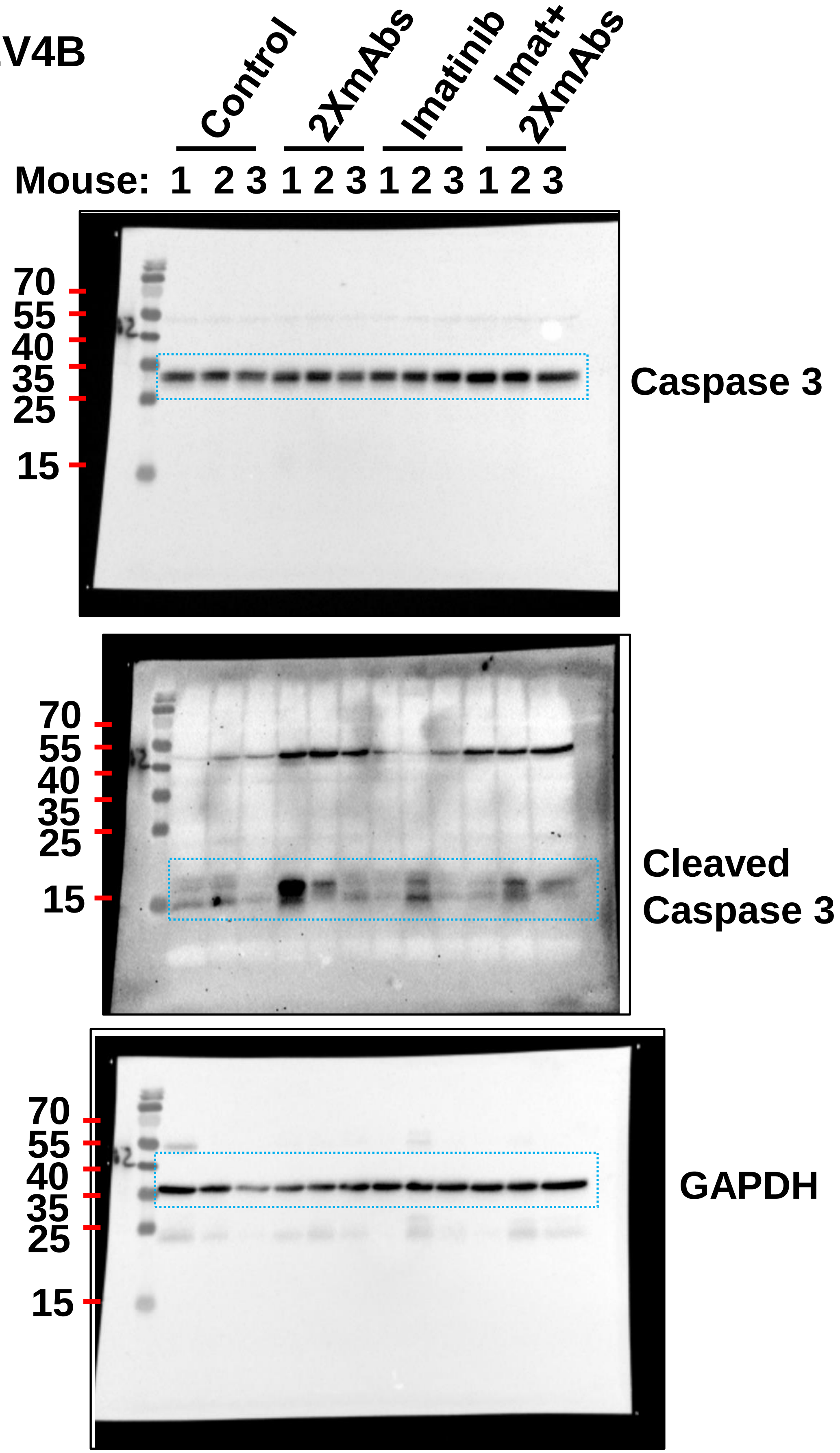
Control
2XmAbs
Imatinib
Imat+ 2XmAbs
Mouse: 1 2 3 1 2 3 1 2 3 1 2 3
70
55
40
35
25
15
Caspase 3
70
55
40
35
25
15
Cleaved Caspase 3
70
55
40
35
25
15
GAPDH

Figure EV4B

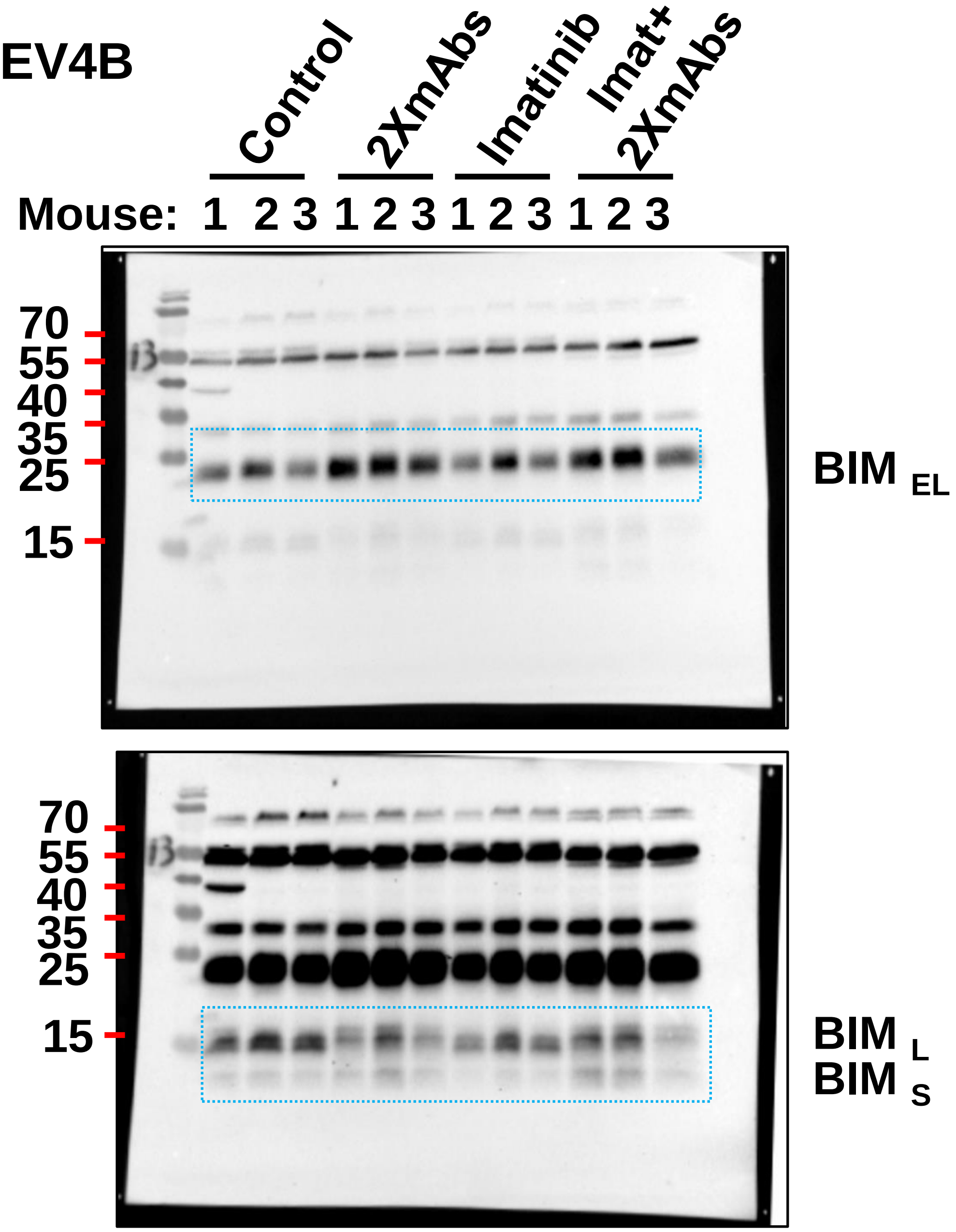
Control
2XmAbs
Imatinib
Imat+ 2XmAbs
Mouse: 1 2 3 1 2 3 1 2 3 1 2 3
70
55
40
35
25
15
BIM EL
70
55
40
35
25
15
BIM L
BIM S

Figure EV4B

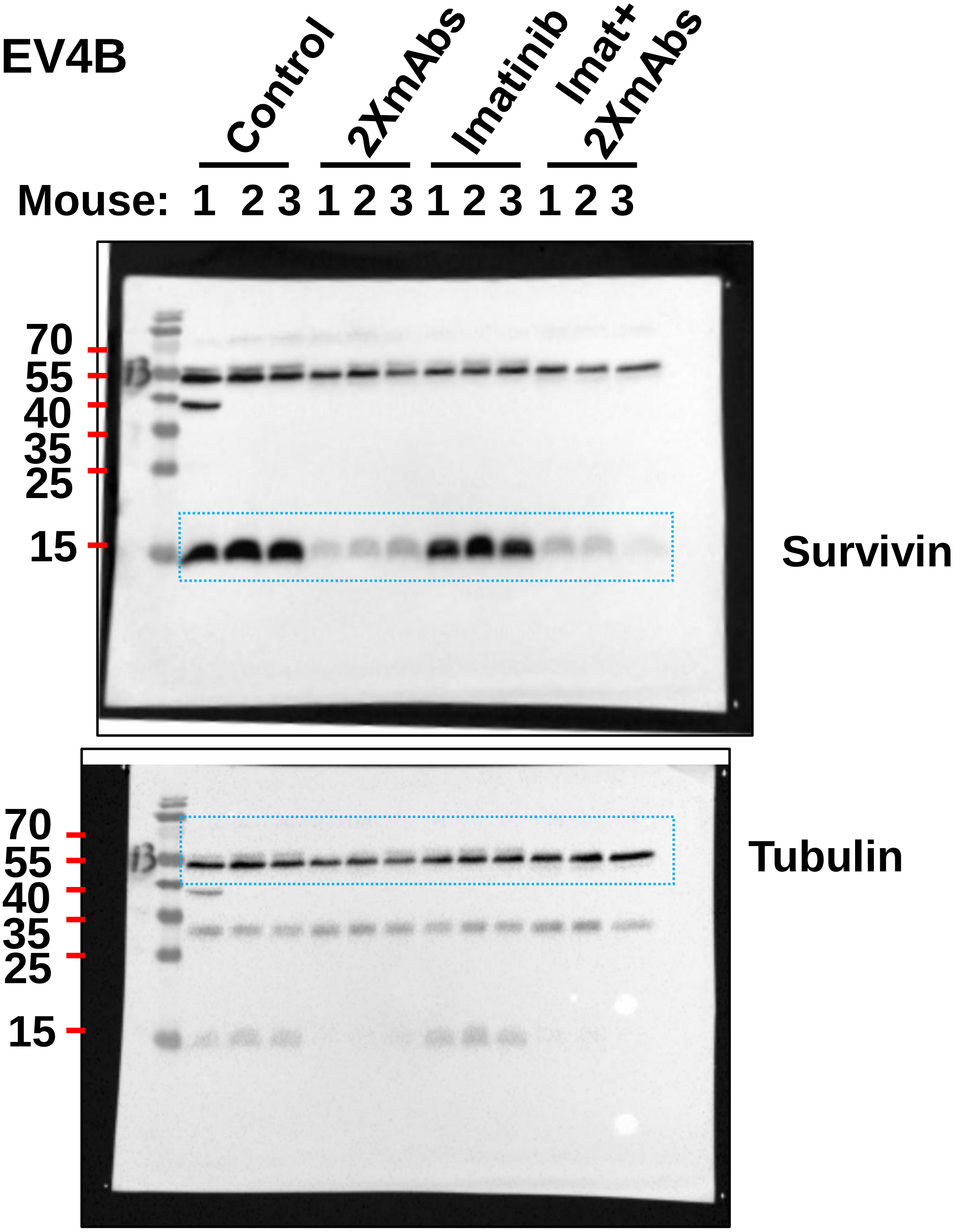

Control
2XmAbs
Imatinib
Imat+ 2XmAbs
Mouse: 1 2 3 1 2 3 1 2 3 1 2 3
70
55
40
35
25
15
Survivin
70
55
40
35
25
15
Tubulin

Figure EV4C

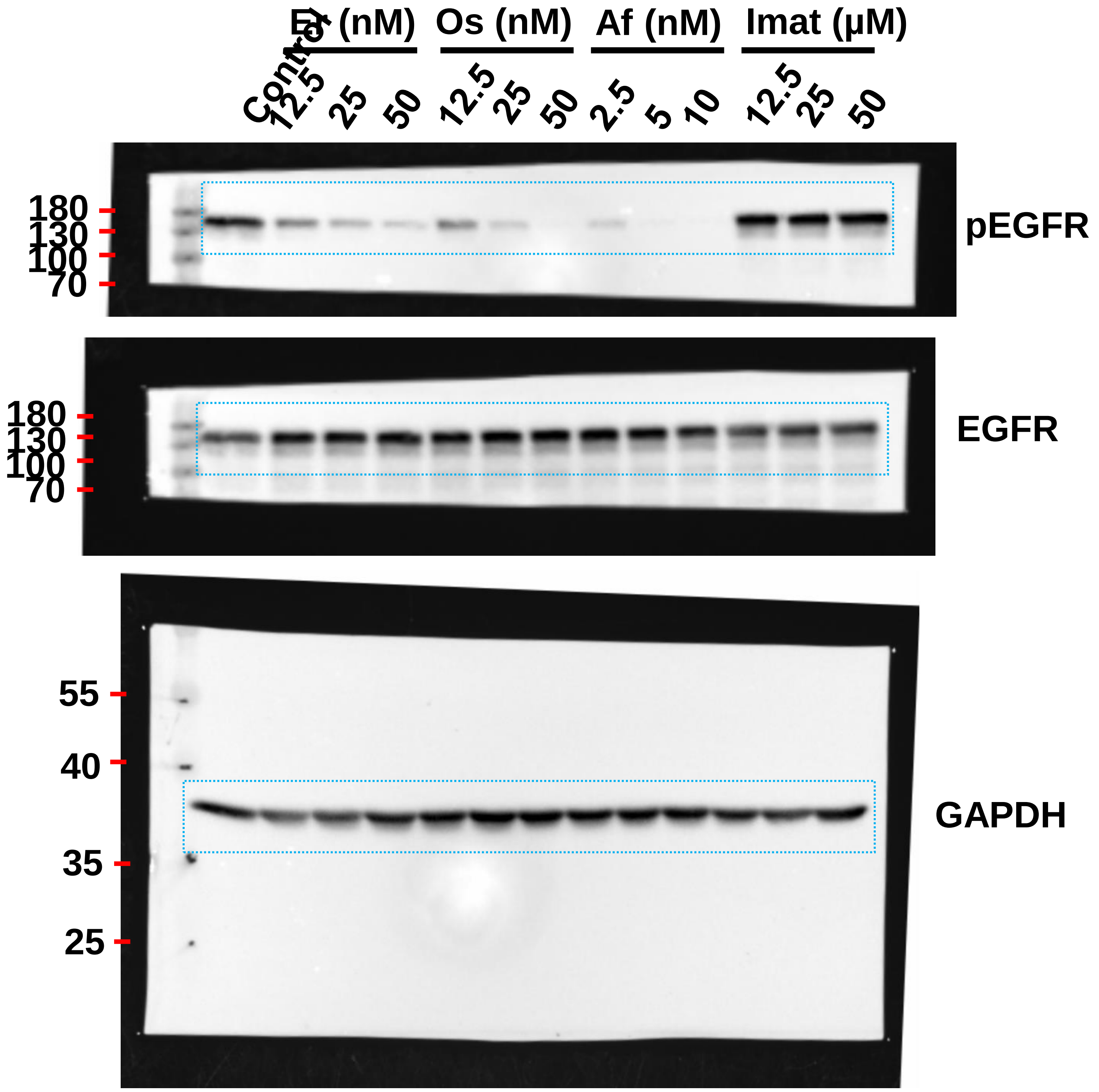
Control
Erl (nM)
Os (nM)
Af (nM)
Imat (µM)
12.5
25
50
12.5
25
50
2.5
5
10
12.5
25
50
180
130
100
70
pEGFR
180
130
100
70
EGFR
55
40
35
25
GAPDH
