# Supplementary material for: Upfront admixing antibodies and EGFR inhibitors preempts sequential treatments in lung cancer models
Source: EMBO Mol Med. 2021 Mar 4;13(4):e13144. doi: 10.15252/emmm.202013144 (PMC8033519; doi:10.15252/emmm.202013144)

Figure EV2B

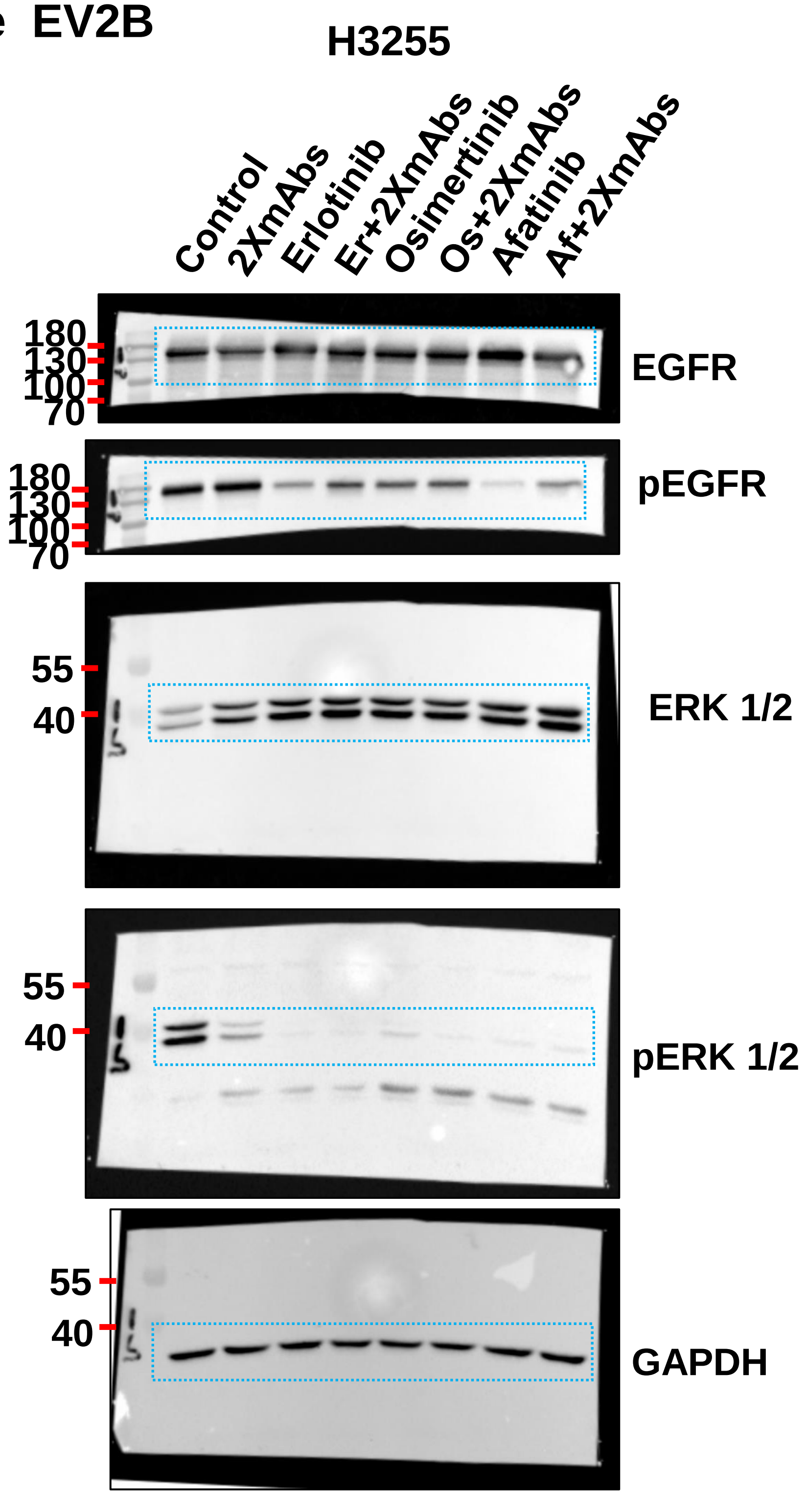
H3255
Control
2XmAbs
Erlotinib
Er+2XmAbs
Osimertinib
Os+2XmAbs
Afatinib
Af+2XmAbs
180
130
100
70
EGFR
180
130
100
70
pEGFR
55
40
ERK 1/2
55
40
pERK 1/2
55
40
GAPDH

Figure EV2B

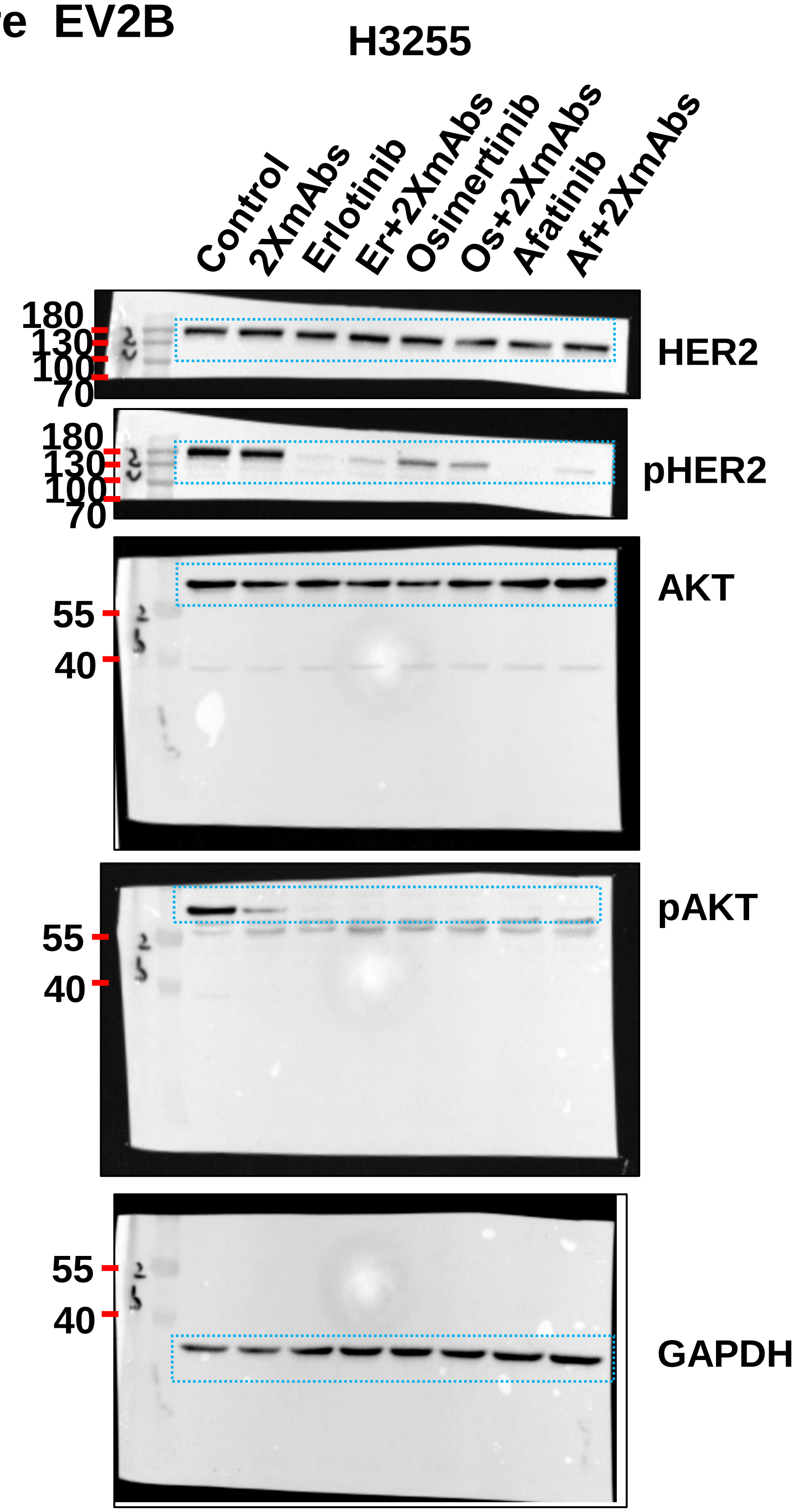

H3255
Control
2XmAbs
Erlotinib
Er+2XmAbs
Osimertinib
Os+2XmAbs
Afatinib
Af+2XmAbs
180
130
100
70
HER2
180
130
100
70
pHER2
55
40
AKT
55
40
pAKT
55
40
GAPDH

Figure EV2B

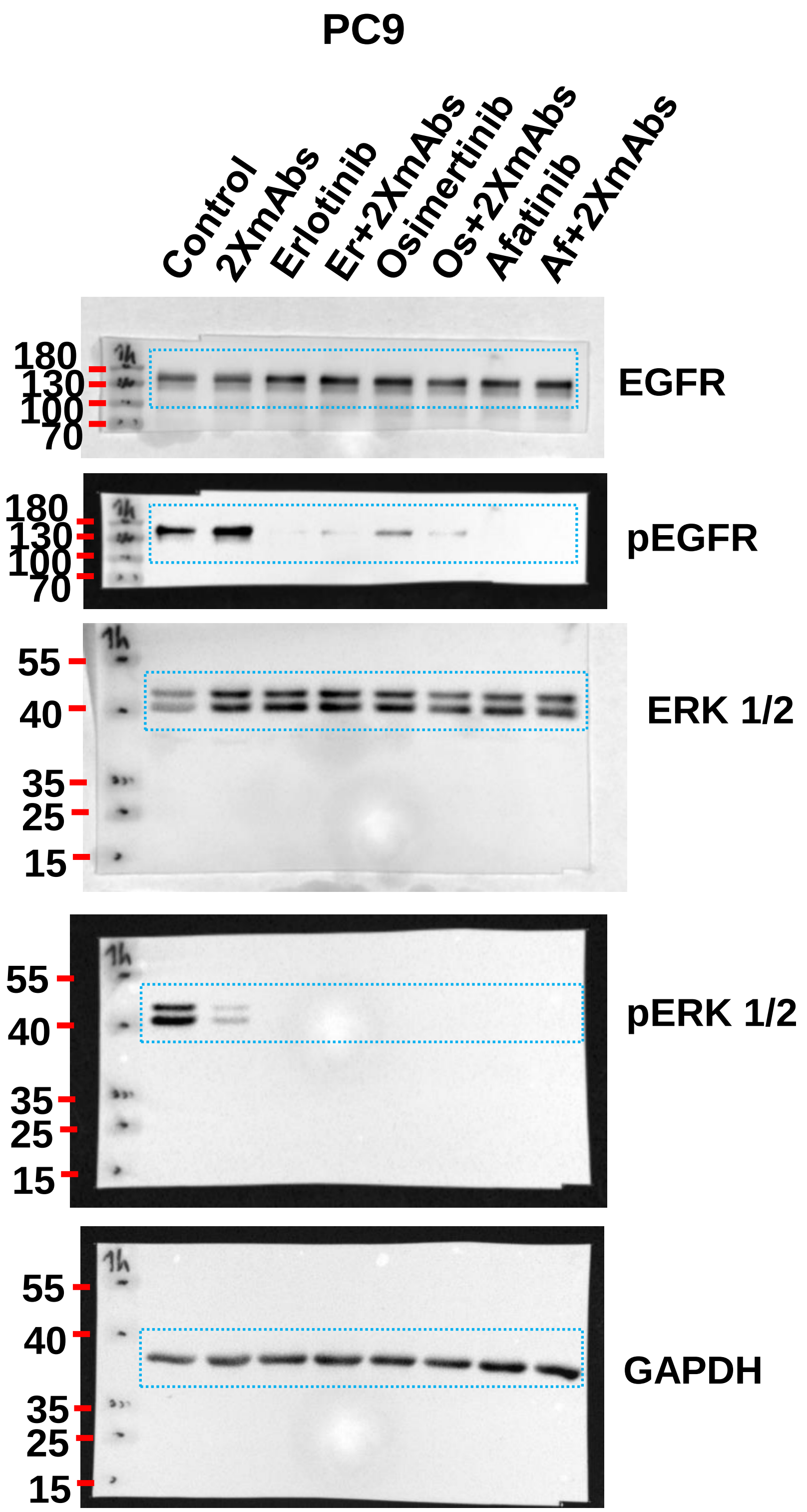

PC9
Control
2XmAbs
Erlotinib
Er+2XmAbs
Osimertinib
Os+2XmAbs
Afatinib
Af+2XmAbs
180
130
100
70
EGFR
180
130
100
70
pEGFR
55
40
35
25
15
ERK 1/2
55
40
35
25
15
pERK 1/2
55
40
35
25
15
GAPDH

Figure EV2B

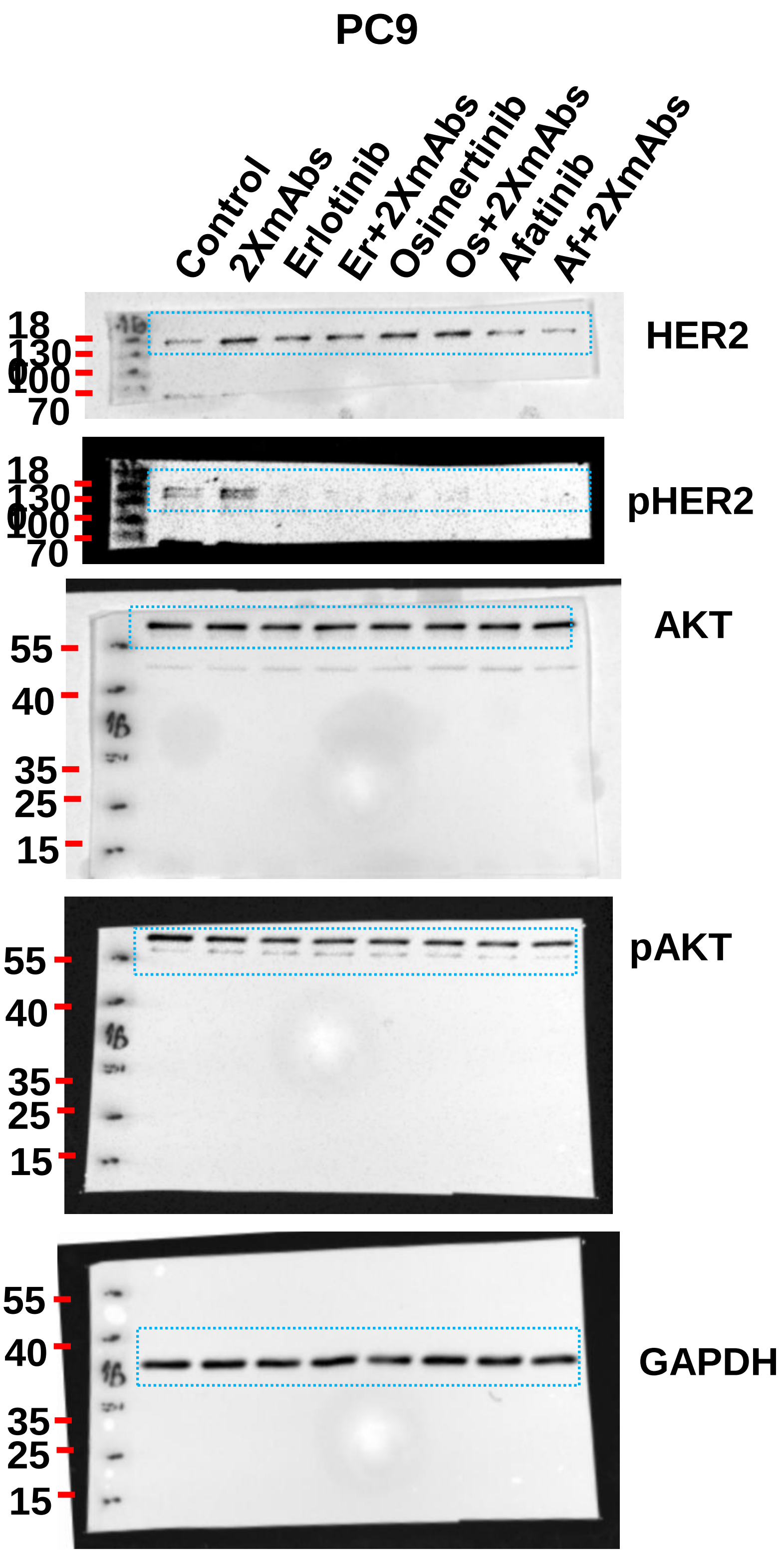
PC9
Control
2XmAbs
Erlotinib
Er+2XmAbs
Osimertinib
Os+2XmAbs
Afatinib
Af+2XmAbs
HER2
pHER2
AKT
pAKT
GAPDH
180
130
100
70
55
40
35
25
15

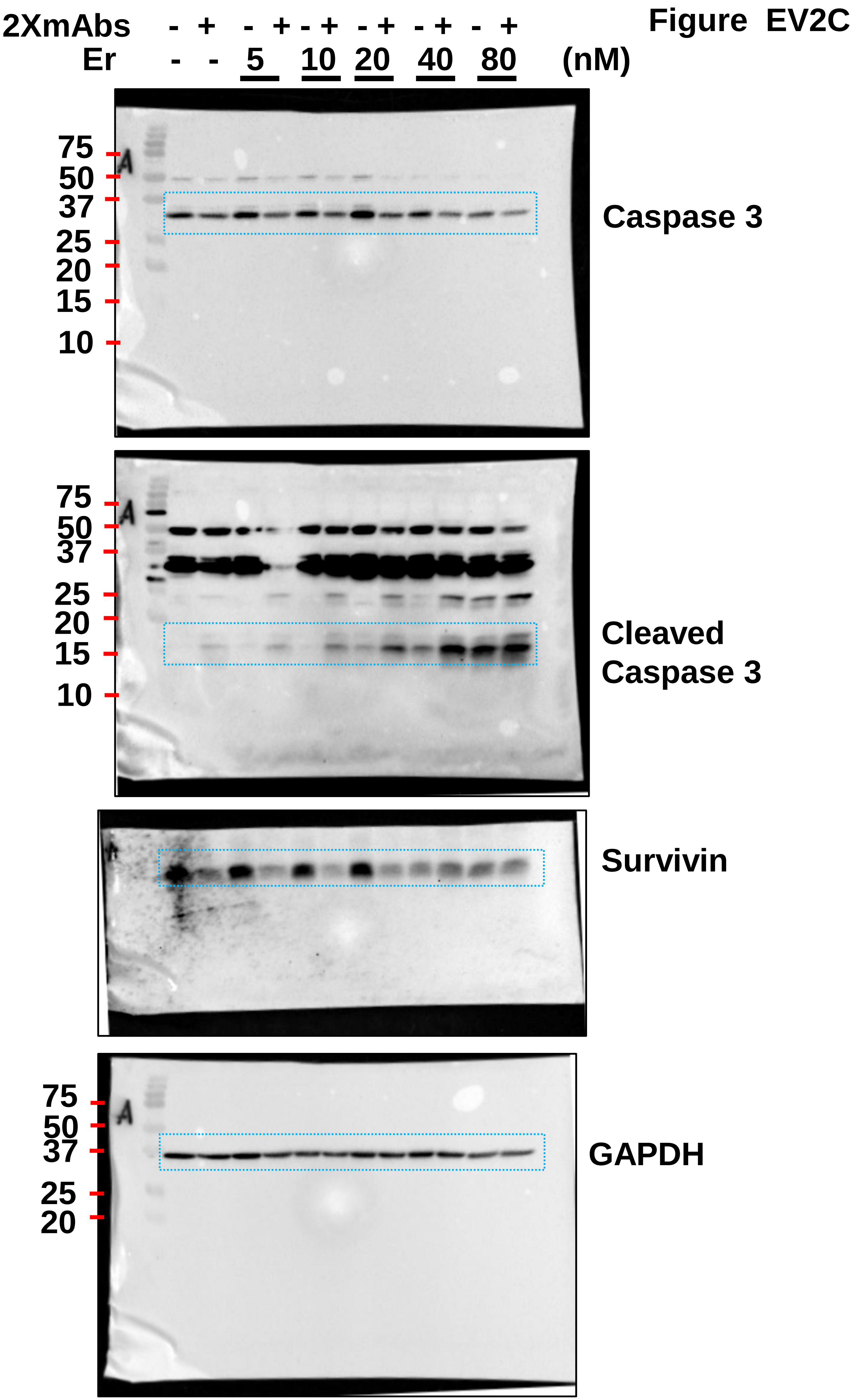
Figure EV2C
2XmAbs - + - + - + - + - + - +
Er - - 5 10 20 40 80 (nM)
75
50
37
25
20
15
10
Caspase 3
Cleaved
Caspase 3
Survivin
GAPDH

Figure EV2C

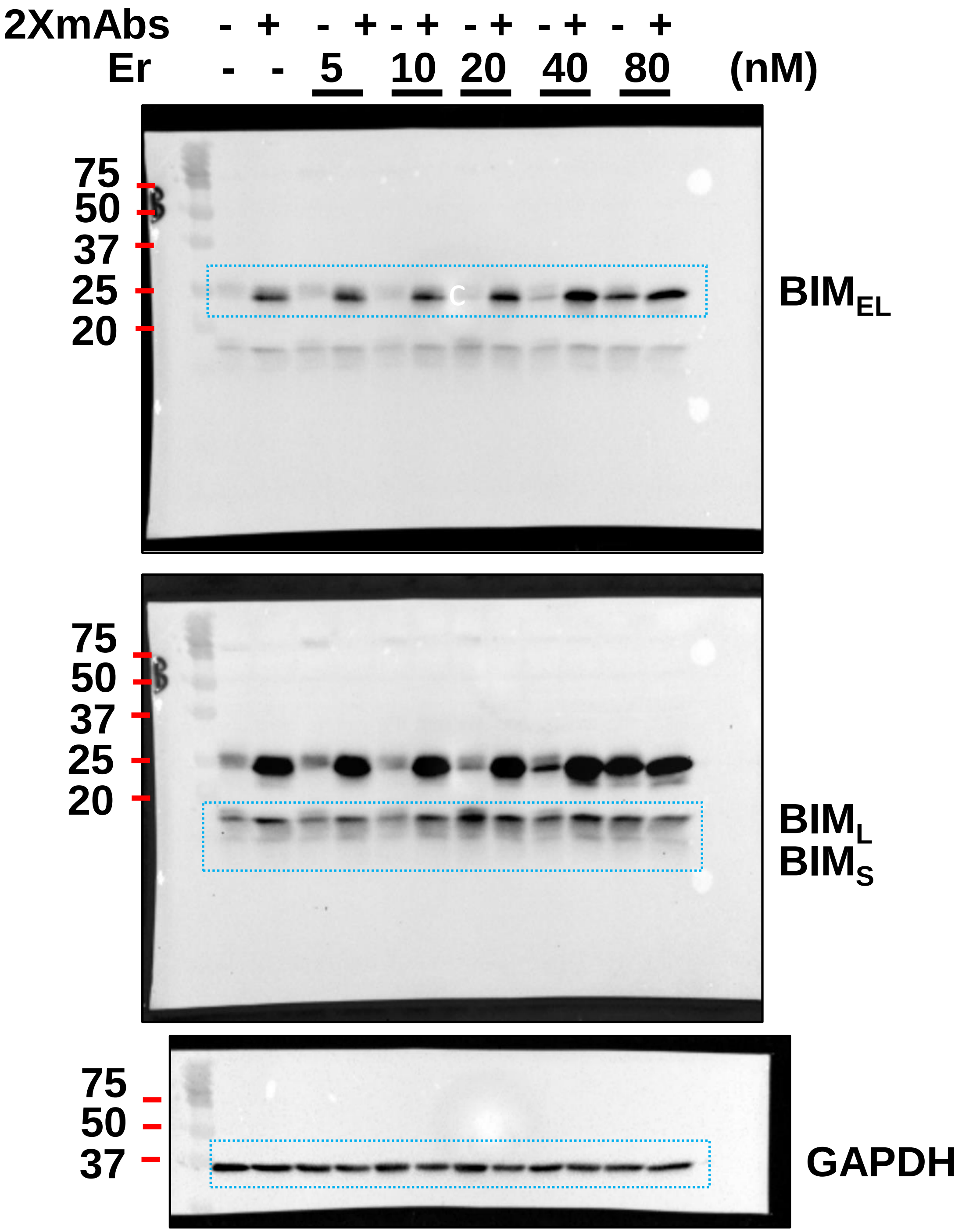

2XmAbs - + - + - + - + - + - +
Er - - 5 10 20 40 80 (nM)
75
50
37
25
20
BIM$_{EL}$
75
50
37
25
20
BIM$_L$
BIM$_S$
75
50
37
GAPDH

Figure EV2C

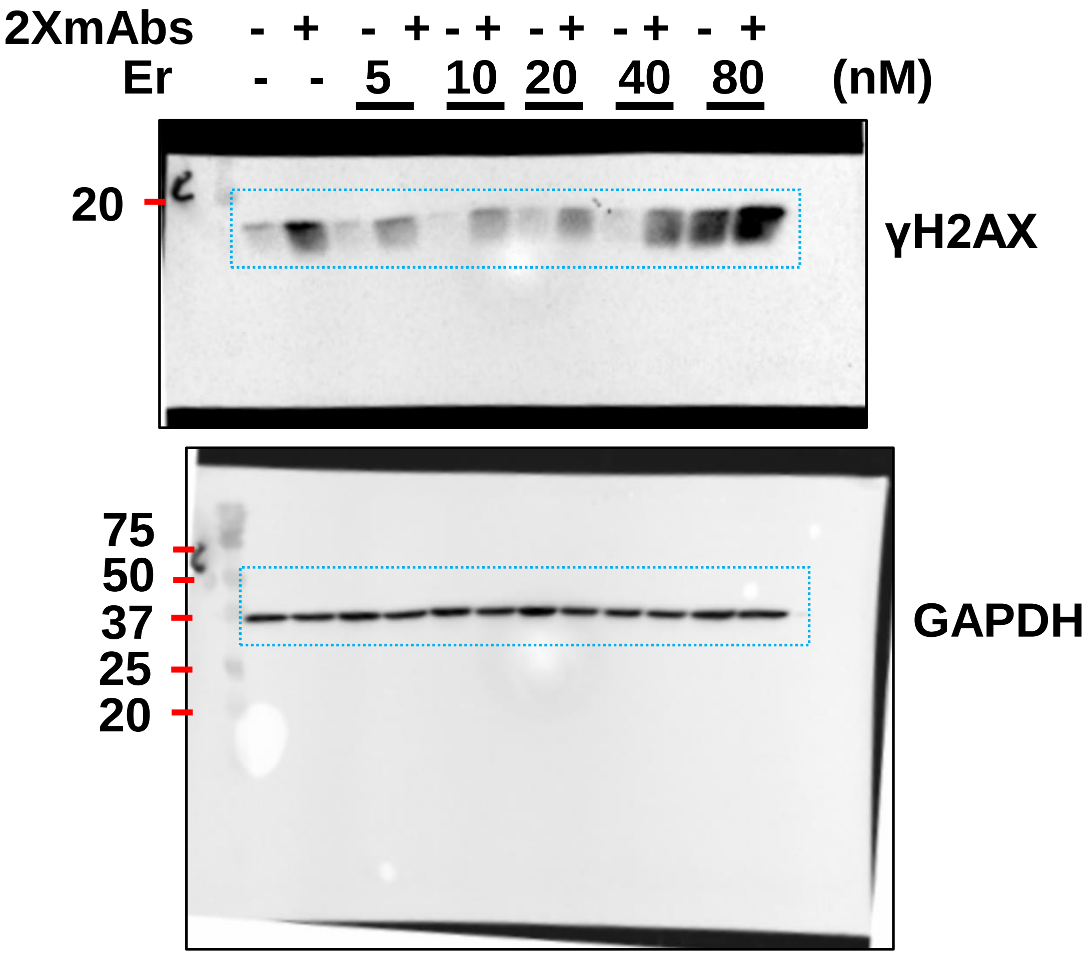

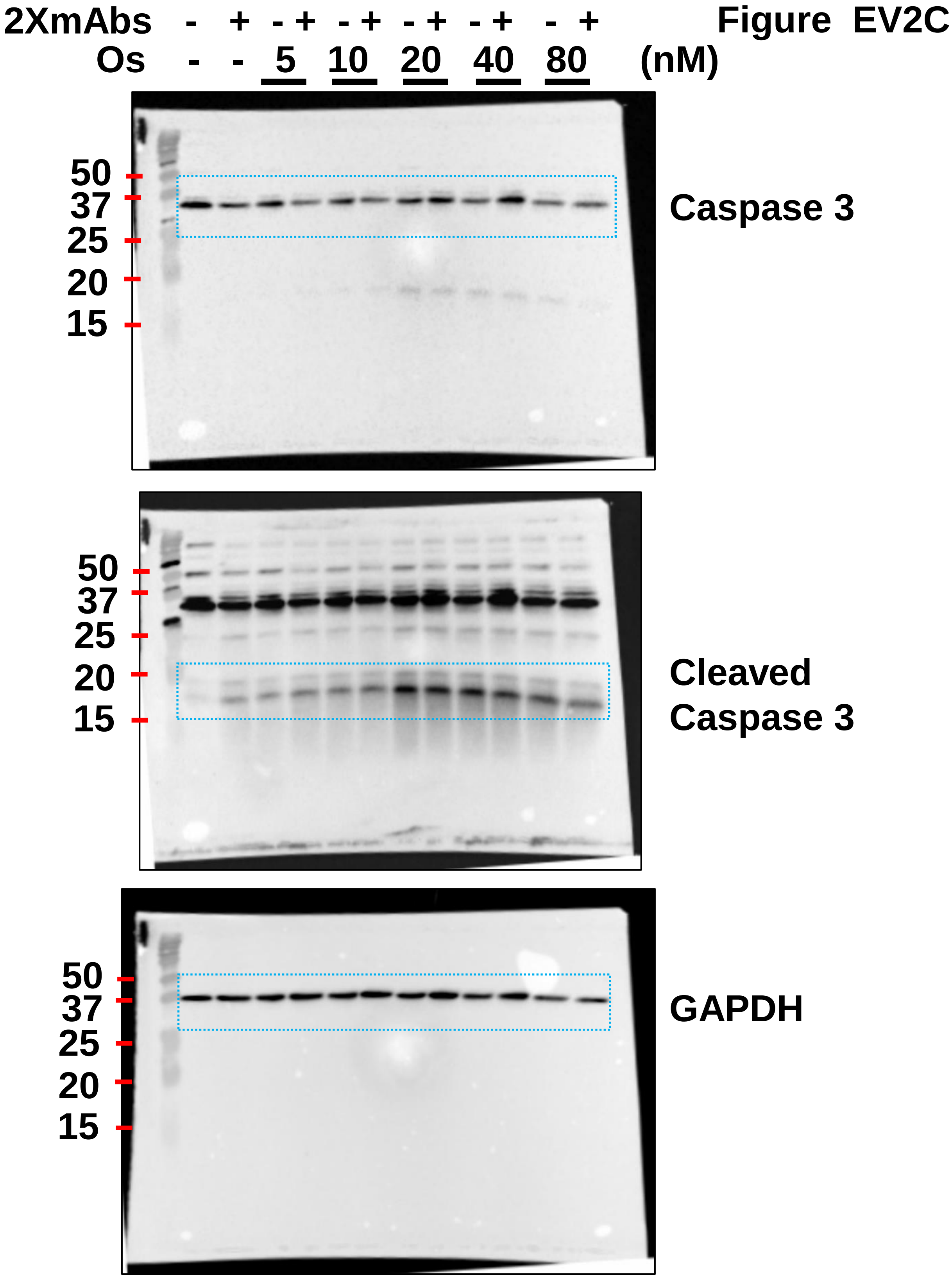
Figure EV2C
2XmAbs - + - + - + - + - + - +
Os - - 5 10 20 40 80 (nM)
50
37
25
20
15
Caspase 3
Cleaved Caspase 3
GAPDH

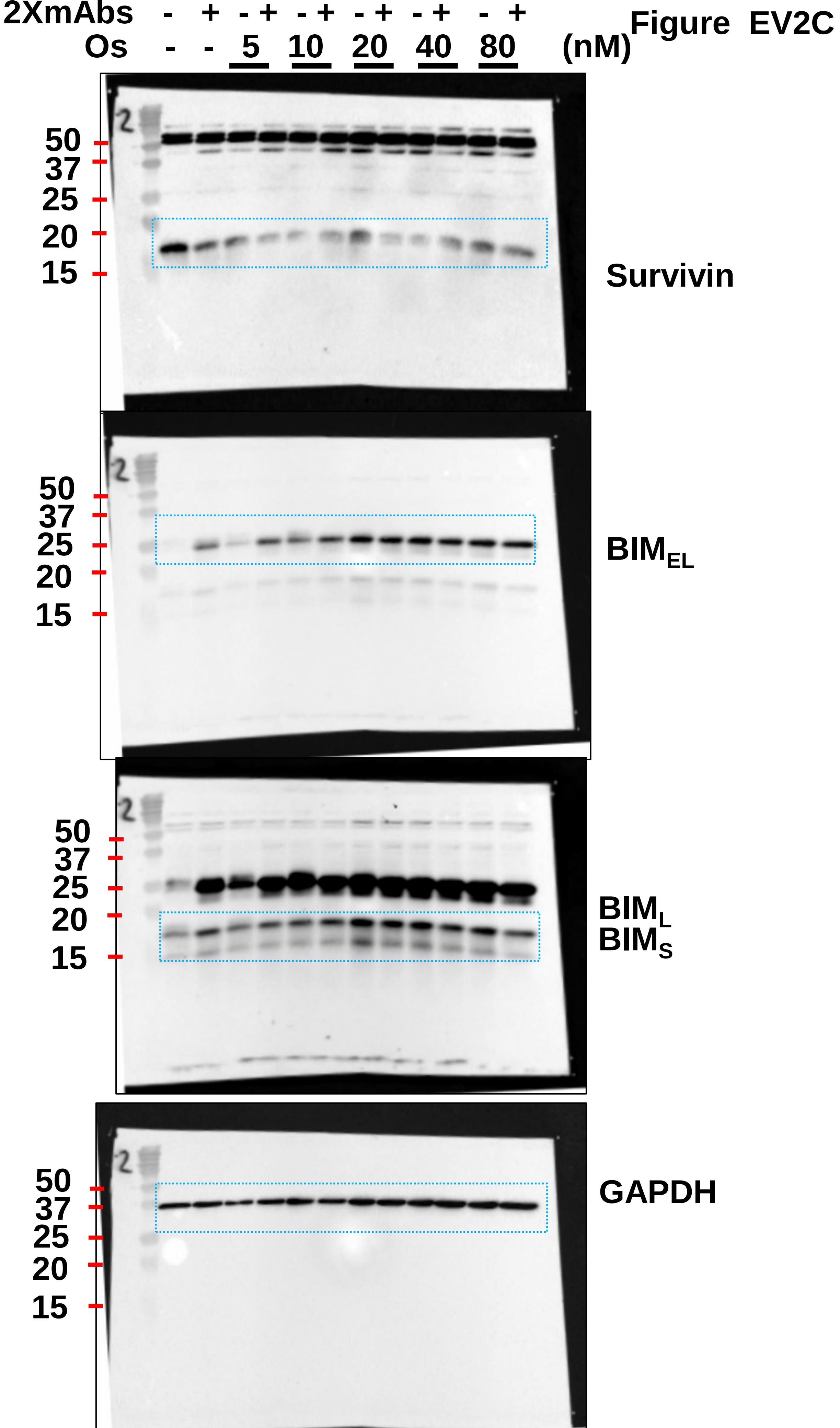
Figure EV2C
2XmAbs - + - + - + - + - + - +
Os - - 5 10 20 40 80 (nM)
50
37
25
20
15
Survivin
BIM_EL
BIM_L
BIM_S
GAPDH

Figure EV2C

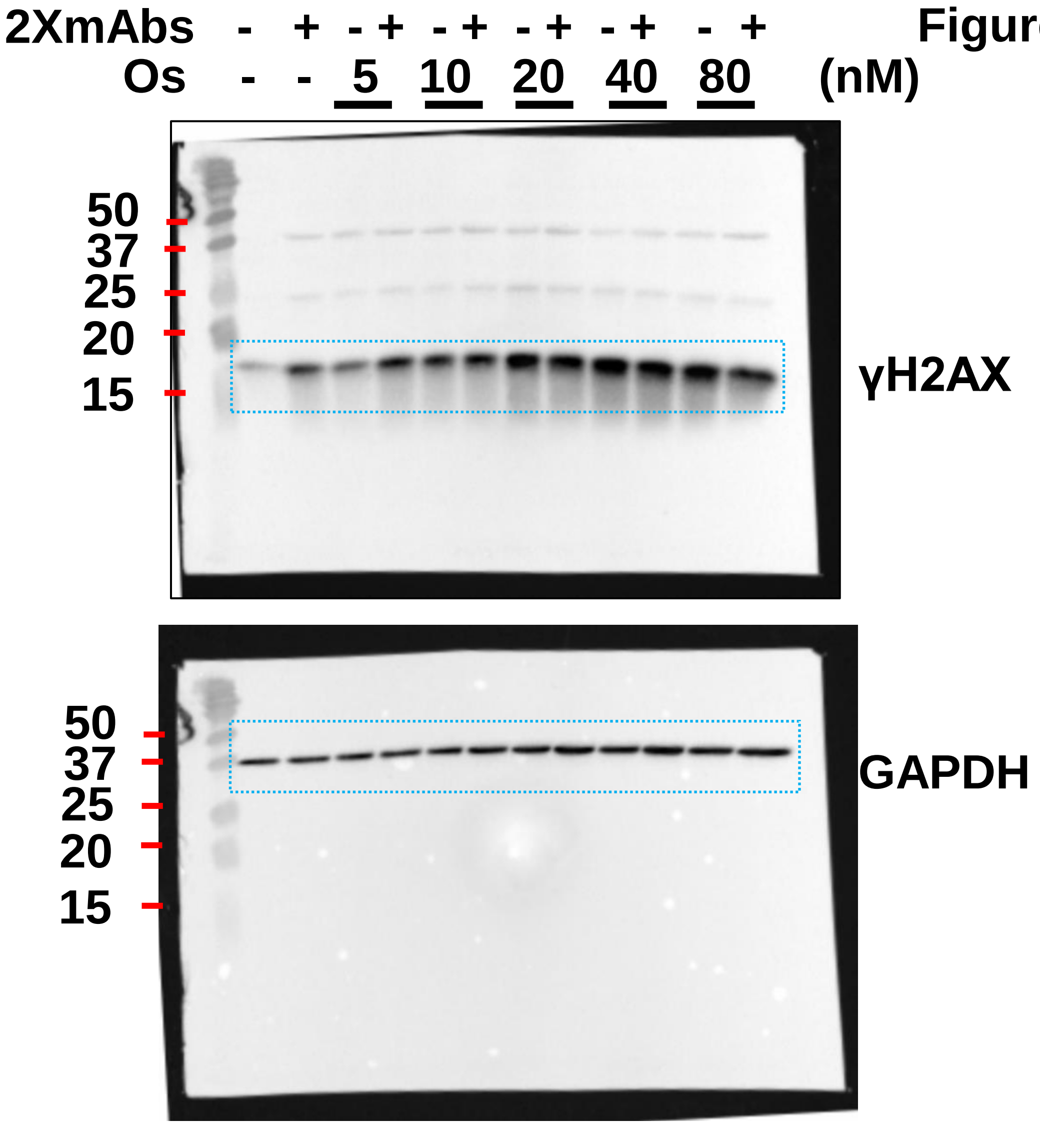

Figure EV2C

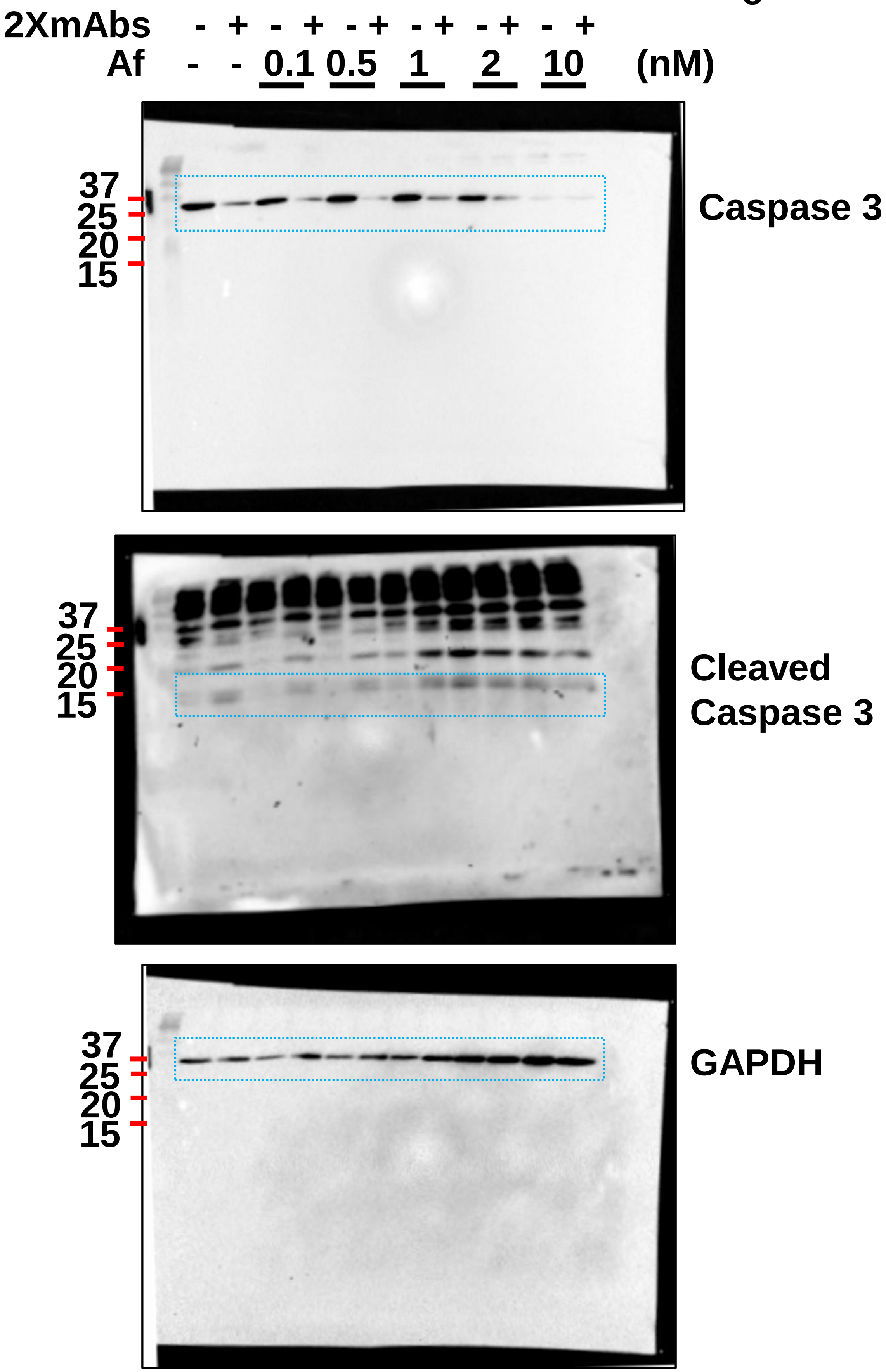
2XmAbs - + - + - + - + - + - +
Af - - 0.1 0.5 1 2 10 (nM)
37
25
20
15
Caspase 3
37
25
20
15
Cleaved Caspase 3
37
25
20
15
GAPDH

Figure EV2C

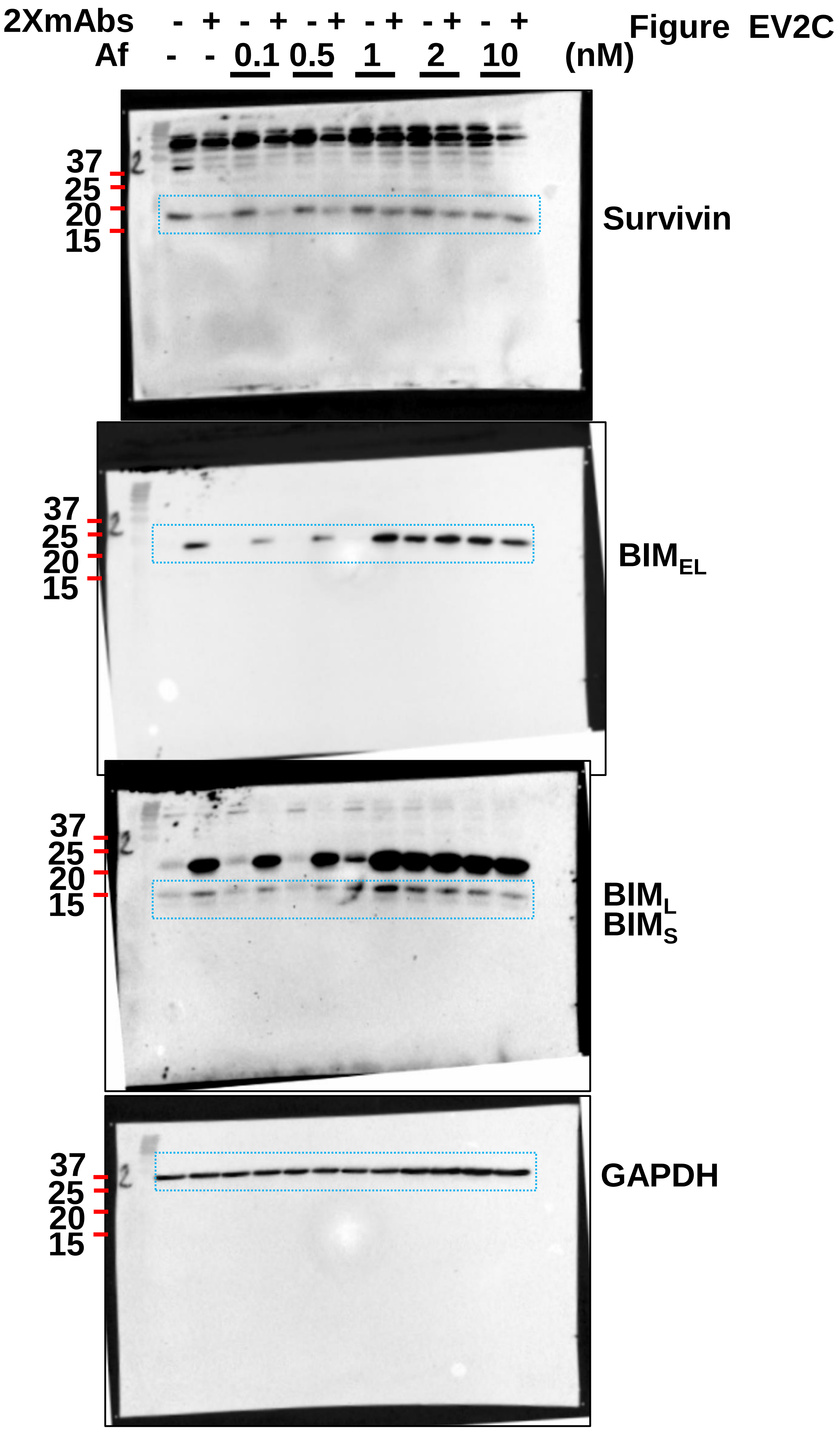
2XmAbs - + - + - + - + - + - +
Af - - 0.1 0.5 1 2 10 (nM)
37
25
20
15
Survivin
BIM$_{EL}$
BIM$_{L}$
BIM$_{S}$
GAPDH

Figure EV2C

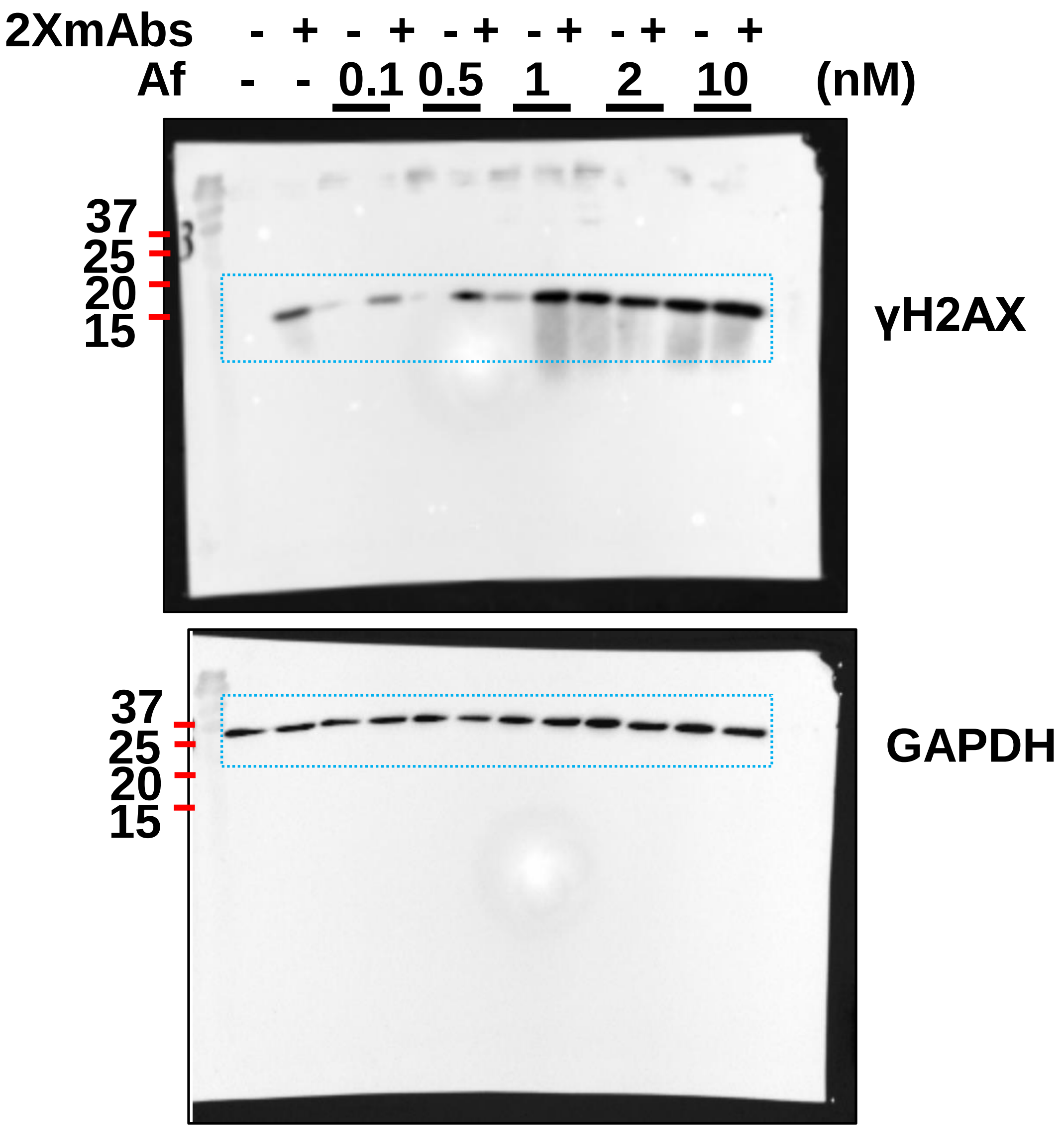

Supplement: Supplementary file 3 — Source Data for Expanded View and Appendix [file EMMM-13-e13144-s003.zip › SourceDataForEVFigures.zip!/SourceDataForFigureEV2B_C.pdf]
