# Supplementary material for: Upfront admixing antibodies and EGFR inhibitors preempts sequential treatments in lung cancer models
Source: EMBO Mol Med. 2021 Mar 4;13(4):e13144. doi: 10.15252/emmm.202013144 (PMC8033519; doi:10.15252/emmm.202013144)
Supplement: Supplementary file 3 — Source Data for Expanded View and Appendix [file EMMM-13-e13144-s003.zip › SourceDataForEVFigures.zip!/SourceDataForFigureEV1B.pdf]

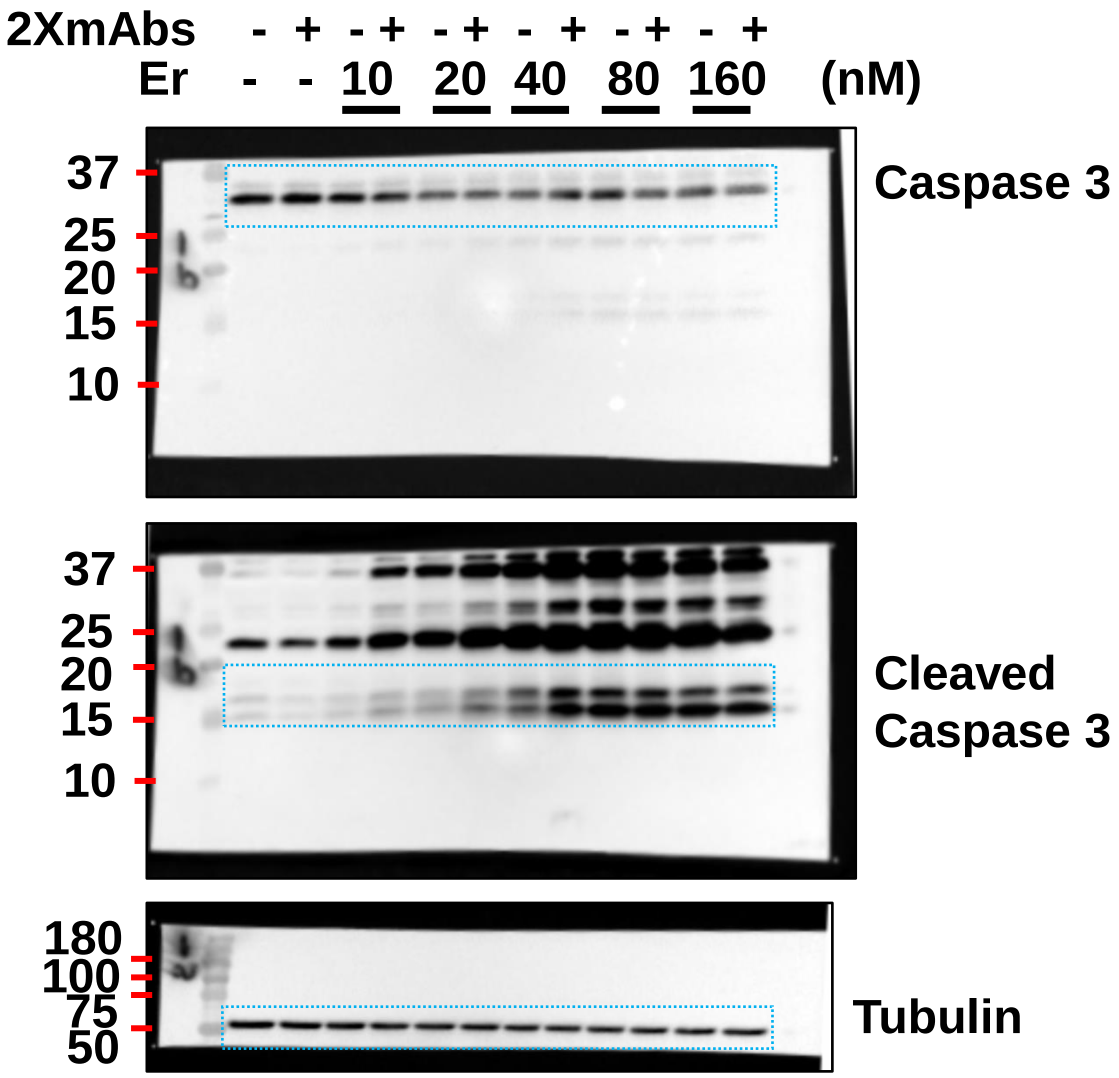
2XmAbs - + - + - + - + - + - +
Er - - 10 20 40 80 160 (nM)
37
25
20
15
10
Caspase 3
37
25
20
15
10
Cleaved
Caspase 3
180
100
75
50
Tubulin

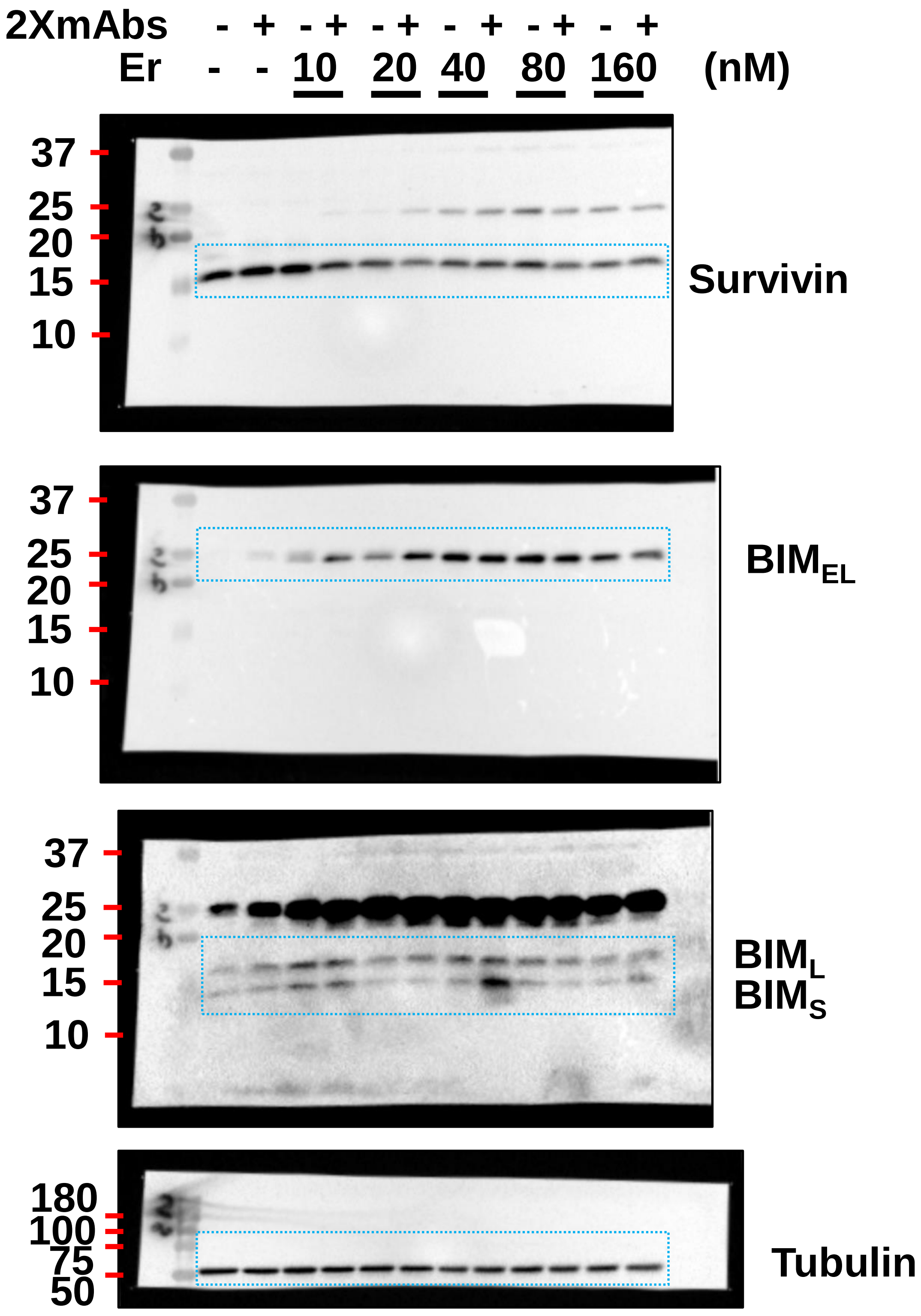

2XmAbs - + - + - + - + - + - +
Er - - 10 20 40 80 160 (nM)
37
25
20
15
10
Survivin
BIMEL
BIML
BIMS
180
100
75
50
Tubulin

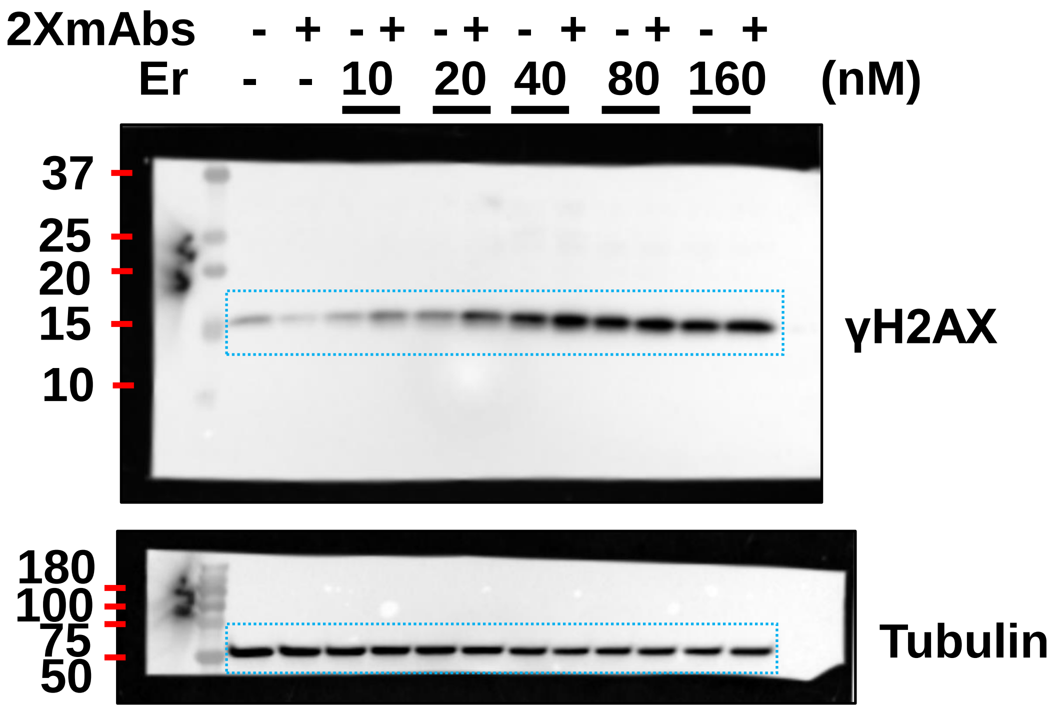
2XmAbs - + - + - + - + - + - +
Er - - 10 20 40 80 160 (nM)
37
25
20
15
10
γH2AX
180
100
75
50
Tubulin

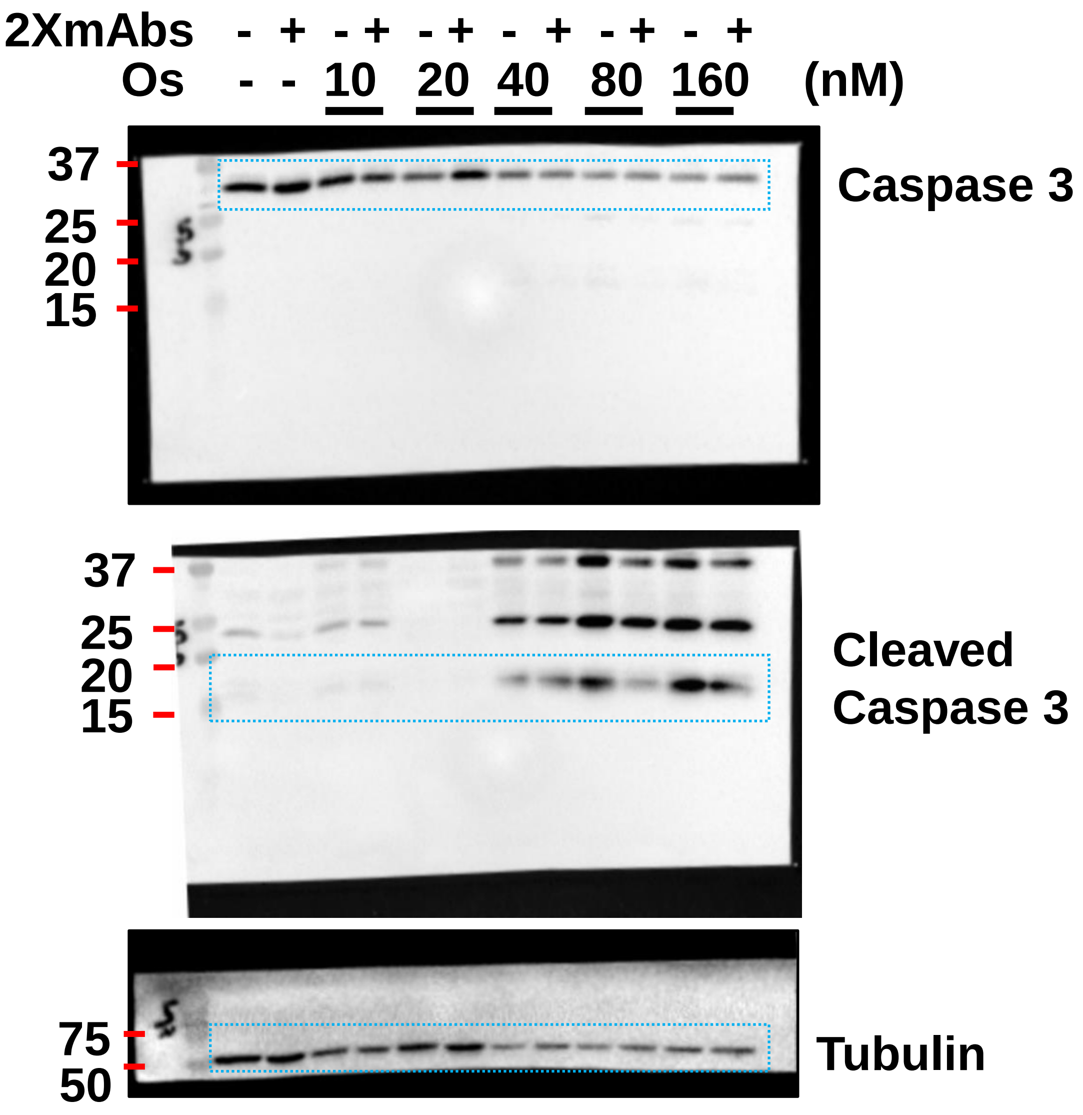
2XmAbs - + - + - + - + - + - +
Os - - 10 20 40 80 160 (nM)
37
25
20
15
Caspase 3
37
25
20
15
Cleaved Caspase 3
75
50
Tubulin

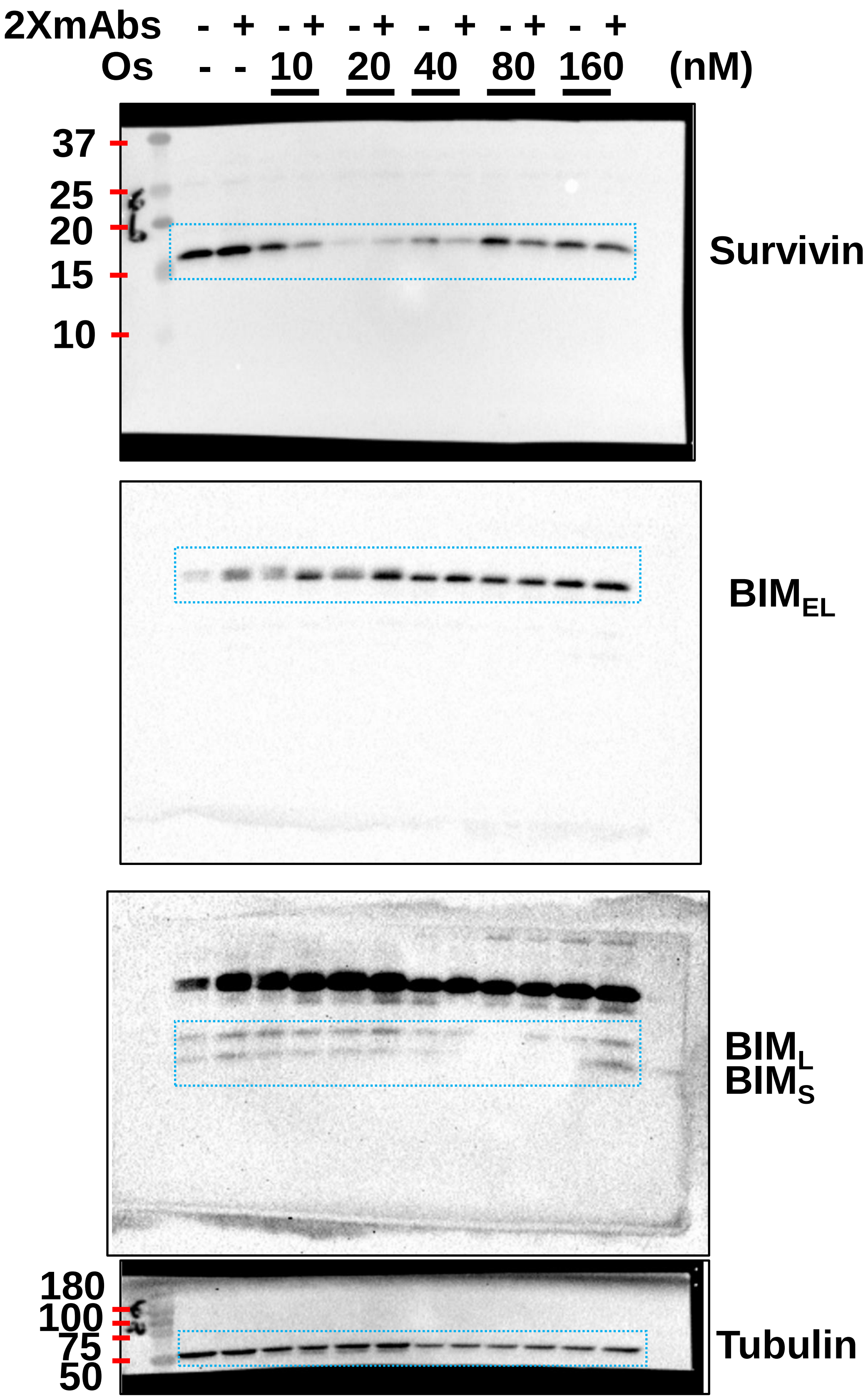
2XmAbs - + - + - + - + - + - +
Os - - 10 20 40 80 160 (nM)
37
25
20
15
10
Survivin
BIM$_{EL}$
BIM$_L$
BIM$_S$
180
100
75
50
Tubulin

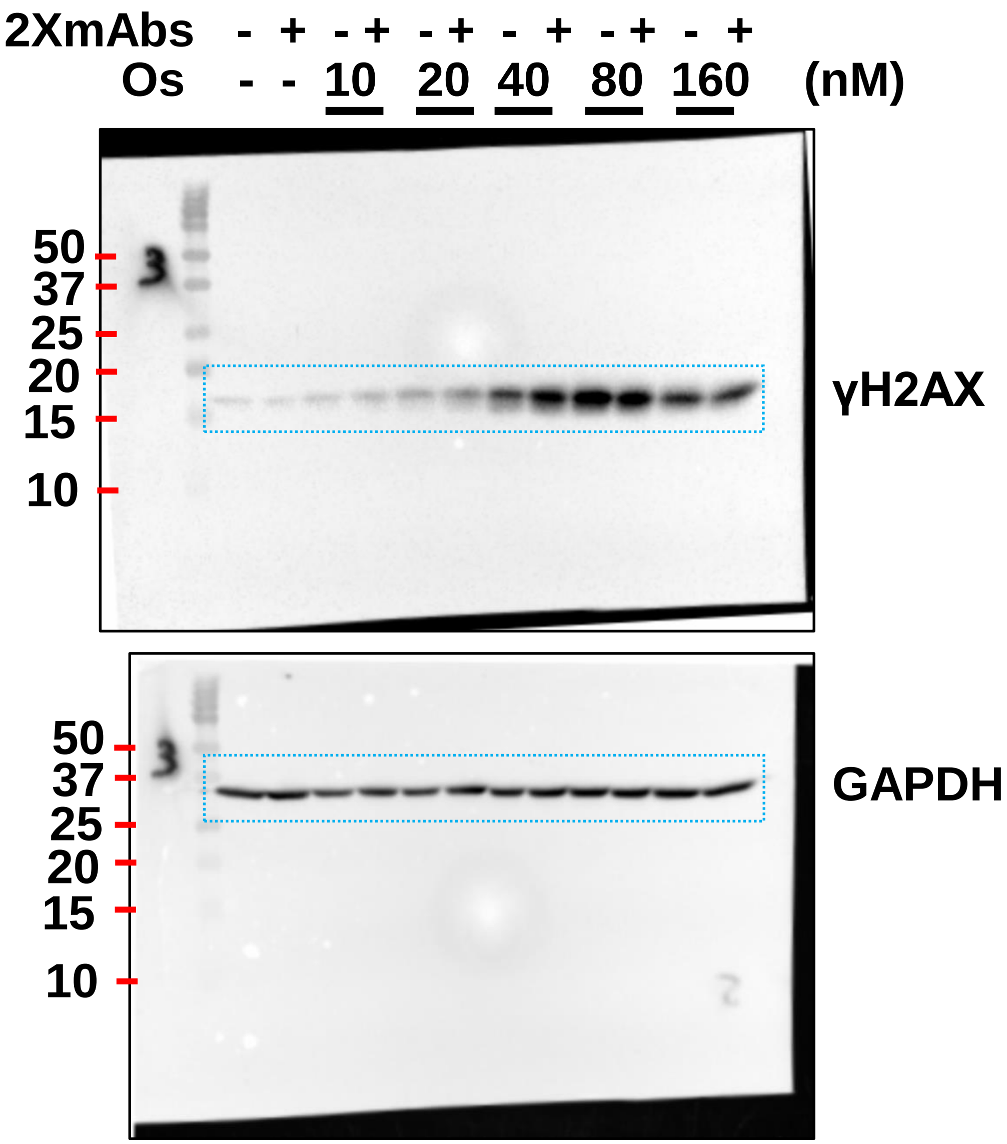

2XmAbs - + - + - + - + - + - +
Os - - 10 20 40 80 160 (nM)
50
37
25
20
15
10
γH2AX
50
37
25
20
15
10
GAPDH

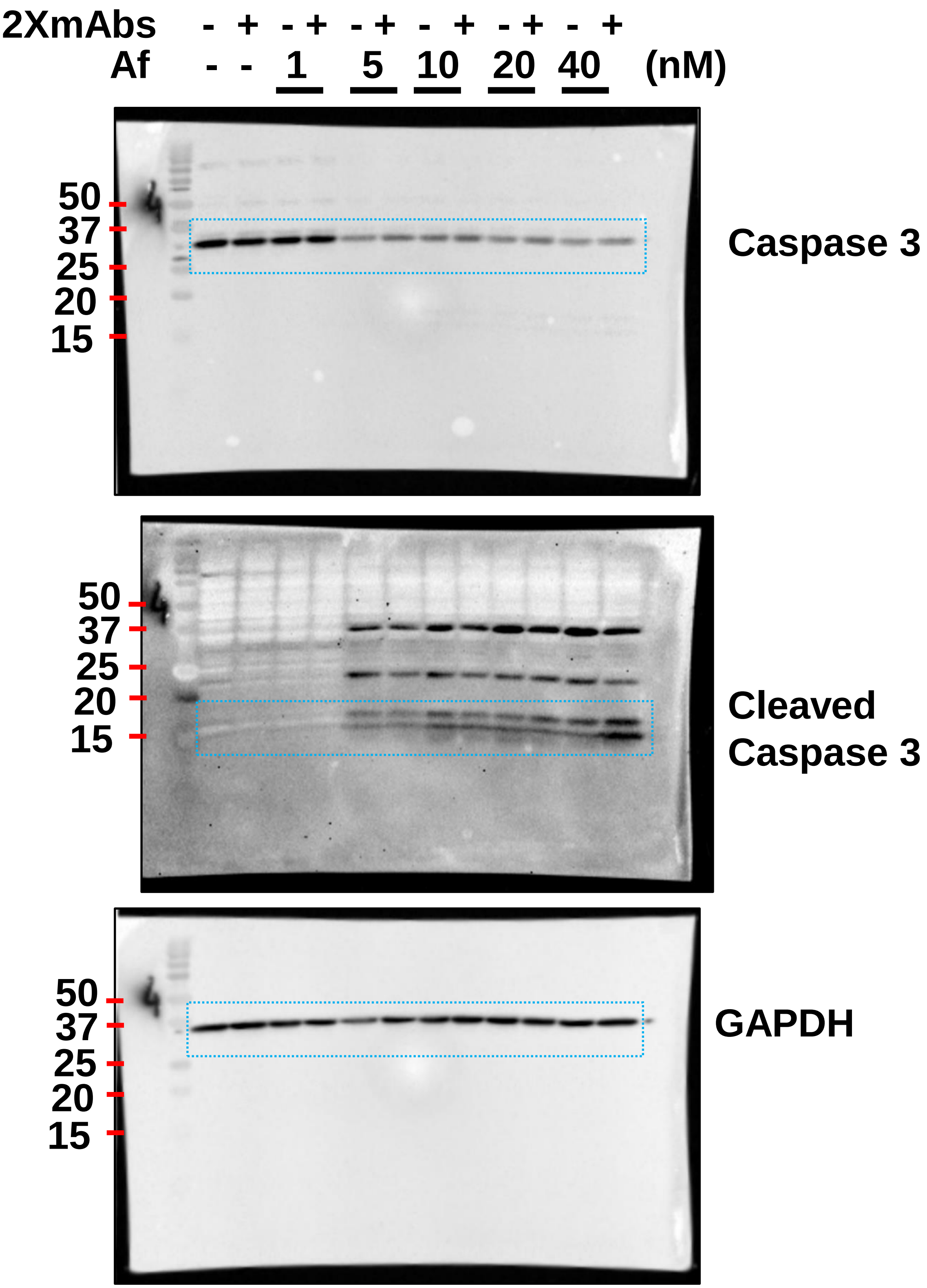
2XmAbs - + - + - + - + - + - +
Af - - 1 5 10 20 40 (nM)
50
37
25
20
15
Caspase 3
Cleaved Caspase 3
GAPDH

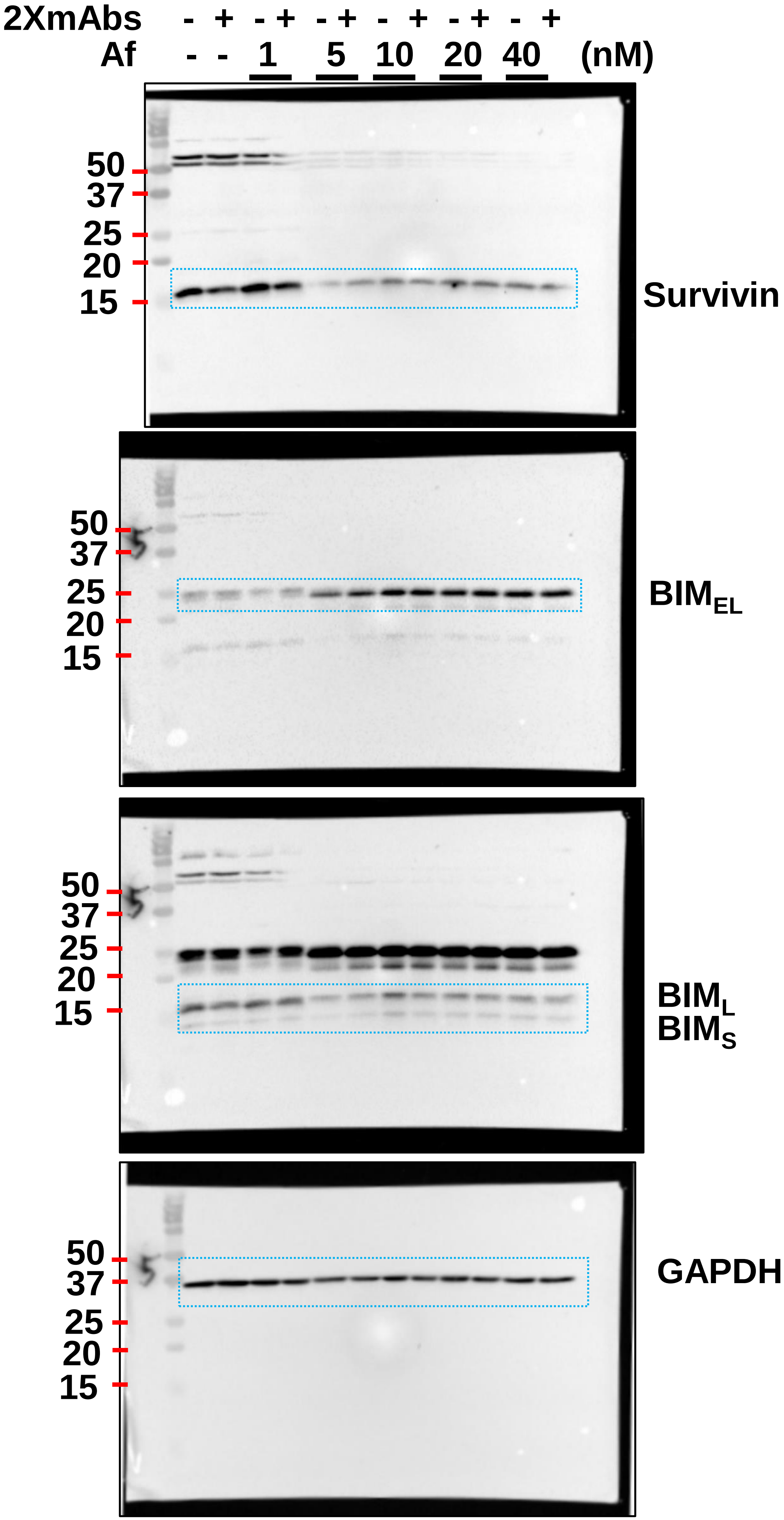

2XmAbs - + - + - + - + - + - +
Af - - 1 5 10 20 40 (nM)
50
37
25
20
15
Survivin
BIM$_{EL}$
BIM$_L$
BIM$_S$
GAPDH

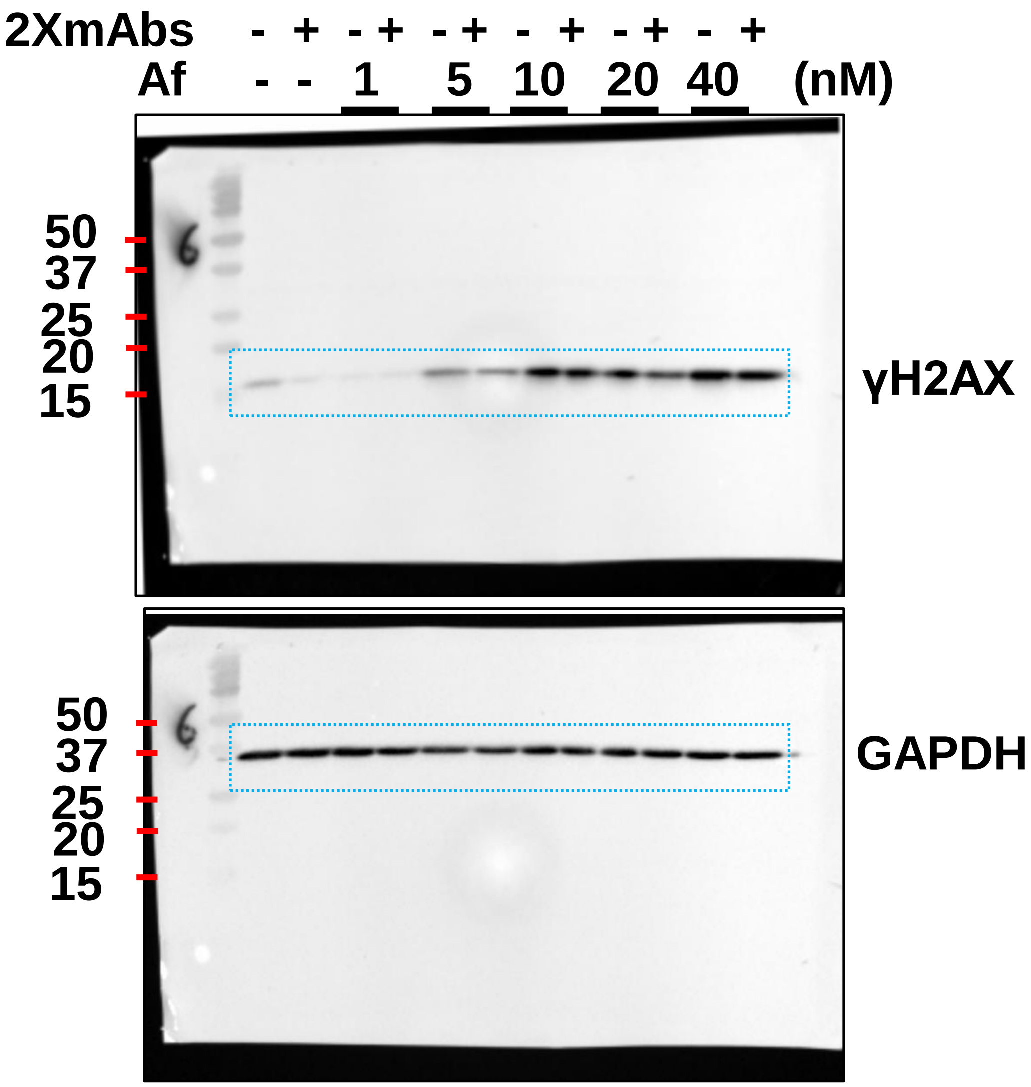
2XmAbs - + - + - + - + - + - +
Af - - 1 5 10 20 40 (nM)
50
37
25
20
15
γH2AX
50
37
25
20
15
GAPDH
