# Supplementary material for: Upfront admixing antibodies and EGFR inhibitors preempts sequential treatments in lung cancer models
Source: EMBO Mol Med. 2021 Mar 4;13(4):e13144. doi: 10.15252/emmm.202013144 (PMC8033519; doi:10.15252/emmm.202013144)
Supplement: Supplementary file 3 — Source Data for Expanded View and Appendix [file EMMM-13-e13144-s003.zip › SourceDataForEVFigures.zip!/SourceDataForFigureEV5D_E.pdf]

Figure EV5D

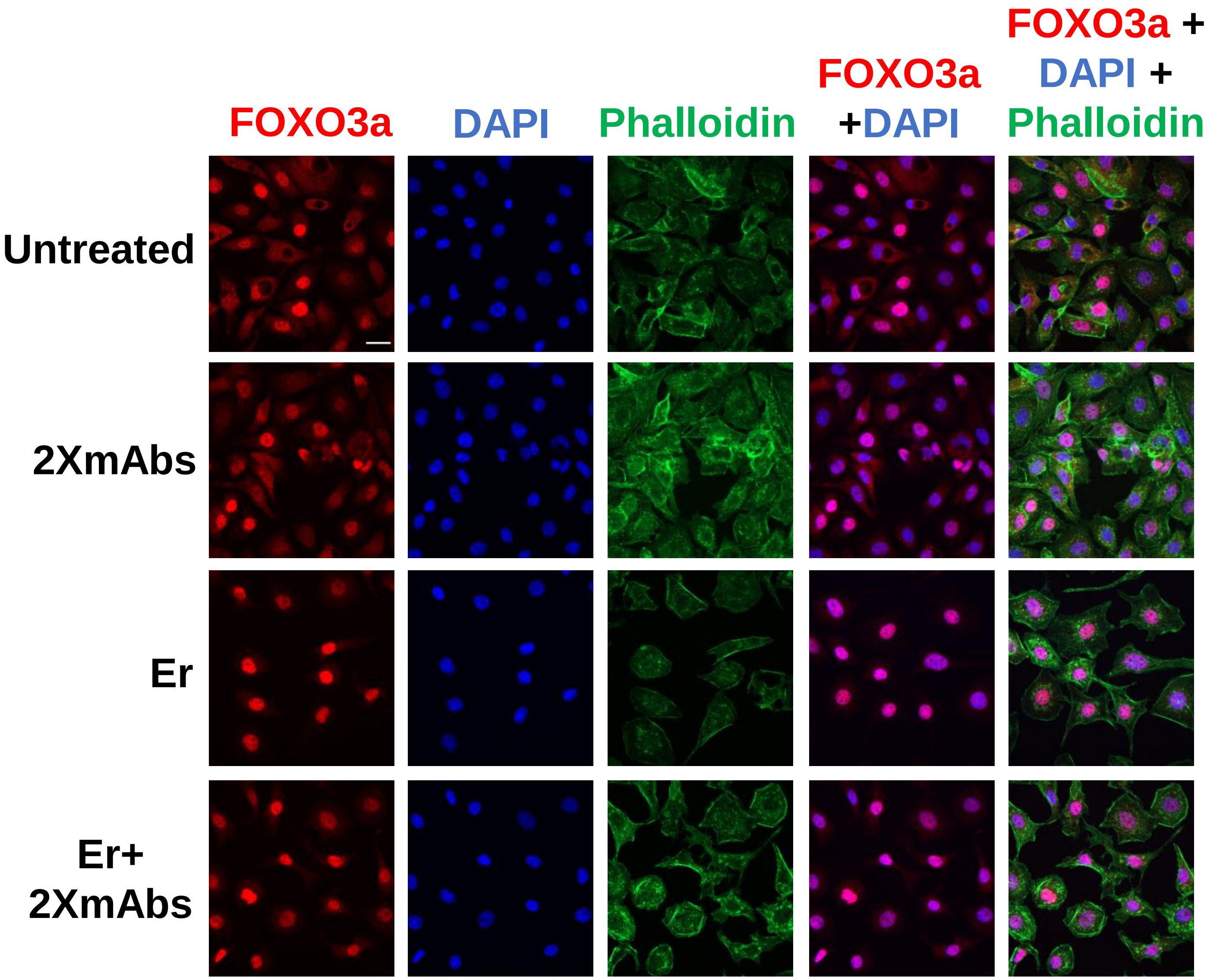

FOXO3a
DAPI
Phalloidin
FOXO3a +DAPI
FOXO3a + DAPI + Phalloidin
Untreated
2XmAbs
Er
Er+ 2XmAbs

Figure EV5E

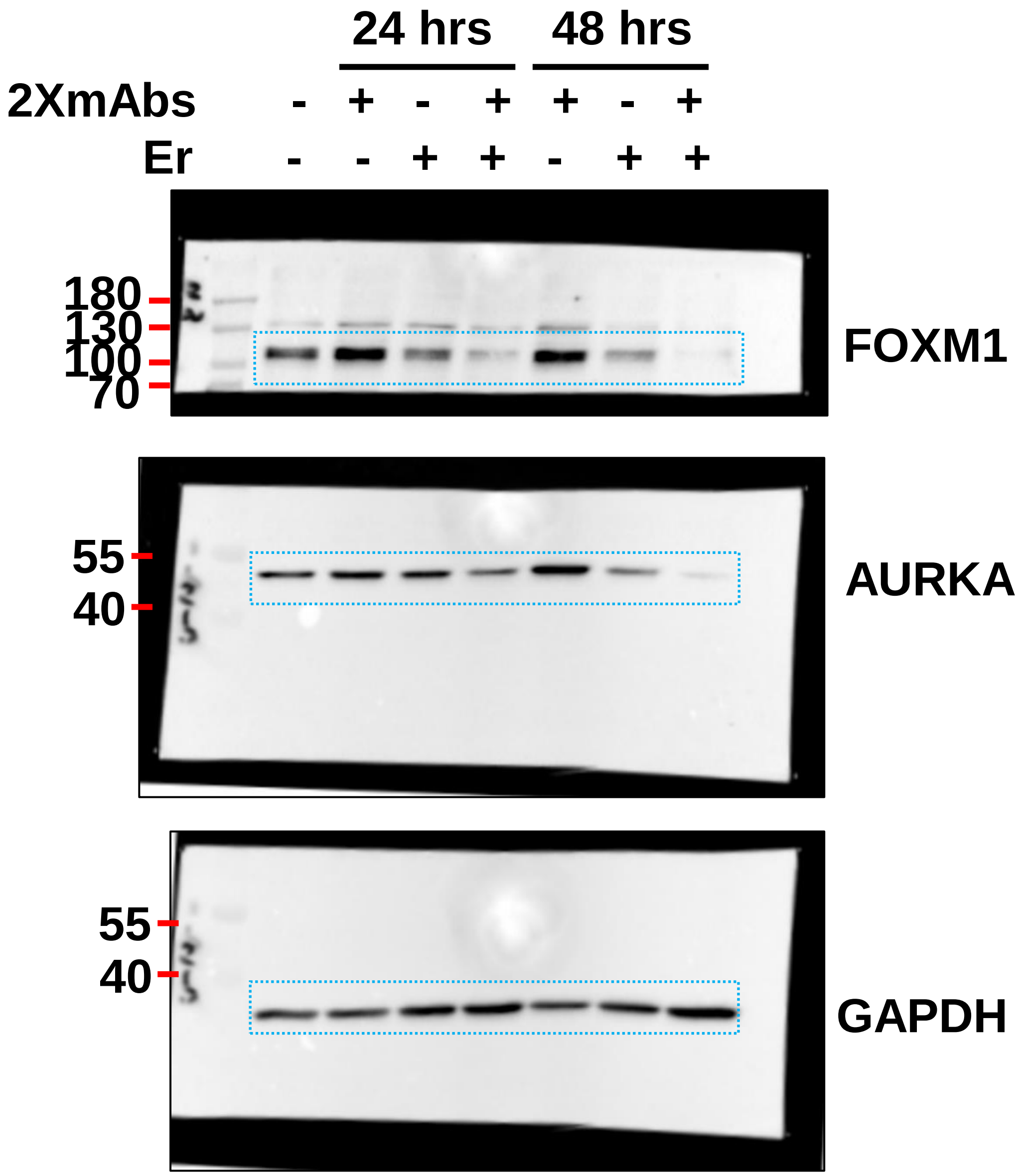

24 hrs
48 hrs
2XmAbs
- + - + + - +
Er
- - + + - + +
180
130
100
70
FOXM1
55
40
AURKA
55
40
GAPDH

Figure EV5E

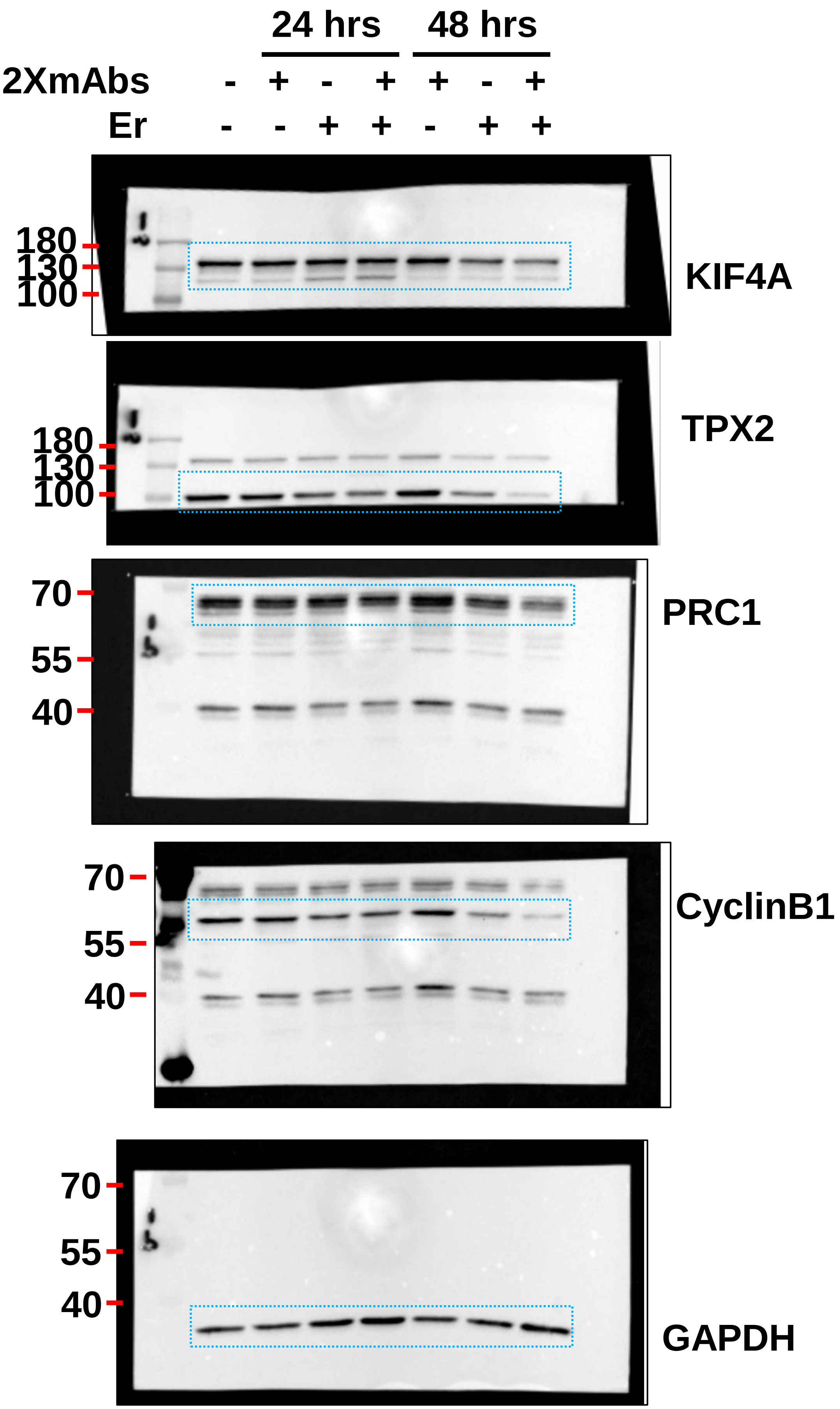

24 hrs
48 hrs
2XmAbs - + - + + - +
Er - - + + - + +
180
130
100
KIF4A
180
130
100
TPX2
70
55
40
PRC1
70
55
40
CyclinB1
70
55
40
GAPDH

Figure EV5E

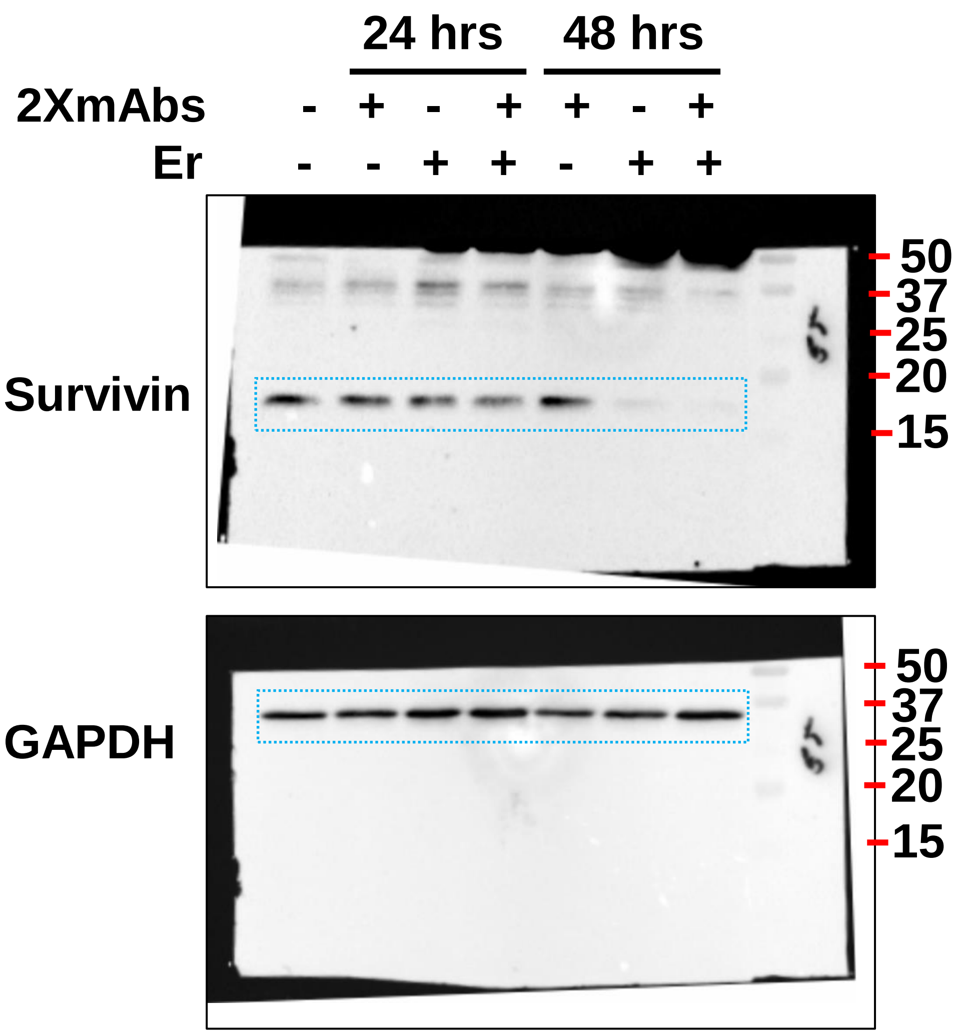
24 hrs
48 hrs
2XmAbs - + - + + - +
Er - - + + - + +
Survivin
GAPDH
50
37
25
20
15
50
37
25
20
15
